# Identifying critical lysines in mammalian histone H3 with high-throughput CRISPR prime editing

**DOI:** 10.1038/s41588-026-02675-y

**Published:** 2026-07-08

**Authors:** Daniel Price, Grigory Zemlyanskiy, Watanya Trakarnphornsombat, Ignasi Forne, Nadezda Volkova, Louis Dubusse, Alexandra J. Cooper, Axel Imhof, Aliaksandra Radzisheuskaya

**Affiliations:** 1 Division of Cell and Molecular Biology, The Institute of Cancer Research, London, UK; 2 Protein Analysis Unit and Department of Molecular Biology, Biomedical Center Munich, Faculty of Medicine, LMU Munich, Martinsried, Germany

**Keywords:** Epigenetics, Mutagenesis, Epigenetics, Genetic engineering

## Abstract

Histone post-translational modifications are fundamental to genome regulation, yet dissecting the functions of individual histone marks in mammals remains challenging due to the presence of multiple histone gene copies. Here we develop a high-throughput clustered regularly interspaced short palindromic repeats (CRISPR) prime editing platform enabling precise, reversible and combinatorial mutagenesis of canonical and noncanonical histone H3 genes within their native genomic context. Using systematic lysine-to-arginine substitutions benchmarked against synonymous controls, we identify key residues, including H3K4, H3K9, H3K14, H3K18 and H3K79, whose mutation compromises fitness in mouse embryonic stem cells. We further show that H3K56, linked to genome stability in yeast and *Drosophila*, has a conserved role in mammalian cells. Through analysis of selected double mutants, we uncover functional crosstalk across residues, with combinations such as H3K27R + H3K36R impairing stem cell self-renewal and altering transcription. Altogether, this study establishes a functional map of histone H3 lysines in mammals and provides a broadly applicable platform for systematic dissection of chromatin regulation.

## Abstract (web summary)

This study uses a precise and efficient clustered regularly interspaced short palindromic repeats (CRISPR) prime editing system to substitute lysine residues in histone H3, individually or in combination, identifying those essential for mouse embryonic stem cell self-renewal.

## Main

Chromatin states are partly regulated by covalent post-translational modifications (PTMs) of histones, which influence chromatin structure and recruitment of regulatory factors. Despite extensive profiling, the functional contribution of many individual PTMs remains unclear, because much of our understanding relies on perturbation of the enzymes that deposit these marks. Interpreting such studies is frequently complicated by redundancy, substrate pleiotropy and noncatalytic functions of histone-modifying enzymes^1–4^. Therefore, mutating individual histone residues provides an orthogonal approach for dissecting PTM functions.

Large-scale histone mutagenesis was first conducted in *Saccharomyces cerevisiae*^5,6^, where genetic manipulation is facilitated by the presence of two redundant genes of each core histone. In multicellular organisms, histone mutagenesis is more challenging due to the expansion and diversification of histone genes during evolution. Early studies in *Drosophila* exploited the organization of all core histone genes within a single locus containing ~100 tandem repeats of a 4.8-kb unit^7^. Systematic deletion and re-introduction of mutant histone gene units in this system linked specific histone mutations to defects in viability, fertility and DNA-damage responses^8,9^.

Progress in mammalian systems has been hindered by expansion and dispersal of histone genes across four chromosomal clusters that lack regular tandem-repeat structure observed in simpler organisms^10^. Recently, clustered regularly interspaced short palindromic repeats (CRISPR) base editing was used to generate H3K27R mutant mouse embryonic stem cells (ESCs) by exploiting conserved sequence surrounding the K27 codon in canonical histone H3 genes^11^. However, base editing is constrained by a narrow editing window, bystander mutations and limited control over the amino acid substitutions introduced, reducing its applicability across histone residues.

To overcome these limitations, we applied CRISPR prime editing^12^, which uses a Cas9 nickase–reverse transcriptase fusion and a prime editing guide RNA (pegRNA) to install precise nucleotide substitutions, small insertions and deletions without requiring DNA double-strand breaks or donor templates^12^. Using this method, we established a precise and efficient high-throughput platform for histone H3 mutagenesis in mouse ESCs. We then defined the contribution of individual H3 lysines to ESC fitness and demonstrated the utility of our method for functional interrogation of histone PTMs.

## Results

### Adapting prime editing for mammalian histone mutagenesis

Mouse canonical histone H3 is encoded by nine H3.1 and three H3.2 genes expressed in a replication-dependent manner. The noncanonical H3.3 variant is encoded by two genes (*H3f3a* and *H3f3b*) and is expressed throughout the cell cycle, being predominantly incorporated at actively transcribed and heterochromatic regions, including telomeres and pericentromeres^13^.

**Fig. 1:**
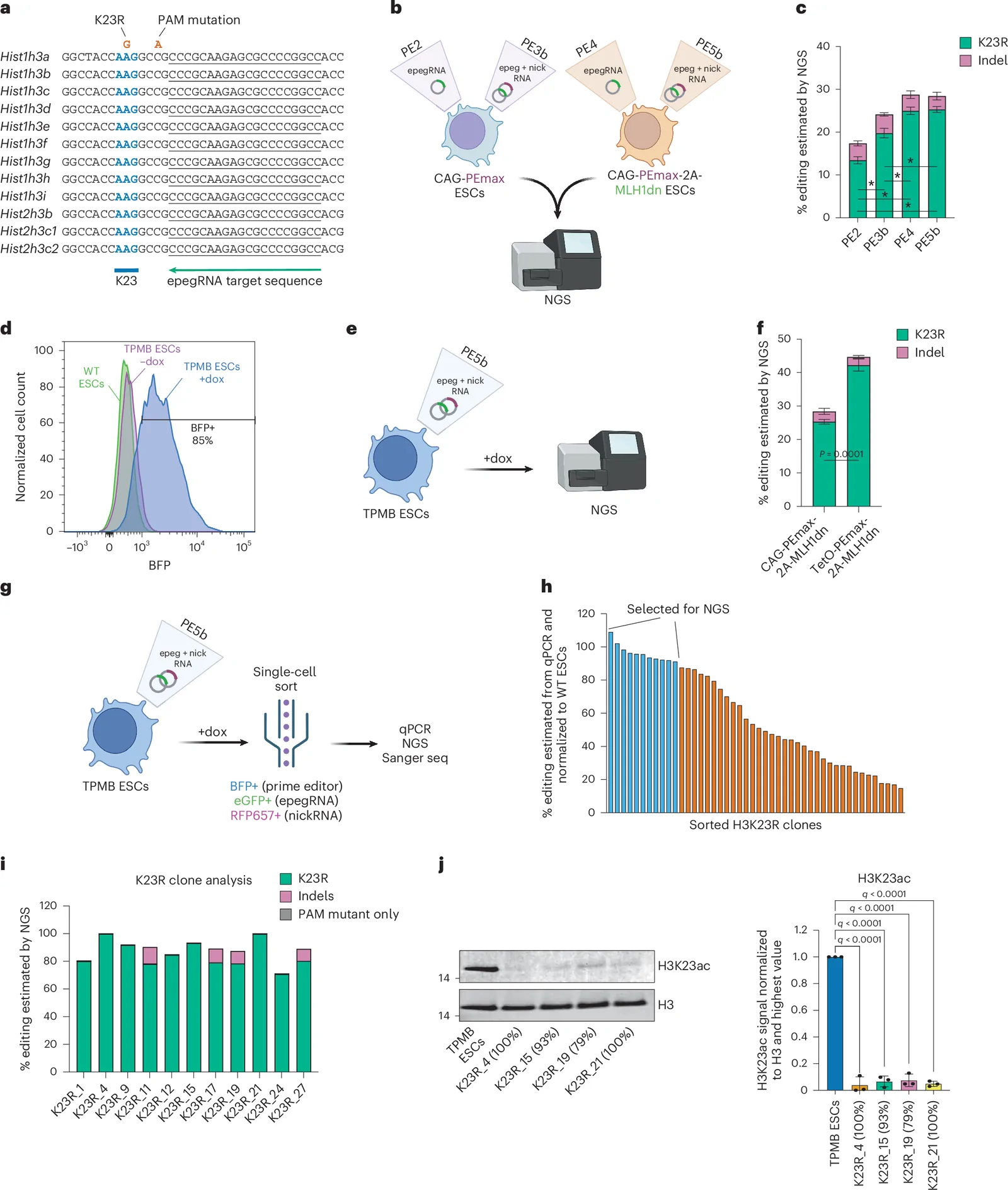
Adapting prime editing for mammalian histone mutagenesis. **a**, Nucleotide sequence of *Mus musculus* histone H3.1/H3.2 genes surrounding lysine 23, with the H3K23R epegRNA spacer and desired mutations indicated. **b**, Schematic of the prime editing systems tested. Mutagenesis is calculated using amplicon-based NGS of 18 H3.1 alleles. **c**, Bar plot showing the percentage of reads containing the H3K23R mutation or indels in the tested prime editing systems. Data are presented as mean ± 1 s.d. of *n* = 3 biological replicates (that is, independent transfections), and statistical significance assessed by one-way ANOVA followed by Tukey’s multiple comparisons test. *q < 0.05. Only significant comparisons are highlighted. **d**, Flow cytometry histogram showing BFP expression in WT and TPMB ESCs before and after the addition of doxycycline. **e**, Schematic of testing our inducible prime editing cell line, TPMB, using the PE5b system. Mutagenesis is calculated using amplicon-based NGS of 18 H3.1 alleles. **f**, Bar plot showing the percentage of reads containing the H3K23R mutation or indels in the constitutive and doxycycline-inducible PE5b system. Data are presented as mean ±1 sd. of *n* = 3 biological replicates (that is, independent transfections), and statistical significance assessed by two-tailed unpaired *t* test (*P* < 0.05). Only significant comparisons are highlighted. **g**, Schematic of establishing clonal H3K23R cell lines. PE5b components are delivered to TPMB ESCs, single-cell clones are obtained by sorting triple BFP, eGFP and RFP657 cells, and are then screened using qPCR, Sanger sequencing and amplicon-based NGS. Schematics in **b**, **e** and **g** created in BioRender; Radzisheuskaya, A. https://BioRender.com/zvoto5r (2026). **h**, Bar plot showing the percentage of target mutagenesis across canonical H3.1 in H3K23R clones estimated by qPCR. Estimations are calculated by comparing the difference in Cq values between WT and H3K23R-specific primers (ΔCq) in H3K23R clones to ΔCq values generated using synthetic DNA fragments with defined WT-to-mutant ratios (Extended Data Fig. 1d). The clones highlighted in blue were taken for subsequent amplicon-based NGS analysis. **i**, Bar plot showing the percentage of reads containing the H3K23R mutation, indels or PAM-only mutations in 11 H3K23R clones preselected using qPCR. **j**, Western blot showing the change to H3K23ac signal in the selected H3K23R clones compared to parental TPMB cells. Total H3 is used as a loading control. Quantification is presented as mean ± 1 s.d. of *n* = 3 biological replicates (that is, independent blots from independent cell pellets). Statistical significance was assessed by one-way ANOVA followed by Dunnett’s multiple comparisons test (*q* < 0.05). ANOVA, analysis of variance; Cq, quantification cycle. Source data

To establish prime editing for histone mutagenesis, we designed an enhanced pegRNA (epegRNA)^14^ targeting all 12 canonical mouse H3 genes to introduce a lysine-to-arginine (K-to-R) mutation at codon 23 (K23R) and a silent PAM-disrupting mutation (Fig. 1a). The epegRNA-expressing plasmid was transiently transfected into ESCs, which constitutively express the PEmax prime editor (PE2 system^15^). We additionally tested PE3b, which incorporates a nicking RNA^12^, PE4, which inhibits mismatch repair through expression of a dominant-negative MLH1 mutant (MLH1dn), and PE5b, which combines both strategies^15^ (Fig. 1b). Editing outcomes in the resulting cell pools were assessed by PCR across all 18 H3.1 alleles followed by amplicon-based next-generation sequencing (NGS). PE4 and PE5b, both of which inhibit mismatch repair, yielded the highest editing efficiencies and lowest indel frequencies (Fig. 1c). Although PE4 and PE5b performed similarly, we selected PE5b for subsequent experiments because previous studies reported modest improvements in efficiency and precision relative to PE4 (refs. ^15,16^).

**Extended Data Fig. 1:**
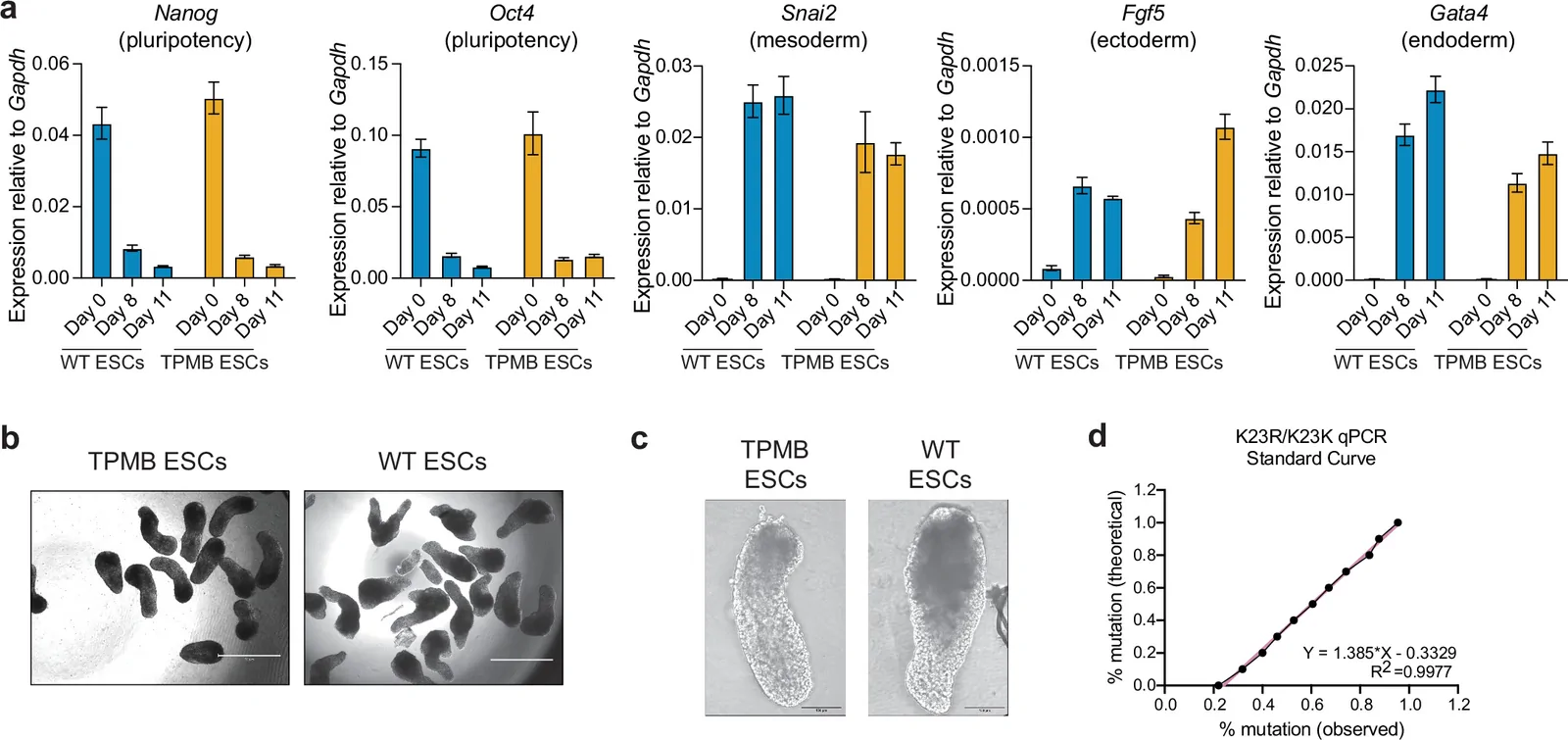
Adapting prime editing for mammalian histone mutagenesis. **a**, qRT-PCR analysis of *Nanog, Oct4, Snai2, Fgf5 and Gata4* expression in WT and TPMB ESCs in self-renewing (day 0) and differentiation (days 8–11) conditions. Differentiation was induced by stimulating embryoid body formation. The values are normalized to *Gapdh* and shown as mean ± s.d. (n = 3 technical replicates, that is, independent qRT-PCR reactions). **b**,**c**, Representative images of gastruloids derived from WT and TPMB ESCs shown at ×4 (**b**) and ×10 (**c**) magnification. Scale bar = 750 µm (**b**) and 150 µm (**c**), respectively. The experiment in (**b**) and (**c**) was performed 3 times with similar results. **d**, Standard curve generated from ΔCq values obtained by qPCR using synthetic DNA fragments mixed at defined WT-to-mutant ratios. Mutant and WT fragments were combined at varying proportions, and the difference in Cq values between WT and H3K23R-specific primers was measured to construct the curve. Source data

Because prolonged mismatch repair inhibition can promote genomic instability and mutational burden^16^, we generated a doxycycline-inducible TetO-PEmax-2A-MLH1dn-2A-BFP (TPMB) ESC line. TPMB ESCs demonstrated tight doxycycline-dependent control of prime editor expression (Fig. 1d) and superior editing efficiency using the PE5b system (Fig. 1e,f). Notably, TPMB ESCs maintained pluripotency and, upon embryoid body differentiation, expressed markers for all three germ layers (Extended Data Fig. 1a). TPMB ESCs also efficiently formed gastruloids, three-dimensional models of early gastrulation^17^ (Extended Data Fig. 1b,c), supporting their suitability for studying histone modifications.

**Extended Data Fig. 2:**
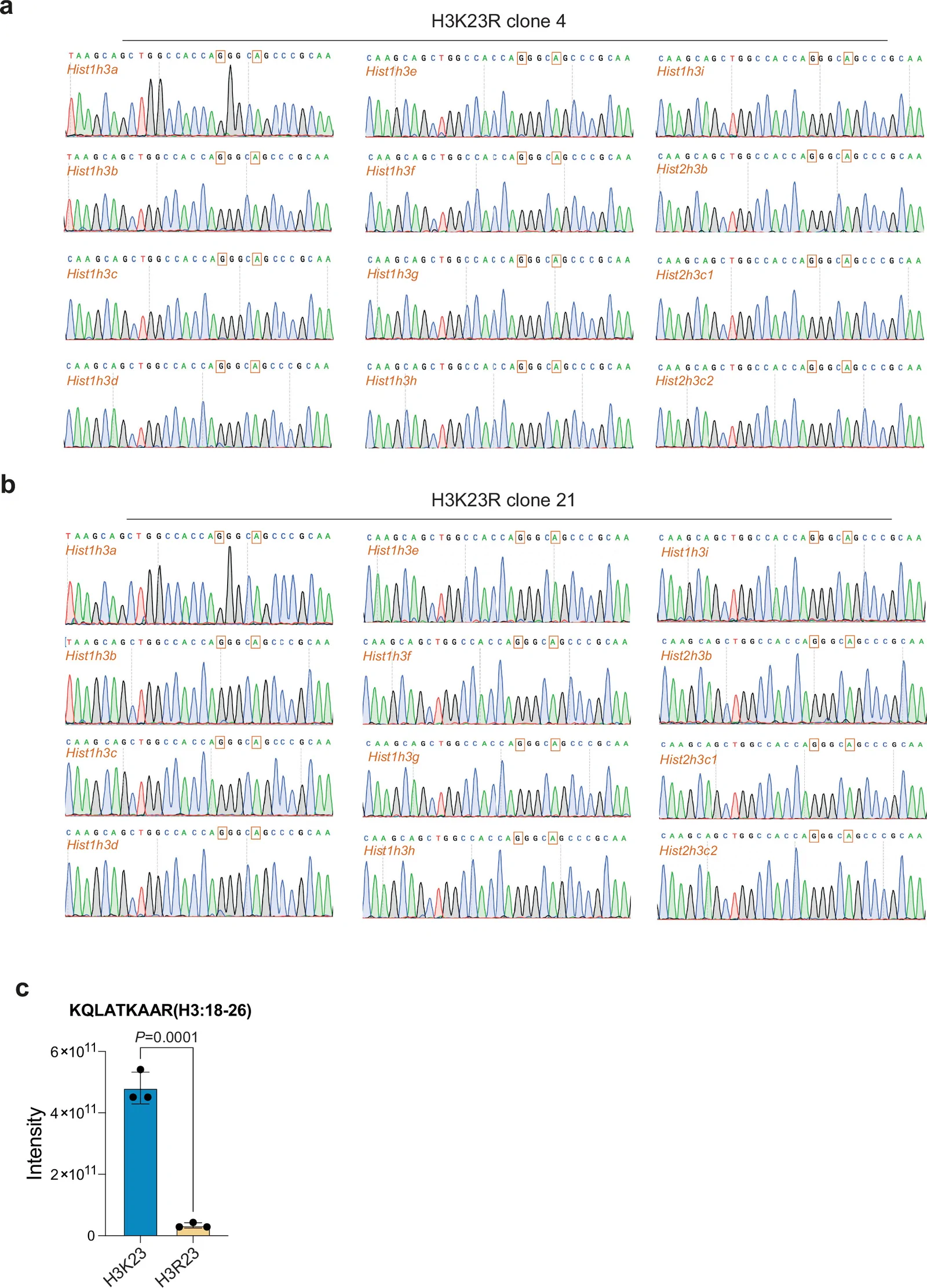
Detailed characterization of H3K23R clones. **a**,**b**, Sanger sequencing traces demonstrating successful H3K23R mutation in all the 24 histone H3.1/H3.2 genes in two independent clones (4 and 21). **c**, Signal of the KQLATKAAR(H3:18-26) peptide (all modification states were summed). Substitution of K23 with R23 eliminates the signal due to the introduction of an additional trypsin cleavage site in position 23. Quantification is presented as mean ± 1 s.d. of n = 3 biological replicates (that is, independent cell pellets). Statistical significance was assessed by a two-tailed unpaired *t*-test (*P* < 0.05). Source data

To determine whether fully edited H3K23R clonal lines could be established, TPMB ESCs were transiently transfected with epegRNA-expressing and nickRNA-expressing plasmids carrying eGFP and RFP657 reporters, respectively. After doxycycline induction, triple BFP, eGFP and RFP657 positive cells were single-cell sorted (Fig. 1g). Clones were initially screened using a qPCR assay^18^, estimating the ratio of edited-to-wild-type (WT) H3 genes. This identified high editing efficiencies, with 35% clones exceeding 80% editing (Fig. 1h and Extended Data Fig. 1d). Notably, amplicon-based NGS identified two H3K23R clones fully mutated across the 18 H3.1 alleles (Fig. 1i). Complete editing of all H3.1 and H3.2 alleles in these clones was further confirmed by individual H3 gene genotyping (Extended Data Fig. 2a,b) and mass spectrometry (MS; Extended Data Fig. 2c), indicating that amplicon-based NGS of H3.1 loci accurately reflects editing across all histone H3 alleles. H3K23R clones showed near-complete loss of H3K23ac western blot signal (Fig. 1j), demonstrating that canonical H3K23 is dispensable for ESC self-renewal.

### H3K14R and H3K18R mutations reduce cellular fitness

**Fig. 2:**
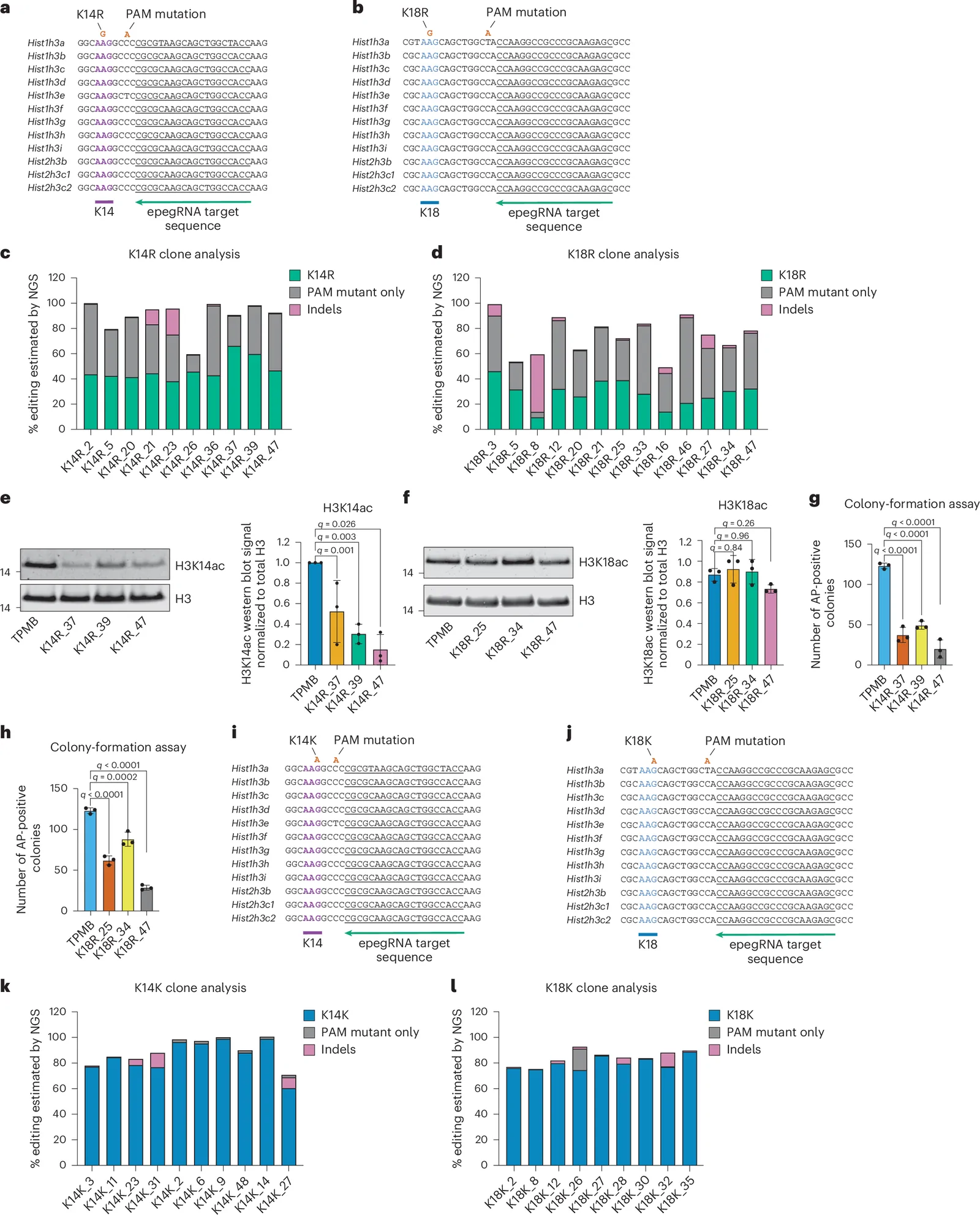
H3K14R and H3K18R mutations reduce cellular fitness. **a**,**b**, Nucleotide sequence of *M. musculus* histone H3.1/H3.2 genes surrounding lysine 14 (**a**) and lysine 18 (**b**), with the H3K14R or H3K18R epegRNA spacer and target mutations indicated. **c**,**d**, Bar plot showing the percentage of reads containing the H3K14R (**c**) or H3K18R (**d**) mutation, indels or PAM-only mutations in H3K14R or H3K18R clones preselected using qPCR. **e**,**f**, Western blot showing the change to H3K14ac signal in H3K14R clones (**e**) or H3K18ac signal in H3K18R clones (**f**) compared to parental TPMB cells. Total H3 is used as a loading control. Quantification is presented as mean ± 1 s.d. of *n* = 3 biological replicates (that is, independent blots from independent cell pellets). Statistical significance was assessed by one-way ANOVA followed by Dunnett’s multiple comparisons test (*q* < 0.05). **g**,**h**, The number of AP-positive colonies recovered after plating TPMB and H3K14R (**g**) or H3K18R (**h**) cells at clonal density. Quantification is presented as mean ± 1 s.d. of *n* = 3 biological replicates (that is, independently plated wells). Statistical significance was assessed by one-way ANOVA followed by Dunnett’s multiple comparisons test (*q* < 0.05). **i**,**j**, H3.1/H3.2 target of the H3K14K (**i**) or H3K18K (**j**) epegRNA spacer with the target mutations indicated. **k**,**l**, Bar plot showing the percentage of reads containing the H3K14K (**k**) or H3K18K (**l**) mutation, indels or PAM-only mutations in H3K14K or H3K18K clones preselected using qPCR. AP, alkaline phosphatase. Source data

Next, we applied the optimized prime editing strategy to two additional canonical H3 lysines. H3K14 and H3K18 are highly acetylated residues predominantly modified by KAT7 (HBO1) and KAT3A/KAT3B (CBP/p300), respectively^19,20^. We designed a single epegRNA for each site, leveraging the >96% sequence similarity among canonical mouse H3 genes (Fig. 2a,b). The H3K14R spacer contained a 1-bp mismatch with *Hist1h3a*, whereas *Hist1h3e* and *Hist1h3a* carried noncanonical NAG PAMs for H3K14R and H3K18R epegRNAs, respectively (Fig. 2a,b), which can still be recognized by WT Cas9 (ref. ^21^).

**Extended Data Fig. 3:**
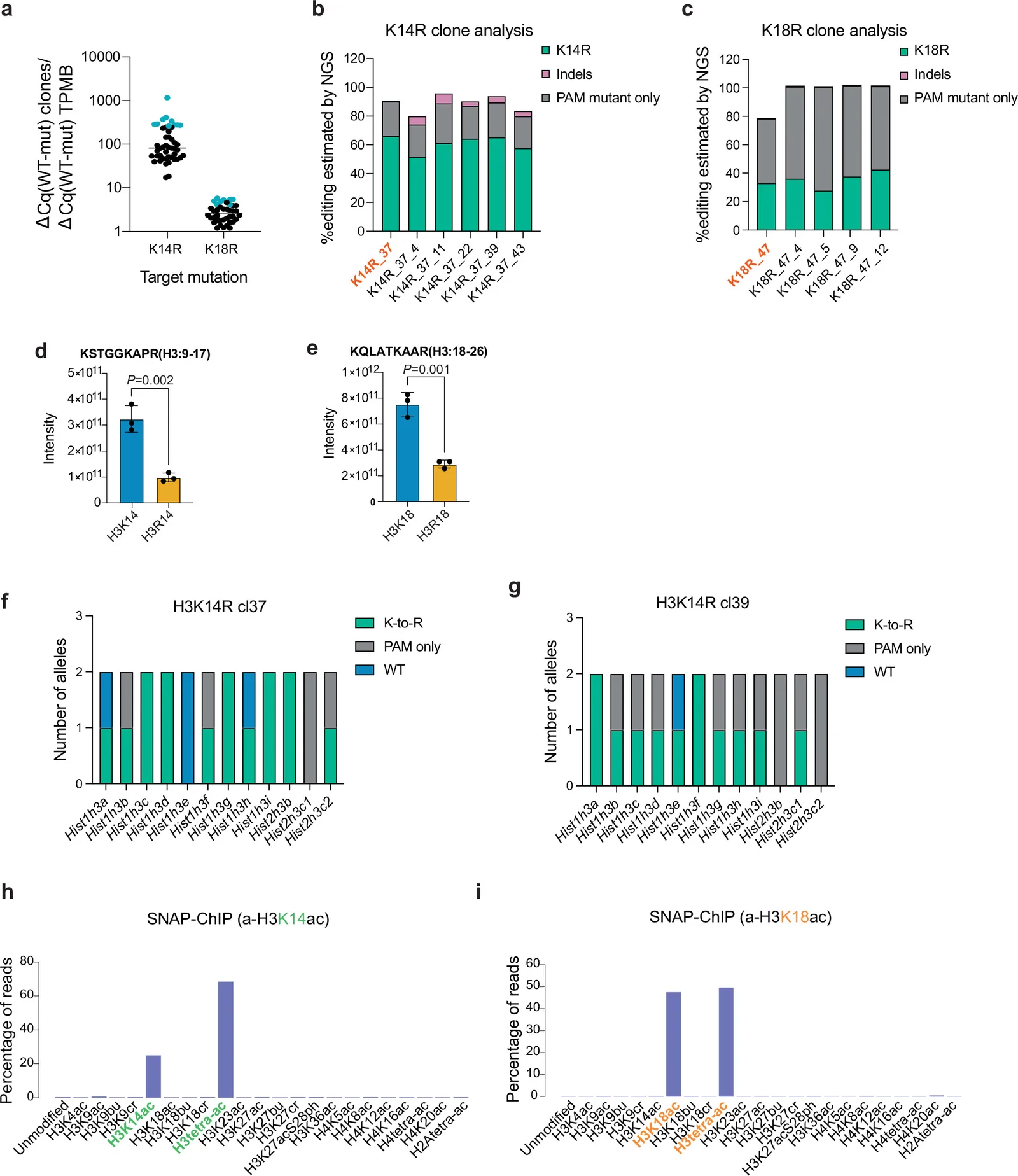
H3K14R and H3K18R mutations reduce cellular fitness. **a**, qPCR-based assessment of the H3K14R and H3K18R prime editing efficiency in 47 individual clones. The clones taken for the amplicon-based NGS analysis are highlighted in blue. **b**, Bar plot showing the % of reads containing the H3K14R mutation, indels, or PAM-only mutations in H3K14R_37 clone (K14R_37) and in the five qPCR preselected clones derived after two additional rounds of CRISPR prime editing in clone H3K14R_37. **c**, Bar plot showing the % of reads containing the H3K18R mutation, indels, or PAM-only mutations in H3K18R_47 clone (K18R_47) and in four qPCR preselected clones derived after two additional rounds of CRISPR prime editing in clone H3K18R_47. **d**, Signal of the KSTGGKAPR(H3:9-17) peptide (all modification states were summed). Partial substitution of K14 with R14 decreases the signal due to the introduction of an additional trypsin cleavage site in position 14. Quantification is presented as mean ± 1 s.d. of n = 3 biological replicates (that is, independent cell pellets). Statistical significance was assessed by a two-tailed unpaired *t*-test (*P* < 0.05). **e**, Signal of the KQLATKAAR(H3:18-26) peptide (all modification states were summed). Partial substitution of K18 with R18 decreases the signal due to the introduction of an additional trypsin cleavage site in position 18. Quantification is presented as mean ± 1 s.d. of n = 3 biological replicates (that is, independent cell pellets). Statistical significance was assessed by a two-tailed unpaired *t*-test (*P* < 0.05). **f**,**g**, Bar plots summarizing individual histone H3 genes genotyping in two independent H3K14R clones. **h**,**i**, SNAP-ChIP antibody specificity validation results for anti-H3K14ac (**h**) and anti-H3K18ac (**i**) antibodies, data shown as a percentage of total aligned reads (H3 tetra contains H3K4/9/14/18ac). Source data

Notably, incorporation of the desired mutation at both residues was markedly less efficient than at H3K23, and no fully mutant clones were obtained (Fig. 2c,d). Mutagenesis of H3K14 was below 67% (~16/24 alleles; Fig. 2c), whereas H3K18R editing did not exceed 50% (Fig. 2d). These maximal editing efficiencies were consistently achieved after multiple (two to three) rounds of epegRNA/nickRNA transfection and qPCR prescreening of 47 clones per condition (Extended Data Fig. 3a). Additional transfections of partially edited H3K14R and H3K18R clones failed to increase editing efficiency further (Extended Data Fig. 3b,c). Partial editing of these sites was confirmed by MS (Extended Data Fig. 3d,e).

Notably, partially edited H3K14R and H3K18R clones showed a high frequency of alleles carrying only the PAM-disrupting mutation without the intended K-to-R substitution (Fig. 2c,d), indicating that reduced mutagenesis was unlikely due to low prime editing activity. Instead, these data suggest a biological constraint on the number of H3K14R and H3K18R alleles tolerated by ESCs, with continued mutagenesis leading to accumulation of incomplete editing products.

Individual genotyping of H3 genes in two partial H3K14R clones revealed no gene-specific editing bias (Extended Data Fig. 3f,g), including editing of *Hist1h3e* despite its NAG PAM and *Hist1h3a* despite the spacer mismatch. Using antibodies which we validated by sample normalization and antibody profiling chromatin immunoprecipitation (SNAP–ChIP)^22^ (Extended Data Fig. 3h,i), we found that partial H3K14 mutagenesis led to more than 50% decrease in H3K14ac western blot signal (Fig. 2e), whereas changes to H3K18ac signal were minor in H3K18R partial mutants (Fig. 2f), potentially reflecting compensation by remaining WT alleles. Partially edited H3K14R and H3K18R mutants exhibited impaired self-renewal capacity in colony-formation assays (Fig. 2g,h), which supports the negative impact of these mutations on cellular fitness.

To test whether the inability to generate fully mutant H3K14R and H3K18R cells reflected a biological constraint, we designed control epegRNAs introducing silent lysine-to-lysine (K-to-K) substitutions at codon 14 (Fig. 2i) or 18 (Fig. 2j) but otherwise identical to the corresponding K-to-R epegRNAs. In contrast to K-to-R editing, K-to-K mutagenesis produced predominantly highly edited clones, reaching 70–100% editing at H3K14 and 75–90% editing at H3K18, with few PAM-only alleles (Fig. 2k,l). Together, these findings suggest that H3K14R and H3K18R mutations confer a fitness defect, leading to outcompetition by less-edited ESCs.

### Prime editing enables reversible and combinatorial histone mutation

**Fig. 3:**
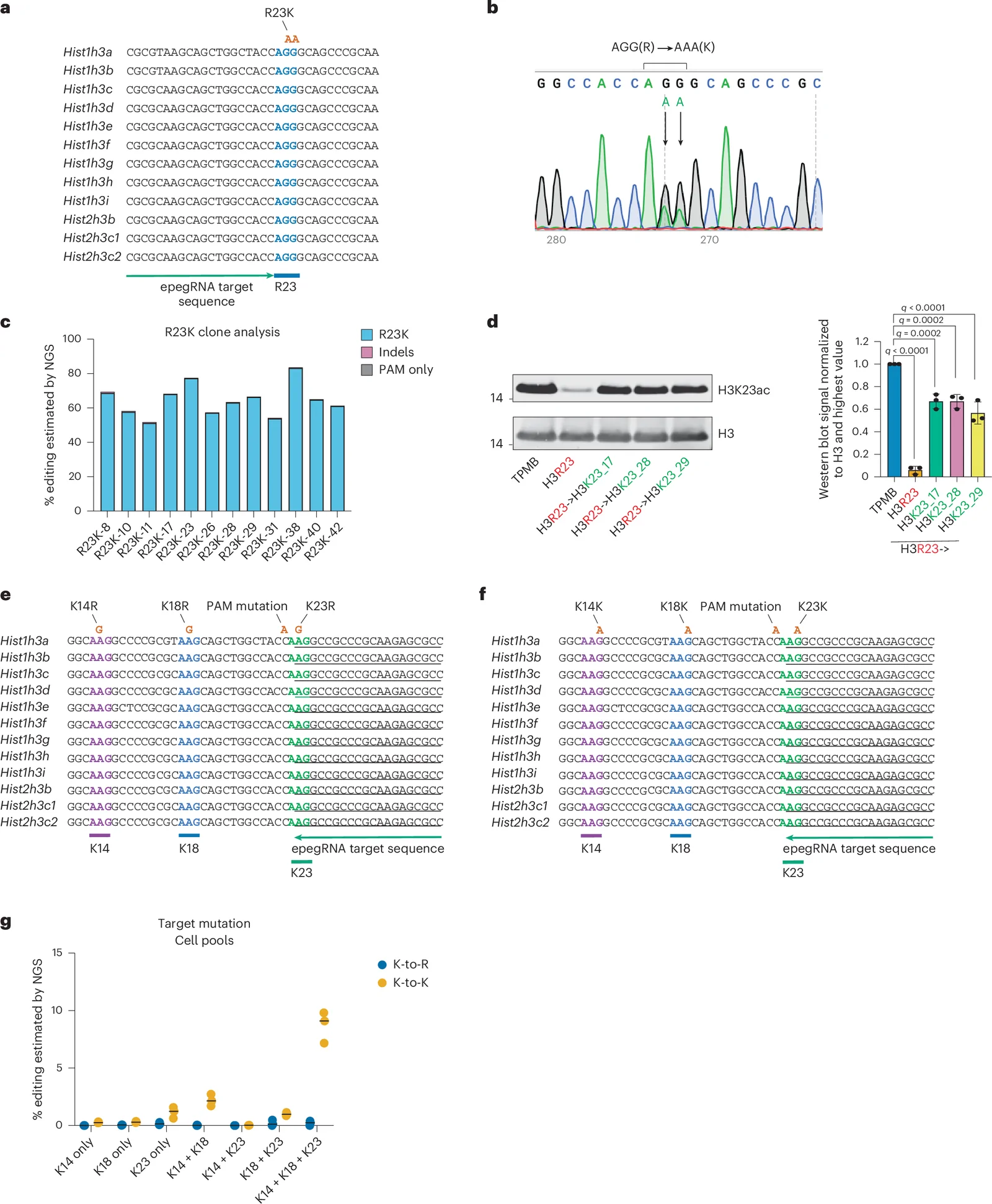
Prime editing enables reversible and combinatorial histone mutation. **a**, Nucleotide sequence of *M. musculus* histone H3.1/H3.2 genes surrounding installed arginine 23, with the H3R23K epegRNA spacer and target mutations indicated. PAM mutation in this strategy will be introduced by the target mutation. **b**, Sanger sequencing trace demonstrating the efficient emergence of AGG-to-AAA (R-to-K) mutation in the pool of transfected cells. **c**, Bar plot showing the percentage of reads containing the H3R23K mutation or indels in 12 H3R23K clones preselected using qPCR. **d**, Western blot showing H3K23ac levels in parental TPMB ESCs, fully mutant H3K23R clone and H3R23K reverted clones derived from it. Total H3 is used as a loading control. Quantification is presented as mean ± 1 s.d. of *n* = 3 biological replicates (that is, independent blots from independent cell pellets). Statistical significance was assessed by one-way ANOVA followed by Dunnett’s multiple comparisons test (*q* < 0.05). **e**, Nucleotide sequence of *M. musculus* histone H3.1/H3.2 genes surrounding lysine 14 to lysine 23, with the triple K-to-R epegRNA spacer and target mutations indicated. **f**, Nucleotide sequence of *M. musculus* histone H3.1/H3.2 genes surrounding lysine 14 to lysine 23, with the triple K-to-K epegRNA spacer and target mutations indicated. **g**, Scatterplot showing the percentage of reads containing single, double or triple K14, K18, K23 mutations in the pools of transfected cells (*n* = 3 biological replicates, that is, independent cell transfections). Source data

We then tested whether introduced histone mutations could be reverted. We designed an epegRNA/nickRNA pair programmed to restore lysine at codon 23 in fully mutant H3K23R ESCs (Fig. 3a). Transient transfection produced efficient editing in bulk cell populations (Fig. 3b). Single-cell cloning yielded highly reverted lines (Fig. 3c) with restored H3K23ac western blot signal (Fig. 3d), demonstrating that prime editing enables reversible histone mutagenesis.

Next, we asked whether the system supports combinatorial histone editing. To test this, we designed an epegRNA encoding simultaneous K-to-R or synonymous K-to-K mutations at H3K14, H3K18 and H3K23 across a 28-bp editing window containing four nucleotide substitutions (Fig. 3e,f). We found efficient installation of synonymous triple-K mutations, whereas triple K-to-R editing was markedly reduced (Fig. 3g), consistent with fitness defects associated with H3K14R and H3K18R mutations. Triple-K editing efficiencies were approximately fourfold to fivefold lower than those observed for single-residue mutagenesis.

### Pan-H3 mutagenesis uncovers histone variant compensation

**Fig. 4:**
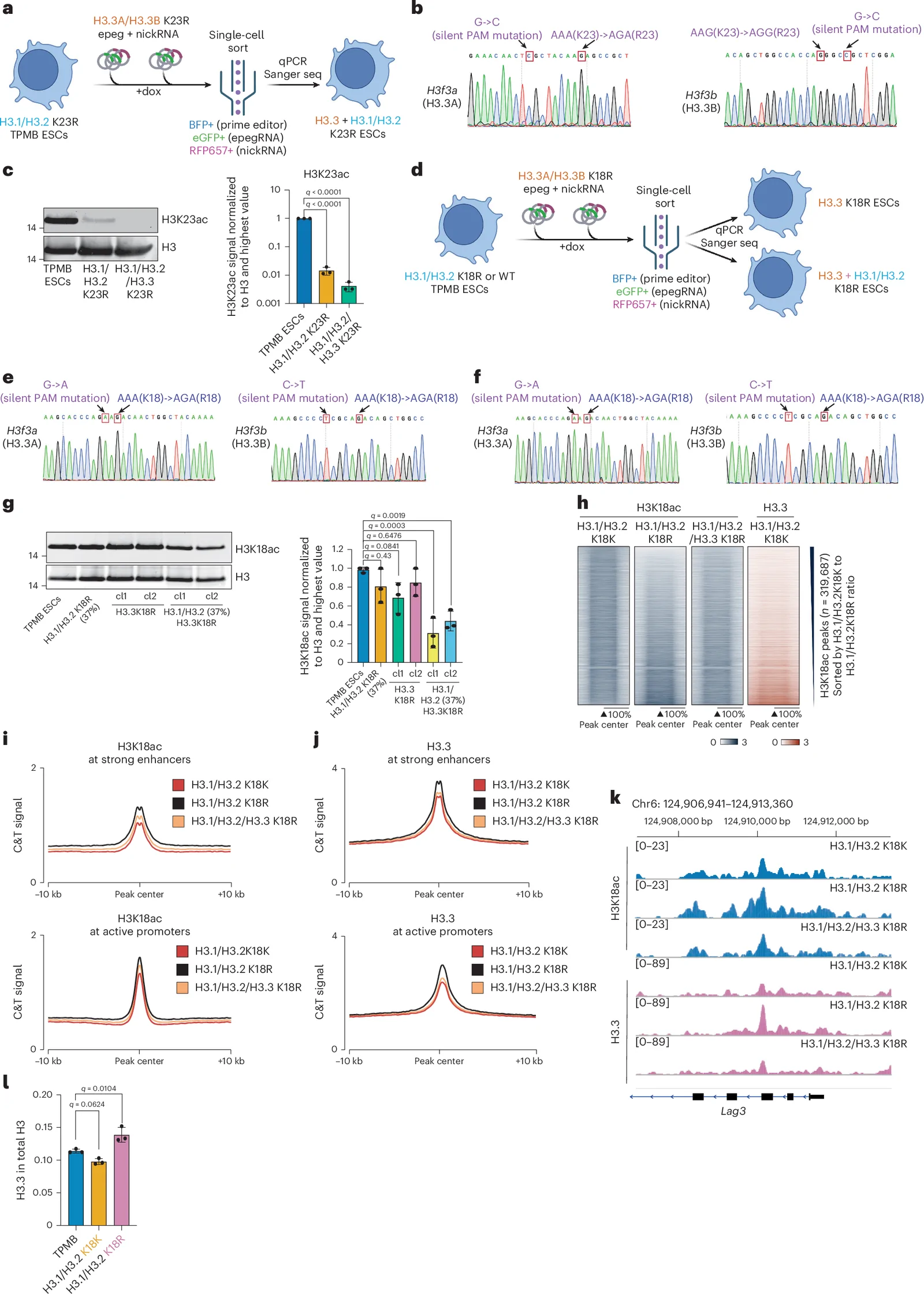
Pan-H3 mutagenesis uncovers histone variant compensation. **a**, Schematic of establishing clonal H3.1/H3.2/H3.3K23R cell lines. H3.3A/H3.3B epegRNA/nickRNAs are delivered to H3.1/H3.2K23R ESCs, single-cell clones are obtained by sorting triple BFP, GFP and RFP657 cells and are then screened using qPCR and Sanger sequencing. **b**, Sanger sequencing traces demonstrating the complete K23R and PAM mutations at both *H3f3a* and *H3f3b* genes. **c**, Western blot showing the change to H3K23ac signal in H3.1/H3.2K23R and H3.1/H3.2/H3.3K23R clones compared to parental TPMB cells. Total H3 is used as a loading control. Quantification is presented as mean ± 1 s.d. of *n* = 3 biological replicates (that is, independent blots from independent cell pellets). Statistical significance was assessed by one-way ANOVA followed by Dunnett’s multiple comparisons test (*q* < 0.05). **d**, Schematic of establishing clonal H3.1/H3.2/H3.3K18R and H3.3K18R cell lines. H3.3A/H3.3B epegRNA/nickRNAs are delivered to partial H3.1/H3.2K18R or TPMB ESCs, single-cell clones are obtained by sorting triple BFP, GFP and RFP657 cells, and are then screened using qPCR and Sanger sequencing. Schematics in **a** and **d** created in BioRender; Radzisheuskaya, A. https://BioRender.com/zvoto5r (2026). **e**,**f**, Sanger sequencing traces demonstrating the complete K18R and PAM mutations at both *H3f3a* and *H3f3b* genes in TPMB (**e**) or partial H3.1/H3.2K18R (**f**) cells. **g**, Western blot showing the change to H3K18ac signal in H3.1/H3.2/H3.3K18R and H3.3K18R clones compared to parental TPMB cells. Total H3 is used as a loading control. Quantification is presented as mean ± 1 s.d. of *n* = 3 biological replicates (that is, independent blots from independent cell pellets). Statistical significance was assessed by one-way ANOVA followed by Dunnett’s multiple comparisons test (*q* < 0.05). **h**, Heatmaps of H3K18ac C&T signal in H3.1/H3.2K18K, H3.1/H3.2K18R and H3.1/H3.2/H3.3K18R cells and of H3.3 C&T signal in the control H3.1/H3.2K18K cells across all the H3K18ac peaks (the peaks are shown as length normalized). The experiment was performed in three biological replicates (that is, independent cell pellets). **i**, Average profiles of H3K18ac C&T signal across all strong enhancers (top) and active promoters (bottom) in H3.1/H3.2K18K, H3.1/H3.2K18R and H3.1/H3.2/H3.3K18R cells. The experiment was performed in three biological replicates (that is, independent cell pellets). **j**, Average profiles of H3.3 C&T signal across all strong enhancers (top) and active promoters (bottom) in H3.1/H3.2K18K, H3.1/H3.2K18R and H3.1/H3.2/H3.3K18R cells. The experiment was performed in three biological replicates (that is, independent cell pellets). **k**, H3K18ac and H3.3 C&T signals in H3.1/H3.2K18K, H3.1/H3.2K18R and H3.1/H3.2/H3.3K18R cells at a representative locus. **l**, MS-based quantification of H3.3-to-H3.1/H3.2 ratio in the chromatin of TPMB, H3.1/H3.2K18K and H3.1/H3.2K18R cells based on the intensity of the unmodified peptides 27–40, which differs between H3.1/H3.2 and H3.3 by the presence of S31 in H3.3. Quantification is presented as mean ± 1 s.d. of *n* = 3 biological replicates (that is, independent cell pellets). Statistical significance was assessed by one-way ANOVA followed by Dunnett’s multiple comparisons test (*q* < 0.05). Source data

Although H3.3 is dispensable for ESC viability^23^, it may compensate for mutations in canonical H3.1/H3.2. We therefore tested whether H3.3 could be efficiently mutated using our system. Because sequence conservation between the two H3.3 genes is substantially lower than among canonical H3 genes, separate epegRNA/nickRNA pairs were required for each locus. Simultaneous transfection of both targeting pairs into fully mutant H3.1/H3.2K23R ESCs, followed by single-cell sorting and clonal analysis, generated pan-H3K23R mutants (Fig. 4a,b), which showed complete loss of residual H3K23ac western blot signal (Fig. 4c).

Because partially edited H3.1/H3.2K18R mutants retained near-normal H3K18ac levels (Fig. 2f), we hypothesized that H3.3 may compensate for the loss of canonical H3K18ac. To test this, we introduced H3.3K18R mutations into both WT and partially edited H3.1/H3.2K18R ESCs (Fig. 4d). Complete mutagenesis of *H3f3a* and *H3f3b* was achieved in both genetic backgrounds (Fig. 4e,f). Introduction of H3.3K18R into a partial H3.1/H3.2K18R mutant resulted in a reduction in H3K18ac levels (Fig. 4g), supporting partial compensation by H3.3 for the loss of canonical H3K18ac.

To further investigate this compensatory mechanism, we performed CUT&Tag (C&T) profiling of H3.3 and H3K18ac levels in WT (H3.1/H3.2K18K), H3.1/H3.2K18R and H3.1/H3.2/H3.3K18R ESCs. H3.1/H3.2K18R mutant exhibited marked redistribution of H3K18ac across the genome (Fig. 4h). Regions showing increased H3K18ac strongly correlated with H3.3 localization, whereas depletion of H3K18ac was largely confined to H3.3-poor regions. Introduction of H3.3K18R into partially edited H3.1/H3.2K18R cells abolished this redistribution and resulted in a genome-wide mild reduction in H3K18ac levels (Fig. 4h).

We then examined enhancer and promoter regions, which are typically enriched for H3.3 (refs. ^24–26^). At both classes of regulatory elements, increased H3K18ac in H3.1/H3.2K18R cells was accompanied by increased H3.3 incorporation, an effect that was lost upon introduction of the H3.3K18R mutation (Fig. 4i–k). Consistent with these findings, MS analysis revealed increased H3.3-to-H3.1/H3.2 ratios in H3.1/H3.2K18R cells relative to WT ESCs (Fig. 4l), providing independent evidence of histone variant compensation.

### Genomic by-products of histone mutagenesis by prime editing

**Fig. 5:**
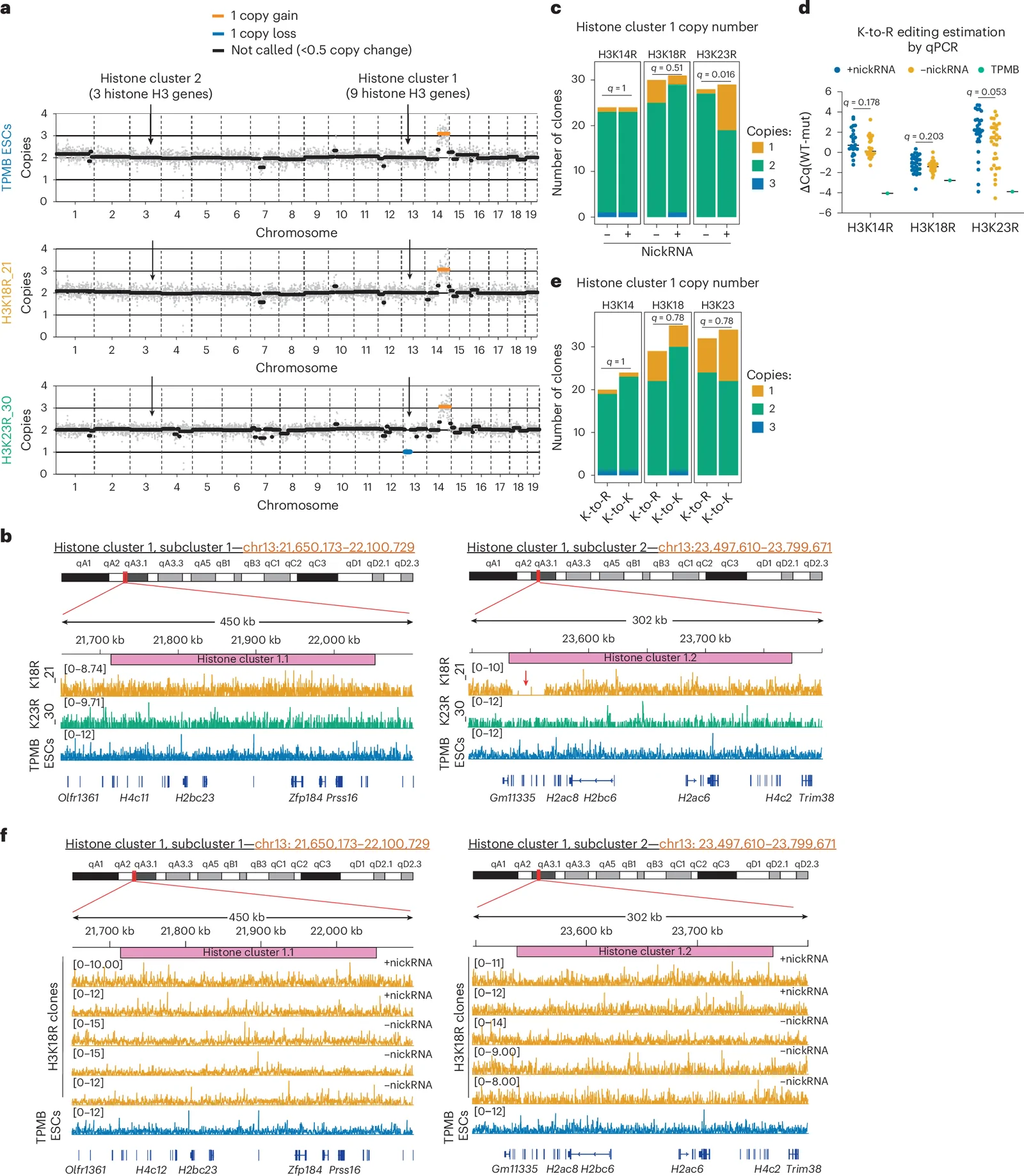
Genomic by-products of histone mutagenesis by prime editing. **a**, Chromosomal copy number plot showing large-scale amplifications or deletions in TPMB ESCs and two histone mutant clones—H3K18R_21 and H3K23R_30. Histone clusters are indicated in chromosomes 3 and 13, and called copy number changes are highlighted. Parental TPMB ESCs exhibited a partial duplication of chromosome 14, inherited by all the clones. This, however, does not compromise their pluripotency as shown in Extended Data Fig. 1. **b**, Shallow whole-genome sequencing profiles over histone cluster 1, subclusters 1 and 2 for TPMB ESCs, and H3K18R_21 and H3K23R_30 clones. A 25-kb deletion in the H3K18R_21 clone is highlighted with a red arrow. **c**, Histone cluster 1 copy number frequencies in H3K14R, H3K18R and H3K23R clones generated with or without nickRNA. qPCR-based copy number estimates were categorized as 1 (values < 1.5), 2 (values ≥ 1.5 and <2.5) or 3 (values ≥ 2.5) copies, and differences across groups were assessed using two-sided Fisher’s exact test followed by Benjamini–Hochberg multiple comparisons test (*q* < 0.05). More than 20 independent clonal cell lines were assessed for each condition. **d**, qPCR-based assessment of the H3K18R and H3K23R prime editing efficiency in the presence or absence of nickRNA. The statistical significance was assessed using two-tailed unpaired *t* tests with Benjamini–Krieger–Yekutieli correction for multiple comparisons (*q* < 0.05). More than 20 independent clonal cell lines were assessed for each condition. **e**, Histone cluster 1 copy number frequencies in H3K14K/H3K14R, H3K18K/H3K18R and H3K23K/H3K23R clones generated with nickRNA. qPCR-based copy number estimates were categorized as 1 (values < 1.5), 2 (values ≥ 1.5 and <2.5) or 3 (values ≥ 2.5) copies, and differences across groups were assessed using two-sided Fisher’s exact test followed by Benjamini–Hochberg multiple comparisons test (*q* < 0.05). More than 20 independent clonal cell lines were assessed for each condition. **f**, Shallow whole-genome sequencing profiles over histone cluster 1, subclusters 1 and 2 for TPMB ESCs, and five H3K18R clones generated with or without nickRNA. Source data

**Extended Data Fig. 4:**
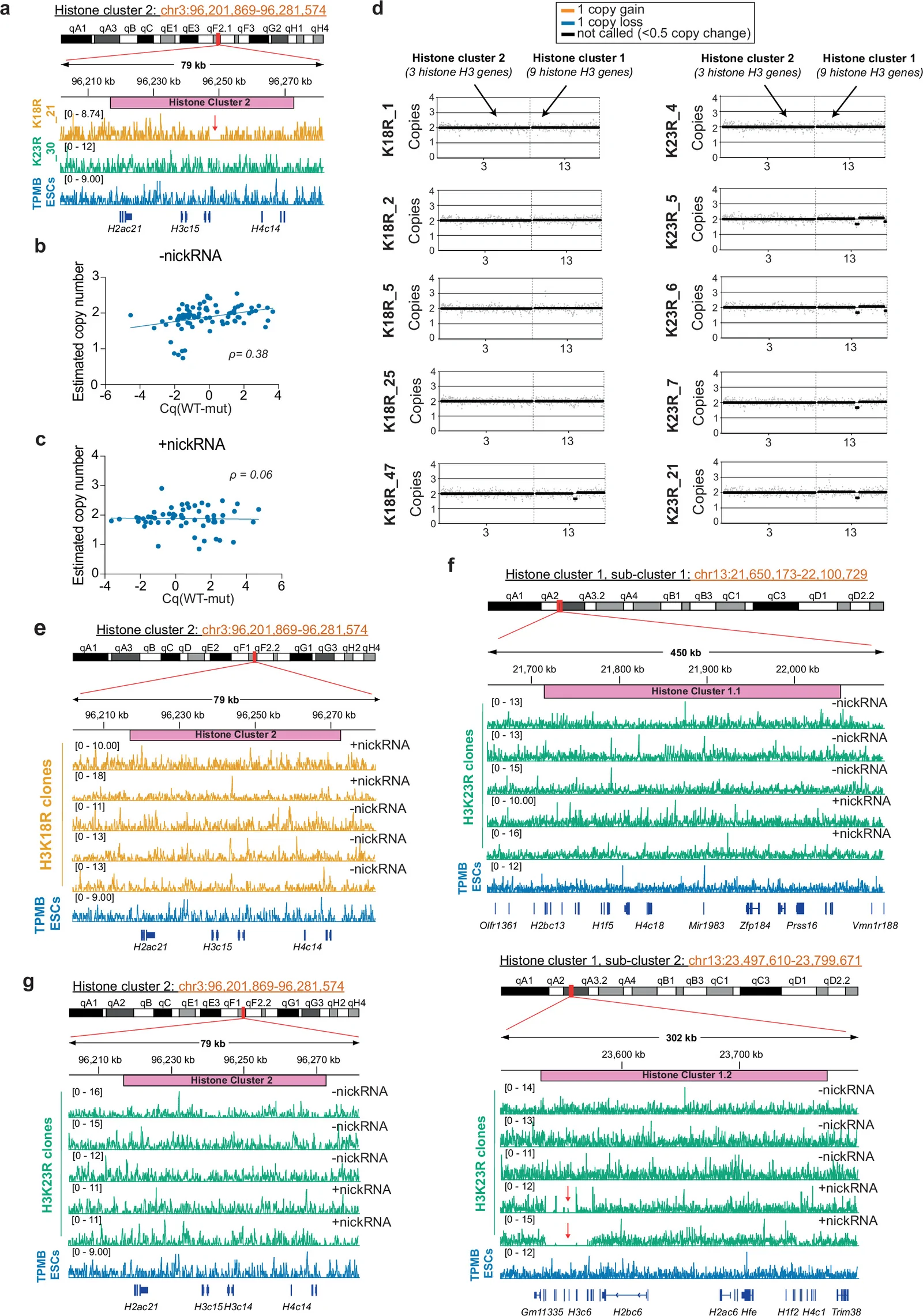
Genomic by-products of histone mutagenesis by prime editing. **a**, Shallow whole-genome sequencing profiles over histone cluster 2 for TPMB ESCs, and H3K18R_21 and H3K23R_30 clones. A 3 kb deletion in the H3K18R_21 clone is highlighted with a red arrow. **b**, Scatterplot of histone cluster 1 copy number versus editing efficiency, measured by qPCR, across all K-to-R editing conditions from Fig. 5c in the absence of nickRNA. Spearman’s correlation was used to assess their association. **c**, Scatterplot of histone cluster 1 copy number versus editing efficiency, measured by qPCR, across all K-to-R editing conditions from Fig. 5c in the presence of nickRNA. Spearman’s correlation was used to assess their association. **d**, Chromosomal copy number plot in five H3K18R and five H3K23R clones preselected based on the histone cluster 1 copy number qPCR assay. Histone clusters are indicated in chromosomes 3 and 13, and called copy number changes are highlighted. **e**, Shallow whole-genome sequencing profiles over histone cluster 2 for TPMB ESCs, and five H3K18R clones generated with or without nickRNA. **f**,**g**, Shallow whole-genome sequencing profiles over histone cluster 1 subclusters 1 and 2 (**f**) and histone cluster 2 (**g**) for TPMB ESCs, and five H3K23R clones generated with or without nickRNA. Kilobase-scale deletions in two H3K23R clones are highlighted with red arrows. Source data

Because histone prime editing involves simultaneous targeting of multiple genomic loci, we assessed whether this approach induces large-scale (>1 kb) genomic alterations within histone clusters. Shallow whole-genome sequencing of several partial H3K18R and fully mutant H3K23R clones identified two classes of occasional lesions—megabase-scale loss of one copy of histone cluster 1 on chromosome 13 (Fig. 5a; clone H3K23R_30) and kilobase-scale deletions within histone clusters (Fig. 5b and Extended Data Fig. 4a; clone H3K18R_21). Previous studies have reported that large-scale deletions (>1 kb) represent common outcomes of CRISPR mutagenesis and can also occur after base or prime editing^27^. In prime editing, these events have been partly attributed to nickRNA, which can increase DNA double-strand breaks^28^.

To test whether omitting nickRNA reduces the frequency of large-scale deletions, we developed a qPCR assay to measure histone cluster 1 copy number and applied it to H3K14R, H3K18R and H3K23R mutants (*n* > 23). Depending on the condition, the frequency of histone cluster 1 copy number loss varied from 4% to 34%. The removal of nickRNA reduced copy number loss in H3K23R, but not in H3K14R or H3K18R mutants (Fig. 5c), indicating that the effect of nickRNA is locus dependent. Inclusion of nickRNA modestly increased editing efficiency at all three loci, although these effects were not statistically significant (Fig. 5d). Histone cluster 1 copy number loss did not strongly correlate with editing efficiency (Extended Data Fig. 4b,c) and occurred at similar frequencies in K-to-K and K-to-R mutagenesis reactions (Fig. 5e).

To assess the frequency of kilobase-scale deletions within histone clusters, we performed shallow whole-genome sequencing of five partially edited H3K18R and five fully mutant H3K23R clones with intact histone cluster 1 copy number by qPCR. For each genotype, two clones were generated with nickRNA and three without nickRNA. None of the ten clones showed histone cluster copy number loss (Extended Data Fig. 4d). No kilobase-scale deletions were detected in H3K18R clones (Fig. 5f and Extended Data Fig. 4e). In contrast, two H3K23R clones generated with nickRNA contained histone cluster 1 deletions ranging from 20 kb to 35 kb, with no lesions observed without nickRNA (Extended Data Fig. 4f,g).

Together, these findings show that histone prime editing can occasionally generate large-scale deletions in histone clusters. The addition of nickRNA increases the frequency of these events in a locus-specific manner. However, appropriate screening readily enables isolation of clones lacking these perturbations for downstream biological studies.

### Prime editing screen maps H3 lysine contributions to fitness

**Fig. 6:**
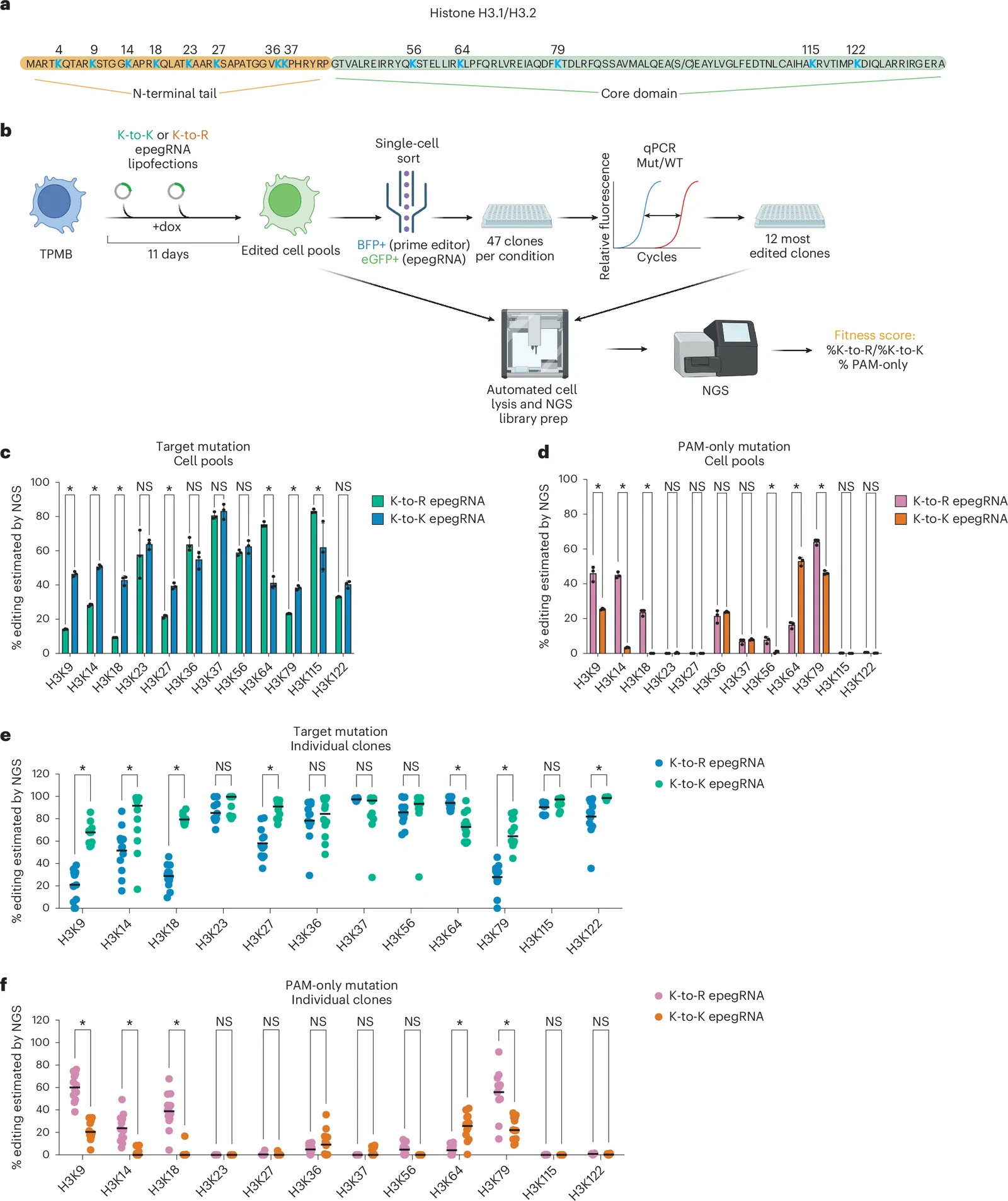
Prime editing screen maps H3 lysine contributions to fitness. **a**, Histone H3.1/H3.2 amino acid sequence. **b**, Schematic of the H3 lysine mutagenesis screen. TPMB ESCs are transfected with either K-to-R or silent K-to-K epegRNAs targeting individual H3 lysine residues. After transfection, cell pools were single-cell sorted to establish clonal lines. The clones were prescreened by qPCR and further analyzed by amplicon-based NGS. Editing outcomes from the transfected cell pools and clonal populations were compared to evaluate the impact of specific lysine mutations on cell fitness. Schematic in **b** created in BioRender; Radzisheuskaya, A. https://BioRender.com/zvoto5r (2026). **c**,**d**, Bar plots showing the percentage of reads containing the target (K-to-R or K-to-K; **c**) or PAM-only mutation (**d**) across 12 histone H3 lysines in the transfected cell pools. Quantification is presented as mean ± 1 s.d. of *n* = 3 biological replicates (that is, independent cell transfections). The statistical significance was assessed using two-tailed unpaired *t* tests with Benjamini–Krieger–Yekutieli correction for multiple comparisons. *q < 0.01. **e**,**f**, Scatterplots showing the percentage of reads containing the target (K-to-R or K-to-K; **e**) or PAM-only mutation (**f**) across 12 histone H3 lysines in the individual clonal cell lines. The statistical significance was assessed using two-tailed unpaired *t* tests with Benjamini–Krieger–Yekutieli correction for multiple comparisons. *q < 0.01. NS, nonsignificant. Source data

Next, we aimed to systematically mutate each of the 13 lysine residues across canonical H3 to assess their contribution to cellular fitness (Fig. 6a). A single epegRNA was sufficient for 11 lysine residues, whereas H3K9 required two and H3K4 required five because of sequence divergence among H3 alleles. To distinguish biological effects from editing-associated biases, reciprocal K-to-K control epegRNAs were generated for all residues. TPMB ESCs were transfected with K-to-R or K-to-K epegRNAs in biological triplicates and edited for 11 days. Transfected cell pools were analyzed by amplicon-based NGS and single-cell sorted to establish clonal lines. For each lysine, 47 independent clones were prescreened by qPCR, after which the 12 most highly edited clones underwent amplicon-based NGS (Fig. 6b). Fitness effects were inferred from differences in editing efficiency and accumulation of PAM-only mutations between K-to-R and K-to-K conditions.

**Extended Data Fig. 5:**
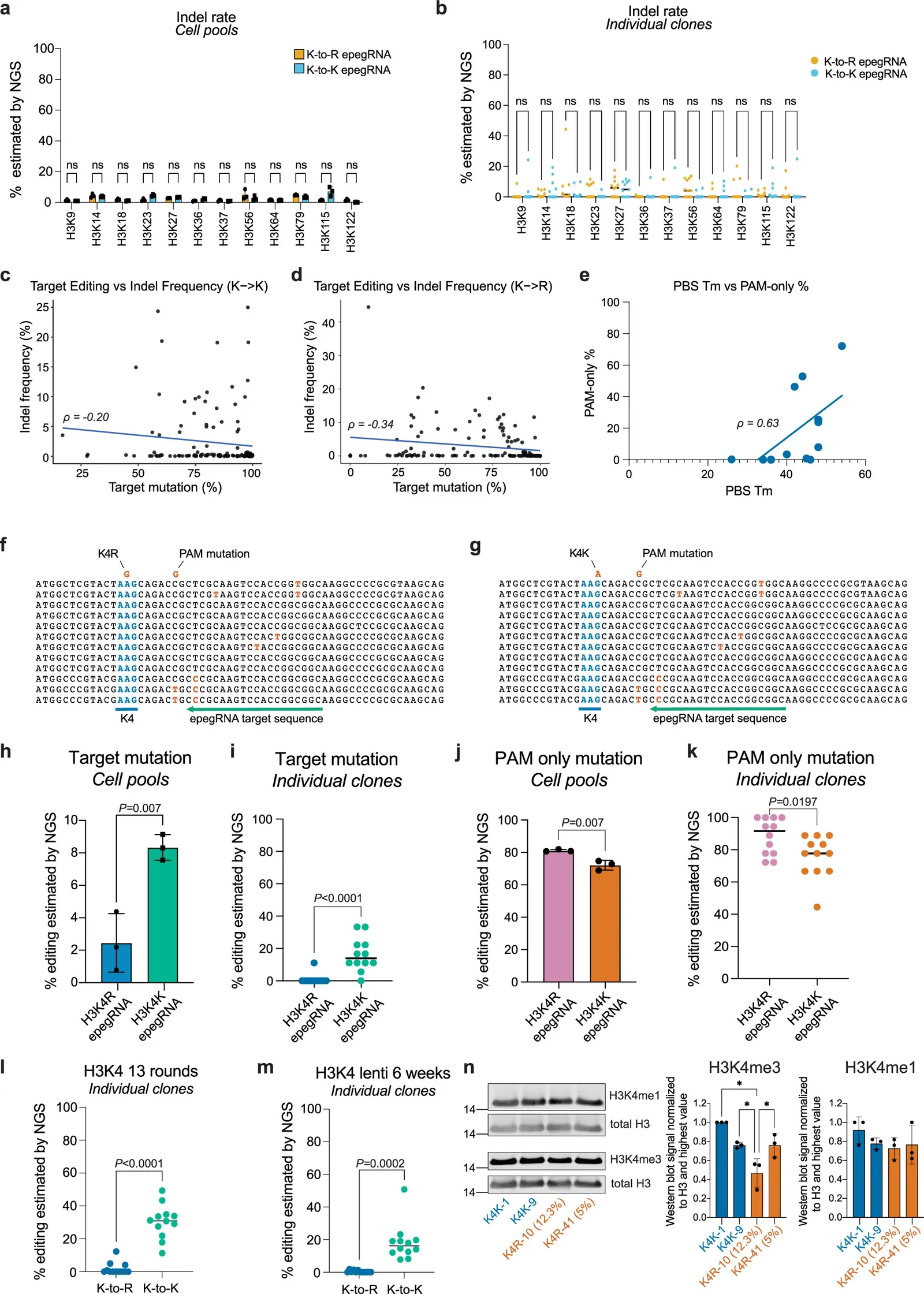
Prime editing screen maps H3 lysine contributions to fitness. **a**,**b**, Bar plot showing the % of reads with indels across 12 histone H3 lysines in transfected cell pools (n = 3 biological replicates, independent transfections) (**a**) or individual clones (**b**). Significance in **a** and **b** was assessed using two-tailed unpaired *t*-tests with Benjamini–Krieger–Yekutieli correction for multiple comparisons (* = *q* < 0.01, ns = nonsignificant). **c**,**d**, Scatterplot of indel rate versus correct edit efficiency across all K-to-K (**c**) or K-to-R (**d**) editing conditions from Fig. 6e. **e**, Scatterplot of PBS Tm versus the PAM-only mutation frequency across all K-to-K editing conditions from Fig. 6e. **f**,**g**, Sequence of *M. musculus* histone H3.1/2 genes surrounding lysine 4, showing H3K4R (**f**) and H3K4K (**g**) epegRNA spacer and intended mutations. Nonconserved bases are highlighted in red. **h**, Bar plot showing the % of reads containing the target mutation (H3K4R or H3K4K) in cell pools (n = 3 biological replicates, independent transfections). **i**, Scatterplot showing the % of reads containing the target mutation (H3K4R or H3K4K) in individual clones. **j**, Bar plot showing the % of reads containing the PAM-only mutation in the H3K4K- or H3K4R-transfected pools (n = 3 biological replicates, independent transfections). **k**, Scatterplot showing the % of reads containing the PAM-only mutation in the individual H3K4K- or H3K4R-transfected clones. **l**, Scatterplots showing the % of reads containing the target mutation (H3K4R or H3K4K) in individual clones derived from cells subjected to 13 rounds of H3K4R or H3K4K epegRNA transfection. **m**, Scatterplots showing the % of reads containing the target mutation (H3K4R or H3K4K) in individual clones derived from cells transduced with H3K4R or H3K4K epegRNA and cultured for 6 weeks. **n**, Western blot analysis of H3K4me1 and H3K4me3 levels in two H3K4K and two H3K4R clones. K-to-R editing efficiency estimated by NGS is shown in brackets. Quantification is mean ± 1 s.d. of n = 3 biological replicates (that is, independent blots from independent cell pellets). Statistical significance was assessed by one-way ANOVA followed by Tukey’s multiple comparisons test (* = *q* < 0.05). Only significant comparisons are highlighted. Spearman’s correlation was used in **c**–**e**. Statistical significance in **h**–**m** was assessed by a two-tailed unpaired *t*-test (*P* < 0.05). Source data

H3K23R editing showed no reduction in editing efficiency or accumulation of PAM-only mutations relative to K-to-K controls in either pooled or clonal analysis (Fig. 6c–f), with multiple fully edited H3K23R clones recovered. In contrast, H3K14R and H3K18R mutations showed markedly reduced editing efficiency and pronounced accumulation of PAM-only alleles (Fig. 6c–f), consistent with strong negative selection against highly edited cells. Similar phenotypes were observed for H3K9R and H3K79R, which reached maximal editing efficiencies of 39% and 46%, respectively, and showed substantial PAM-only enrichment. H3K27R showed a mildly reduced editing efficiency with no accumulation of PAM-only mutations (Fig. 6c,d), suggesting a modest proliferative defect without strong negative selection, consistent with the previous generation of pan-H3K27R ESCs^11^. H3K36R, H3K37R, H3K56R, H3K64R, H3K115R and H3K122R yielded predominantly highly or fully edited clones. H3K64R exhibited a modest gain-of-fitness phenotype, with increased editing efficiencies relative to K-to-K controls in both pooled and clonal analysis. Indel frequencies remained low across all sites (Extended Data Fig. 5a,b) and did not correlate with editing efficiency (Extended Data Fig. 5c,d).

PAM-only mutation frequencies in K-to-K conditions varied across lysine sites. To investigate this locus-specific variation, we analyzed epegRNA extension length, PBS length, reverse transcription template (RTT) length, PBS GC content, PBS melting temperature, predicted on-target score, PAM codon identity and usage frequency, PAM position in the RTT and target edit position in the RTT. Only PBS melting temperature showed a moderate-to-strong correlation with PAM-only frequency (Extended Data Fig. 5e). Higher PBS melting temperature may increase the frequency of reverse transcription initiation, which, particularly under conditions of mismatch repair inhibition, could increase the generation of incomplete or abortive prime editing products. PegRNA folding, local sequence or chromatin context may additionally contribute.

H3K4 mutagenesis required a multiplex editing strategy using five epegRNAs to account for sequence divergence among H3 alleles (Extended Data Fig. 5f,g). Unlike other lysine residues, synonymous K-to-K editing at H3K4 was inefficient, reaching only ~8% editing in cell pools and up to 33% in clonal lines (Extended Data Fig. 5h,i). K-to-R editing was dramatically reduced and associated with increased accumulation of PAM-only mutations in both pooled and clonal analyses (Extended Data Fig. 5j,k).

Because each H3K4 epegRNA perfectly matches only a subset of H3 alleles, the low editing efficiency and high frequency of incomplete editing products (Extended Data Fig. 5j,k) may result from nonproductive target engagement or inefficient PBS annealing at noncognate H3 loci. To improve H3K4 editing, we removed the PAM-disrupting mutation and extended the editing window either through repeated transfections (13 rounds) or through long-term lentiviral epegRNA expression (6 weeks; Extended Data Fig. 5l,m). These modifications improved K-to-K editing efficiency, with repeated transfection increasing median editing from 13.9% to 31% and maximum editing from 33% to 51% (Extended Data Fig. 5m). In contrast, K-to-R editing remained extremely rare, with only 2 of 36 clones reaching 11–12% editing (Extended Data Fig. 5j,m), showing no detectable changes in H3K4me1 and a modest reduction in H3K4me3 in the more edited clone (Extended Data Fig. 5n). These findings indicate strong negative selection against H3K4R cells.

### Histone mutagenesis induces loss of PTMs and functional effects

**Fig. 7:**
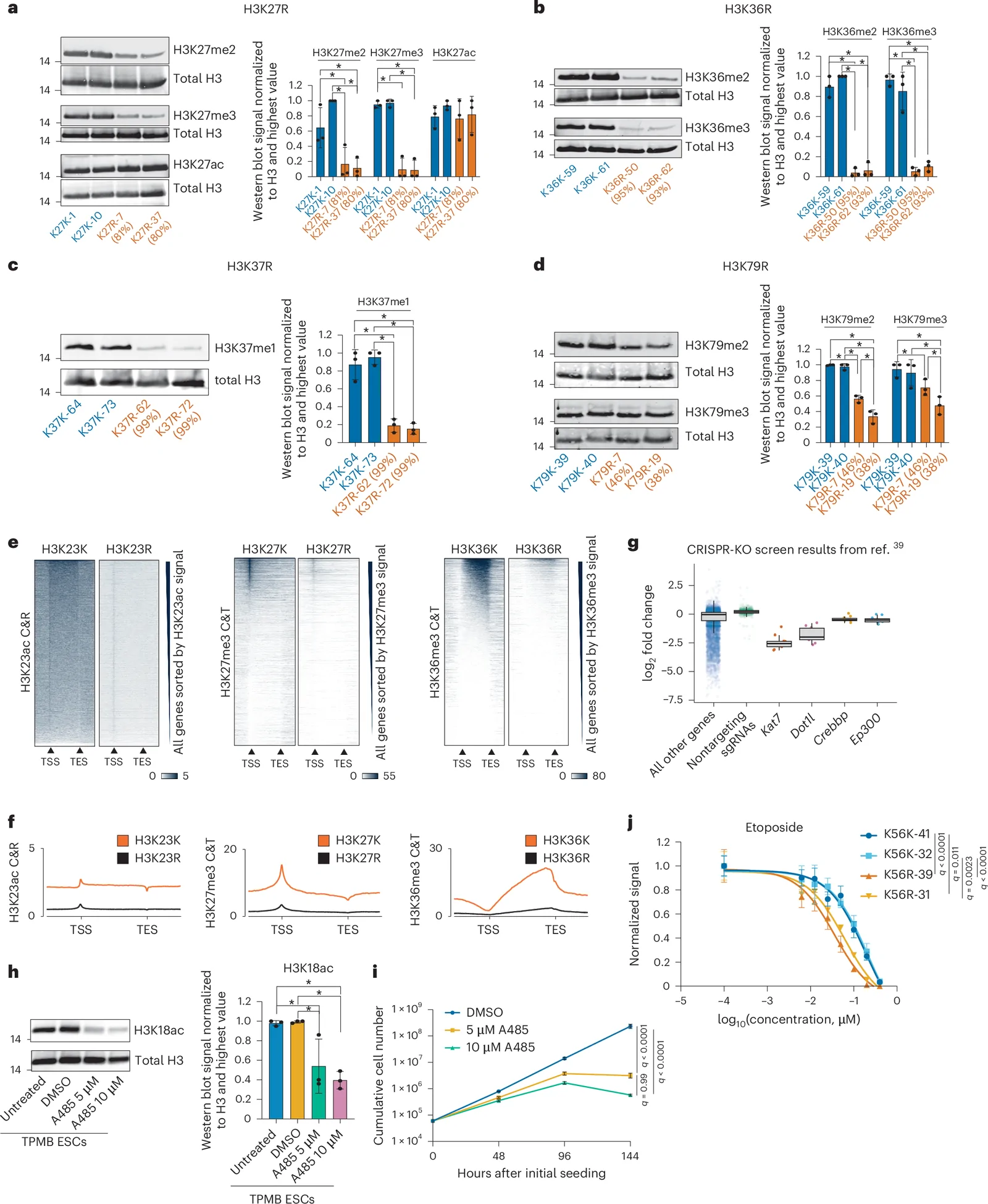
Histone mutagenesis induces loss of PTMs and functional effects. **a**, Western blot analysis of H3K27me2, H3K27me3 and H3K27ac levels in two H3K27K and two H3K27R clones. K-to-R editing efficiency estimated by NGS is shown in brackets. H3 levels are used as a loading control. Quantification is presented as mean ± 1 s.d. of *n* = 3 biological replicates (that is, independent blots from independent cell pellets). Statistical significance was assessed by two-way ANOVA followed by Tukey’s multiple comparisons test. *q < 0.05. Only significant comparisons are highlighted. **b**, Western blot analysis of H3K36me2 and H3K36me3 levels in two H3K36K and two H3K36R clones. K-to-R editing efficiency estimated by NGS is shown in brackets. H3 levels are used as a loading control. Quantification is presented as mean ± 1 s.d. of *n* = 3 biological replicates (that is, independent blots from independent cell pellets). Statistical significance was assessed by two-way ANOVA followed by Tukey’s multiple comparisons test. *q < 0.05. Only significant comparisons are highlighted. **c**, Western blot analysis of H3K37me1 levels in two H3K37K and two H3K37R clones. K-to-R editing efficiency estimated by NGS is shown in brackets. H3 levels are used as a loading control. Quantification is presented as mean ± 1 s.d. of *n* = 3 biological replicates (that is, independent blots from independent cell pellets). Statistical significance was assessed by one-way ANOVA followed by Tukey’s multiple comparisons test. *q < 0.05. Only significant comparisons are highlighted. **d**, Western blot analysis of H3K79me2 and H3K79me3 levels in two H3K79K and two H3K79R clones. K-to-R editing efficiency estimated by NGS is shown in brackets. H3 levels are used as a loading control. Quantification is presented as mean ± 1 s.d. of *n* = 3 biological replicates (that is, independent blots from independent cell pellets). Statistical significance was assessed by two-way ANOVA followed by Tukey’s multiple comparisons test. *q < 0.05. Only significant comparisons are highlighted. **e**, Heatmaps of C&R (H3K23ac) and C&T (H3K27me3 and H3K36me3) signals in H3K23K/H3K23R, H3K27K/H3K27R and H3K36K/H3K36R mutant cells, respectively. C&R data were normalized using *Drosophila* genome spike-in; C&T data were normalized using *Escherichia coli* genome spike-in. The experiment was performed in two biological replicates (that is, independent cell pellets). **f**, Average profiles of C&R (H3K23ac) and C&T (H3K27me3 and H3K36me3) signals in H3K23K/H3K23R, H3K27K/H3K27R and H3K36K/H3K36R mutant cells, respectively. C&R data were normalized using *Drosophila* genome spike-in; C&T data were normalized using *E. coli* genome spike-in. The experiment was performed in two biological replicates (that is, independent cell pellets). **g**, Box plot representing analysis of the CRISPR-KO screen results from ref. ^39^. log_2_ fold change is calculated as the difference in sgRNA abundance between day 0 and day 11 in the screen. Values for individual sgRNAs are shown as individual data points. Box plots show the median (horizontal line), interquartile range (box) and whiskers extending to 1.5× interquartile range. *n* = 2 biological replicates (that is, independently transduced cells). **h**, Western blot analysis of H3K18ac levels in TPMB cells treated with DMSO or A485 at 5 or 10 µM for 48 h. H3 levels are used as a loading control. Quantification is presented as mean ± 1 s.d. of *n* = 3 biological replicates (that is, independent blots from independent cell pellets). Statistical significance was assessed by one-way ANOVA followed by Tukey’s multiple comparisons test. *q < 0.05. Only significant comparisons are highlighted. **i**, Growth curve for TPMB cells treated with DMSO or A485 at 5 or 10 µM. Quantification is presented as mean ± 1 s.d. of *n* = 3 biological replicates (that is, independently seeded cells). Statistical significance was assessed by one-way ANOVA followed by Tukey’s multiple comparisons test (*q* < 0.05). **j**, Etoposide drug response curve for two H3K56K and two H3K56R clones. Quantification is presented as mean ± 1 s.d. of *n* = 3 biological replicates (that is, independently seeded cells). Differences in drug sensitivity across cell lines were assessed using one-way ANOVA with Sidak’s multiple comparisons correction (*q* < 0.05). Only significant comparisons are highlighted. TSS, transcription start site; TES, transcription end site. Source data

**Extended Data Fig. 6:**
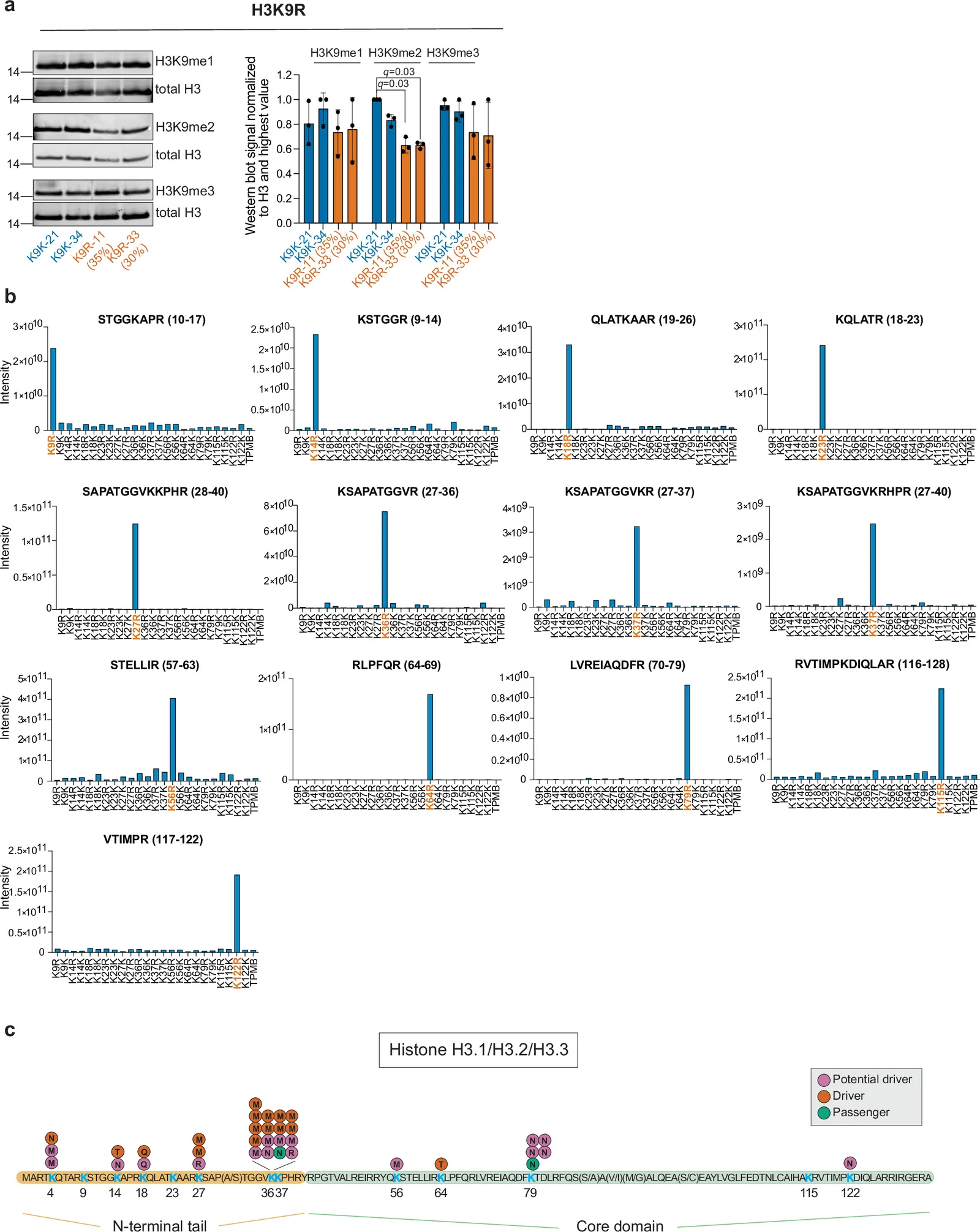
Histone mutagenesis induces loss of PTMs and functional effects. **a**, Western blot analysis of H3K9me1, H3K9me2 and H3K9me3 levels in two H3K9K and two H3K9R clones. K-to-R editing efficiency estimated by NGS is shown in brackets. H3 levels are used as a loading control. Quantification is presented as mean ± 1 s.d. of n = 3 biological replicates (that is, independent blots from independent cell pellets). Statistical significance was assessed by two-way ANOVA followed by Tukey’s multiple comparisons test (*q* < 0.05). Only significant comparisons are highlighted. **b**, Quantification of mutant-specific peptide sequences generated by K-to-R substitutions that introduce additional proteolytic cleavage sites. For the K64R, K79R and K115R substitutions, the peptides resulting from missed tryptic cleavage were used for quantitation due to their better detectability. H3K4R mutants were not analyzed due to low editing efficiency. **c**, Schematic of histone H3 lysine mutations identified across ~12,000 adult tumor samples. Driver classifications reported in ref. ^51^ are indicated by color, with amino acid substitutions shown in one-letter code. Source data

We then confirmed loss of histone PTMs in highly and fully edited clones. H3K27R clones showed loss of H3K27me2 and H3K27me3, with no change in H3K27ac signal (Fig. 7a), consistent with H3K27ac localization at both canonical and noncanonical H3 (refs. ^11,29^). H3K36R clones showed loss of H3K36me2 and H3K36me3 (Fig. 7b), while H3K37R clones displayed loss of H3K37me1 (Fig. 7c). Partially edited H3K9R clones (30–35%) showed no reduction in H3K9me1 or H3K9me3, and a modest decrease in H3K9me2 (Extended Data Fig. 6a). Partially edited H3K79R clones (39–45%) demonstrated reduced H3K79me2 and H3K79me3 levels (Fig. 7d). We could not reliably assess H3K56ac, H3K64ac, H3K115ac or H3K122ac because suitable antibodies could not be identified, a problem previously reported for H3K56ac^30,31^.

To assess genome-wide changes in histone modifications, we profiled H3K23ac, H3K27me3 and H3K36me3 in H3K23R, H3K27R and H3K36R mutants, respectively, using C&T or CUT&RUN (C&R). This confirmed global loss of the corresponding modifications in K-to-R mutants (Fig. 7e,f).

To confirm incorporation of mutant histones into chromatin, we performed histone acid extraction followed by MS^32,33^. Because free histones constitute <1% total histone pool in rapidly dividing cells^34^, this approach predominantly recovers chromatin-associated histones. Propionylation-based sample preparation blocks tryptic cleavage at lysines, restricting digestion to arginine residues^32,33^. K-to-R mutations, therefore, generate mutant-specific peptides, which were detected in all analyzed samples, confirming chromatin incorporation of mutant histones (Extended Data Fig. 6b).

Our screen identified H3K4, H3K9, H3K14, H3K18 and H3K79 as important for ESC fitness (Fig. 6). The H3K4R phenotype is consistent with ESC death after depletion of H3K4 methyltransferase complex components DPY30 and RBBP5 (ref. ^35^). Likewise, H3K9R phenotype aligns with the established role of H3K9 methylation in heterochromatin formation and the lethality observed after deletion of all six H3K9 methyltransferases^36^. H3K14 is primarily acetylated by KAT7 (ref. ^19^), while H3K79 undergoes dimethylation and trimethylation by DOT1L^37,38^. Consistent with the effects of H3K14R and H3K79R mutagenesis, single-guide RNAs (sgRNAs) targeting *Kat7* and *Dot1l* show strong depletion in the CRISPR-KO screen in ESCs^39^ (Fig. 7g). Although *Kat7* (ref. ^40^) and *Dot1l*^41^ KO ESCs can be derived, both exhibit markedly impaired proliferation. H3K18 is primarily acetylated by CBP and p300 (ref. ^20^). While individual *Cbp* or *Ep300* KOs are well-tolerated in ESCs (Fig. 7g), double-knockout ESCs have not been reported. To investigate whether loss of CBP/p300 catalytic activity affects ESC proliferation, we treated cells with CBP/p300 inhibitor A485 (ref. ^42^). This caused a marked reduction in H3K18ac levels (Fig. 7h) and cell death (Fig. 7i). Although CBP and p300 have multiple substrates^20^, our data support an important role for H3K18ac in mediating CBP/p300 function.

H3K56ac has been implicated in chromatin restoration following DNA replication and repair in yeast^43^, where H3K56R mutants exhibit heightened sensitivity to DNA damage^44–46^. Similarly, H3K56R causes developmental defects^9,47^ and genotoxic sensitivity in *Drosophila*^9^. To test whether this function is conserved in mammals, we treated fully edited H3K56R cells with etoposide. H3K56R mutants showed significantly increased etoposide sensitivity relative to H3K56K controls (Fig. 7j), demonstrating a conserved role for H3K56 in the regulation of genome stability.

Because histone mutations can act as oncogenic drivers^48–50^, we compared residues identified in our screen with cancer-associated histone mutations. A recent analysis of ~12,000 adult tumor samples^51^ identified driver mutations at H3K4, H3K14, H3K18, H3K27, H3K36 and H3K64 (Extended Data Fig. 6c). The frequency and driver status of these mutations did not correlate with fitness phenotypes observed in our screen. Notably, the oncogenic status of the acetyl-mimic H3K18Q mutation further supports the functional importance of H3K18ac.

### Combinatorial H3 lysine mutations reveal functional crosstalk

**Fig. 8:**
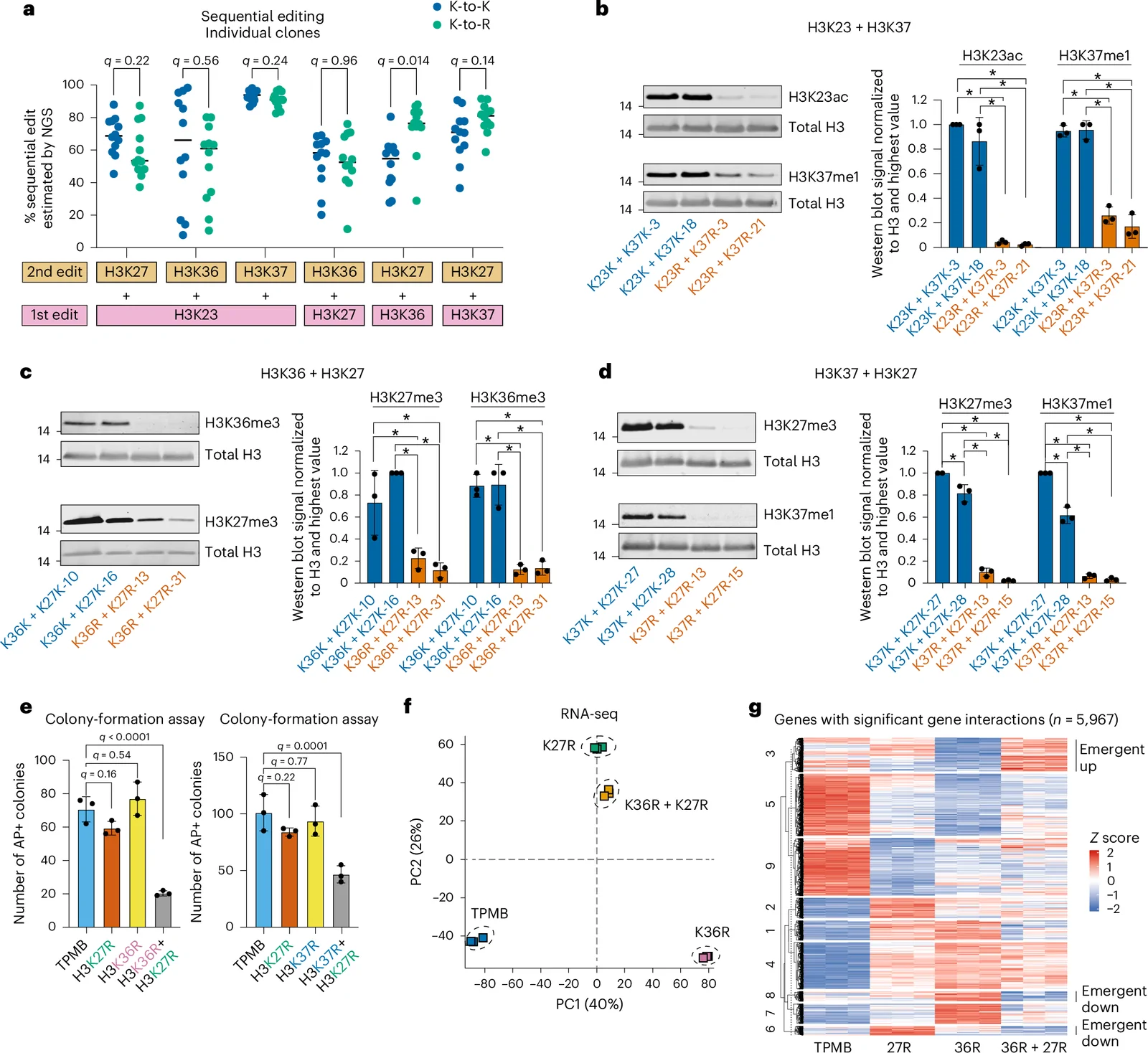
Combinatorial H3 lysine mutations reveal functional crosstalk. **a**, Scatterplots showing the percentage of reads containing the target (K-to-R or K-to-K) mutation in individual clonal cell lines across six sequential mutagenesis experiments. The genotypes of the starting cell lines are indicated in pink boxes, and the second edits introduced are indicated in brown boxes. The statistical significance was assessed using two-tailed unpaired *t* tests with Benjamini–Krieger–Yekutieli correction for multiple comparisons (*q* = 0.01). **b**, Western blot analysis of H3K23ac and H3K37me1 levels in two K23K + K37K and two K23R + K37R clones. H3 levels are used as a loading control. Quantification is presented as mean ± 1 s.d. of *n* = 3 biological replicates (that is, independent blots from independent cell pellets). Statistical significance was assessed by two-way ANOVA followed by Tukey’s multiple comparisons test. *q < 0.05. Only significant comparisons are highlighted. **c**, Western blot analysis of H3K27me3 and H3K36me3 levels in two K36K + K27K and two K36R + K27R clones. H3 levels are used as a loading control. Quantification is presented as mean ± 1 s.d. of *n* = 3 biological replicates (that is, independent blots from independent cell pellets). Statistical significance was assessed by two-way ANOVA followed by Tukey’s multiple comparisons test. *q < 0.05. Only significant comparisons are highlighted. **d**, Western blot analysis of H3K27me3 and H3K37me1 levels in two K37K + K27K and two K37R + K27R clones. H3 levels are used as a loading control. Quantification is presented as mean ± 1 s.d. of *n* = 3 biological replicates (that is, independent blots from independent cell pellets). Statistical significance was assessed by two-way ANOVA followed by Tukey’s multiple comparisons test. *q < 0.05. Only significant comparisons are highlighted. **e**, The number of AP-positive colonies recovered after plating WT (TPMB), single (H3K27R, H3K36R, H3K37R) or double (H3K36R + K3K27R, H3K37R + H3K27R) mutant cells. Quantification is presented as mean ± 1 s.d. of *n* = 3 biological replicates (that is, independently plated wells). Statistical significance was assessed by one-way ANOVA followed by Dunnett’s multiple comparisons test (*q* < 0.05). **f**, PCA of the RNA-seq samples, with samples colored by genotype. **g**, Heatmap of statistically significant gene interactions (*q* < 0.01) identified using DESeq2 with a factorial design to model genetic interactions between the two mutations (5,967 genes). Gene expression values were *z* scored and clustered using *k*-means clustering (*k* = 9). Gene clusters showing distinct expression patterns in the double mutant compared to single mutants and WT are indicated as emergent (clusters 3, 6 and 8). PCA, principal component analysis. Source data

**Extended Data Fig. 7:**
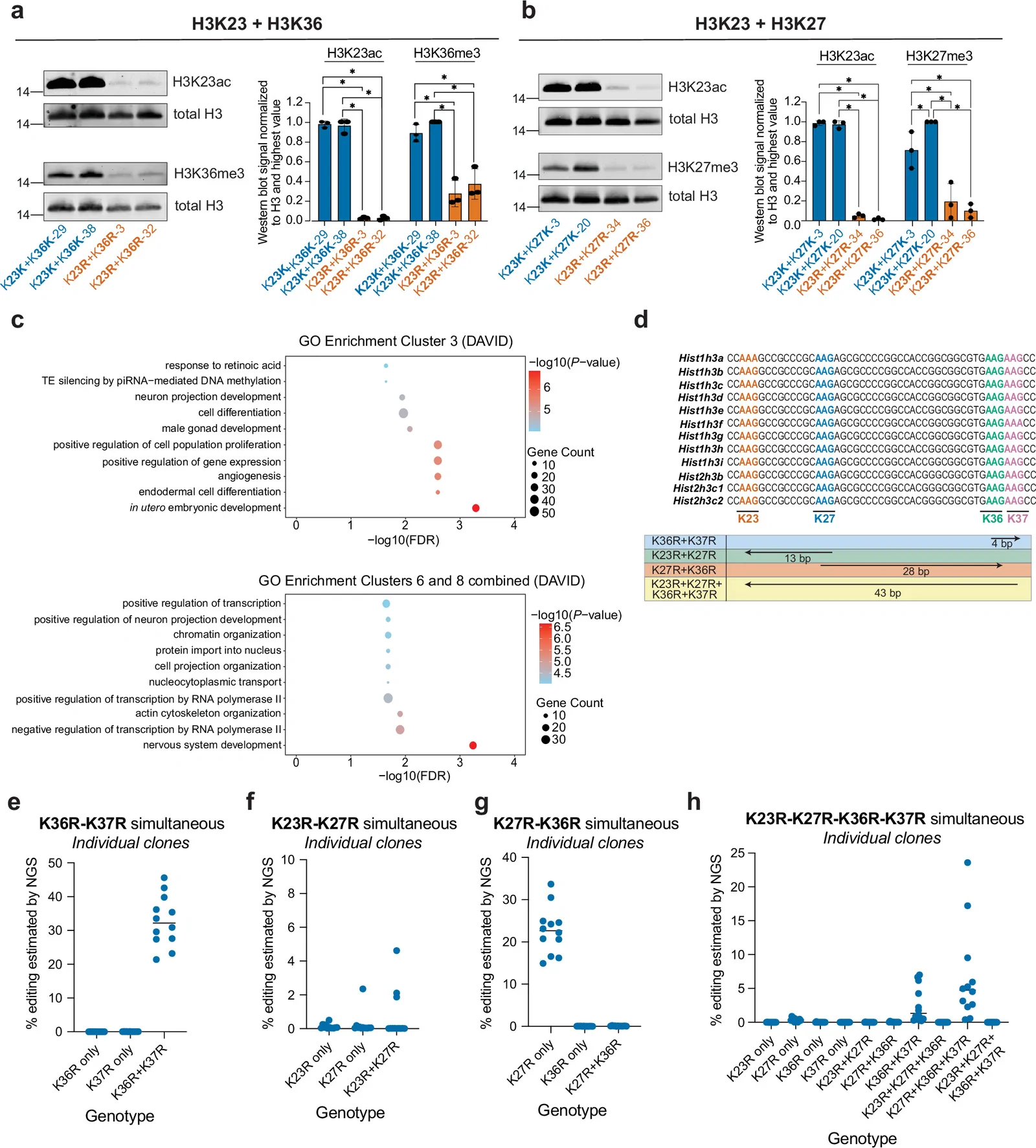
Combinatorial H3 lysine mutations reveal functional crosstalk. **a**, Western blot analysis of H3K23ac and H3K36me3 levels in two K23K + K36K and two K23R + K36R clones. H3 levels are used as a loading control. Quantification is presented as mean ± 1 s.d. of n = 3 biological replicates (that is, independent blots from independent cell pellets). **b**, Western blot analysis of H3K23ac and H3K27me3 levels in two K23K + K27K and two K23R + K27R clones. H3 levels are used as a loading control. Quantification is presented as mean ± 1 s.d. of n = 3 biological replicates (that is, independent blots from independent cell pellets). Statistical significance in **a** and **b** was assessed by two-way ANOVA followed by Tukey’s multiple comparisons test (* = *q* < 0.05). Only significant comparisons are highlighted. **c**, Gene Ontology (GO) analysis of genes from clusters 3, 6, and 8 in the heatmap shown in Fig. 8g, highlighting enriched biological processes. TE = transposable elements. **d**, Nucleotide sequence of *M. musculus* histone H3.1/2 genes spanning lysine 23 to lysine 37. Simultaneous targeting strategies are indicated below, with the longest distance between edited sites specified. **e**, Scatterplots showing the % of reads containing the K36R, K37R and K36R + K37R mutations in the individual cell clones after simultaneous K36R + K37R editing with single epegRNA (n = 12 for each group). **f**, Scatterplots showing the % of reads containing the K23R, K27R and K23R + K27R mutations in the individual cell clones after simultaneous K23R + K27R editing with single epegRNA (n = 12 for each group). **g**, Scatterplots showing the % of reads containing the K27R, K36R and K27R + K36R mutations in the individual cell clones after simultaneous K27R + K36R editing with single epegRNA (n = 12 for each group). **h**, Scatterplots showing the % of reads containing the target quadruple mutation (K23 + K27 + K36 + K37), as well as all possible single, double, and triple mutations, in individual cell clones following simultaneous quadruple editing with a single epegRNA (n = 12 for each group). Source data

To investigate functional interactions across lysine residues, we sequentially introduced double-mutant combinations involving H3K23R, H3K27R, H3K36R and H3K37R using highly edited single mutants as starting populations. Highly edited double-mutant clones were obtained for all attempted combinations (Fig. 8a) and showed loss of the corresponding histone modifications (Fig. 8b–d and Extended Data Fig. 7a,b). Editing efficiency during the second round of mutagenesis did not significantly differ between K-to-K and K-to-R conditions (Fig. 8a). For the H3K27R + H3K36R combination, comparable editing efficiencies were obtained irrespective of editing order. Notably, double H3K27R + H3K36R and H3K27R + H3K37R mutants showed reduced colony-forming capacity relative to the corresponding single mutants, indicating impaired stem cell self-renewal (Fig. 8e).

To further characterize the H3K27R + H3K36R phenotype, we performed RNA sequencing (RNA-seq) of WT, single-mutant and double-mutant cells (Fig. 8f). Differential expression analysis using a factorial DESeq2 (ref. ^52^) design was used to model genetic interactions between the two mutations (Fig. 8g). Among genes with significant interaction effects, most showed epistatic responses, with the double mutant resembling either the H3K27R (clusters 4, 5 and 7) or the H3K36R (clusters 2 and 9) single-mutant state. Smaller subsets showed buffering (cluster 1) or emergent (clusters 3, 6 and 8) transcriptional responses. Genes within the emergent groups were enriched for pathways linked to differentiation, proliferation and transcriptional regulation (Extended Data Fig. 7c), including downregulation of pluripotency-supporting factors *Klf4* (refs. ^53,54^), *Chd4* (ref. ^55^) and *Sin3a*^56^, and upregulation of differentiation drivers *Fgfr2* (ref. ^57^) and *Dkk*^58^, consistent with reduced stem self-renewal capacity of the double-mutant cells. Together, these findings demonstrate functional crosstalk between H3K27 and H3K36 modifications.

Next, we tested whether combinatorial H3K23R, H3K27R, H3K36R and H3K37R mutants could be generated using single epegRNAs encoding multiple substitutions (double and quadruple configurations; Extended Data Fig. 7d). Editing efficiency showed a strong inverse relationship with editing window size (Extended Data Fig. 7e–h). The shortest-range configuration (H3K36R + H3K37R) yielded the highest editing efficiency (up to 46%), whereas longer-range configurations produced predominantly partial or singly edited clones. Together, these findings demonstrate that simultaneous multisite histone mutagenesis with single epegRNAs is feasible but substantially less efficient than sequential editing.

## Discussion

In this study, we establish a prime editing platform for systematic mutagenesis of histone H3 lysines in mouse ESCs. This approach enables precise, multiplexed, reversible and variant-specific editing. Using comparative analysis of K-to-R and synonymous K-to-K substitutions, we defined the contribution of individual H3 lysines to ESC fitness and demonstrated corresponding loss of histone PTMs. Together, these findings establish a scalable framework for functional interrogation of histone PTMs in mammalian cells.

Transient mismatch repair inhibition using MLH1dn substantially enhanced prime editing efficiency, consistent with previous studies^15,16^. In contrast, nickRNA provided little additional benefit under mismatch repair inhibition conditions but increased the frequency of large-scale deletions at some loci. Under strong negative selection, prime editing led to the accumulation of partially edited PAM-only by-products resistant to further editing.

The strong fitness defects associated with H3K4R, H3K9R, H3K14R and H3K79R parallel developmental lethality phenotypes reported in *Drosophila*^9^. Interestingly, while H3K18R caused strong negative selection in ESCs, H3K18A flies are viable^9^, suggesting either species-specific requirements or distinct structural consequences of arginine substitution at this site.

Partially edited mutants represent useful systems for dissecting PTM function at fitness-critical residues. In H3K18R and H3K9R cells, we observed compensatory maintenance of H3K18ac and H3K9me1/H3K9me3, respectively, highlighting the importance of tightly regulated levels of these modifications for ESC fitness. In H3K18R mutants, compensation occurred through increased incorporation of nonmutated H3.3 at a subset of H3K18ac-marked loci, suggesting that specific chromatin regions can preserve key histone modifications through targeted histone exchange or remodeling. Consistent with context-dependent compensation across H3 variants, *Drosophila* histone mutagenesis studies showed that H3.1/H3.2 and H3.3 can compensate for one another at H3K9ac-marked loci^59^. However, variant-specific requirements emerge in repressive contexts—H3.1/H3.2K9 is required for repression of pericentromeric transposons, whereas H3.3K9 maintains telomeric heterochromatin^59^. Analyses of partially edited H3.1/H3.2K9R, H3.3K9R and combined H3.1/H3.2/H3.3K9R mutants in ESCs should help further define redundancy between variant and canonical PTMs at this site. Together, these findings highlight the value of variant-specific editing for resolving shared and specialized functions of histone PTMs.

Although multiple K-to-R H3 mutants were compatible with ESC proliferation, this does not imply their functional dispensability. In fact, *Drosophila* H3K23A, H3K56A, H3K64A and H3K115A mutants exhibit fertility defects^9^, while H3K27R/H3K27A, H3K36R and H3K37R mutations are developmentally lethal^8,9,47,60^. Consistent with this, *Nsd1*^*−/−*^ and *Setd2*^*−/−*^ ESCs exhibit severe differentiation defects^61,62^, and both knockouts are embryonic lethal in mice^63–65^. H3K27R likewise blocks ESC differentiation^11^, consistent with PRC2 loss-of-function phenotypes in mouse differentiation and development^66,67^. In addition, H3K56R mutations in yeast^44–46^, *Drosophila*^9^ and mouse ESCs display increased sensitivity to genotoxic stress, supporting an evolutionary conserved role for H3K56ac in regulating genome stability. Given the proposed roles of H3K64ac, H3K115ac and H3K122ac in nucleosome destabilization and transcriptional activation^68–71^, K-to-R mutants at these sites may exhibit context-dependent defects in transcription and chromatin accessibility. Consistent with this idea, H3K122R yeast mutants show delayed transcriptional responses upon thiamine withdrawal^71^, despite normal proliferation. In addition, enrichment of H3K64me3 at pericentromeric heterochromatin^72^ raises the possibility that H3K64R mutants may display altered heterochromatin organization. Future studies using histone mutants under specific developmental or stress conditions should help uncover context-dependent PTM functions at these sites.

Surprisingly, all tested double-mutant combinations were compatible with ESC proliferation under standard culture conditions. The H3K27R + H3K36R double mutant is particularly interesting given the well-established antagonism between H3K36me2/H3K36me3 and H3K27me3 (refs. ^61,73–80^). Most transcriptional interaction effects in this mutant were epistatic, although buffering and emergent responses were also observed. These findings are consistent with predominantly antagonistic interactions between H3K27 and H3K36 methylation, with one pathway dominating at many loci, while also suggesting additional layers of chromatin crosstalk.

Several caveats should be considered when interpreting histone K-to-R mutations. These substitutions abolish all PTMs at a given lysine residue, making it difficult to attribute phenotypes to individual modifications. In addition, mutations within the histone fold domain may affect histone–DNA or histone–histone interactions independently of PTM loss. Histone mutagenesis is therefore most informative at residues dominated by a single major modification and should ideally be interpreted alongside perturbations of the corresponding chromatin-modifying enzymes.

Finally, this methodology extends beyond histone H3 PTM analysis. The same strategy should apply to other canonical and variant histones, oncohistones^48–50^ and repetitive genomic elements such as ubiquitin, rRNA and tRNA genes or transposable elements that remain challenging to manipulate using conventional genome engineering approaches.

## Methods

No ethical issues are associated with this study.

### Plasmids and cloning

For epegRNA cloning, the pL-CRISPR-SFFV-Puro-P2A-EGFP_peg plasmid was generated by removing the sgRNA scaffold from pL-U6-sgRNA-SFFV-Puro-P2A-EGFP (Addgene, 175037) and by adding two thymidine nucleotides to the RNA Pol III terminator for increased termination efficiency (Addgene, 253729). For nickRNA cloning, pLKO5.sgRNA.EFS.tRFP657 (Addgene, 57824) was used.

PEmax-2A-BFP, PEmax-2A-MLH1dn or PEmax-2A-MLH1dn-2A-BFP were cloned into either pPB-CAG-Dest-pA-pgk-hygro or pPB-TetO-Dest-pA-pgk-hygro piggyBac vectors (provided by J. Silva, Guangzhou Laboratory), using pCMV-PEmax-P2A-hMLH1dn (Addgene, 174828) as a PCR template. pPB-rtTA-IRES-zeo was also provided by J. Silva. PB-TetO-PEmax-2A-MLH1dn-2A-BFP was deposited to Addgene (253728).

For epegRNA cloning, forward and reverse oligos encoding the spacer, extension, scaffold and stability^14^ elements were phosphorylated and annealed using T4 DNA ligase buffer (NEB, B0202) and T4 PNK (NEB, M0201). For nickRNAs, only spacer oligos were annealed. Annealed oligos were then added to either pL-U6-pegRNA-SFFV-Puro-P2A-EGFP or pLKO5.sgRNA.EFS.tRFP657 and subjected to sequential digestion by BsmBI-v2 (NEB, R0379) and ligation with T4 DNA ligase (NEB, M0202). Constructs were transformed into Stbl3 competent cells.

### EpegRNA and nickRNA design

For H3K14R, H3K18R and H3K23R mutagenesis, epegRNAs and nickRNAs were designed using PrimeDesign (v.0.2)^81^, while epegRNAs for all other H3 lysines were designed using DeepPrime (v.3.6)^82^. K-to-K epegRNAs were identical to their K-to-R counterparts, except for the target mutation in the extension sequence, which was changed manually. All the epegRNA and nickRNA sequences are listed in Supplementary Table 1.

### Cell culture

Mouse ESCs (E14Tg2a line; gift from J. Brickman, University of Copenhagen) were cultured in Glasgow’s Minimum Essential Medium (Sigma-Aldrich, G5154) supplemented with penicillin–streptomycin, L-glutamine (Gibco, 25030-081), MEM NEAA (Gibco, 11140-035), sodium pyruvate (Gibco, 11360-039), β-mercaptoethanol (Gibco, 21985023), 10% FBS and leukemia inhibitory factor (produced in-house). Cells were maintained at 37 °C with 5% CO_2_ and regularly tested for mycoplasma.

The TPMB ESC line was generated by cotransfecting WT ESCs with pPB-rtTA-zeo, pPB-TetO-2A-MLH1dn-2A-BFP-hygro and PBase transposase plasmids, followed by selection with zeocin and hygromycin B.

For transfection, epegRNAs and nickRNAs were delivered using Lipofectamine 3000 (Invitrogen, L3000008) in reduced-serum medium (Gibco, 31985062) according to the manufacturer’s instructions. TPMB ESCs were cultured with doxycycline (1 µg ml^−1^) throughout mutagenesis. Twenty-four hours post-transfection, cells were selected with puromycin.

For lentiviral transduction, lentivirus was produced by transfecting HEK293FT cells using a standard calcium phosphate method^83^. ESCs were transduced with viral supernatant in the presence of polybrene (10 µg ml^−1^) for 24 h.

### Flow cytometry

Flow cytometry was performed using a Sony MA900 cell sorter. Cells edited in the presence of nickRNA were sorted based on BFP, eGFP and RFP657, whereas cells edited without nickRNA were sorted based on BFP and eGFP.

### Amplicon-based NGS and data analysis

Cell lysis was performed by adding lysis buffer (10 mM Tris–HCl, pH 7.5, 0.05% SDS, 50 µg ml^−1^ proteinase K) and incubating at 65 °C for 10 min, then 95 °C for 5 min. Amplicon libraries were prepared on an Opentrons Flex robot. Briefly, genomic DNA was used in two sequential PCR reactions, using the PCR1 product as input for PCR2. PCR1 primers amplified the target genomic region and introduced partial Illumina adaptors, while PCR2 primers added unique sample barcodes. Primer sequences are listed in Supplementary Table 1. One PCR1 primer pair was used to genotype H3.1 alleles for all sites except H3K4 and H3K122. For these residues, each H3.1 allele was genotyped individually using gene-specific primers due to sequence divergence. Both PCR steps used Phusion High-Fidelity PCR Master Mix (Thermo Fisher Scientific, F531) with cycling conditions of 98 °C for 30 s, 28 cycles for PCR1 or 10 cycles for PCR2 of 98 °C for 10 s, 65 °C for 20 s and 72 °C for 15 s (PCR1) or 20 s (PCR2), followed by 72 °C for 5 min. PCR products were purified using Mag-Bind TotalPure NGS beads (Omega Bio-Tek, M1378; 0.6× ratio) and quantified using the Qubit 1× dsDNA High Sensitivity kit (Invitrogen, Q33231) and a High-Sensitivity D1000 ScreenTape (Agilent Technologies, 5067-5584) on an Agilent Tapestation 4150, before equimolar pooling. Sequencing was performed using Illumina MiSeq (Micro or Nano (v.2), 300 cycles; Nano Kit (v.2), 500 cycles), or NextSeq 1000/2000 (P2 XLEAP-SBS, 300 cycles).

Editing outcomes were analyzed using CRISPResso2 (v.2.3.4)^84^. Editing efficiency was defined as the percentage of reads containing the desired base change. PAM-only mutation rates were calculated by subtracting the desired edit percentage from the PAM mutation percentage. Indel frequency was determined as the proportion of reads containing insertions or deletions within an 80-bp window surrounding the cut site. Only clones with indel rates below 2% were included in subsequent experiments, as higher rates were considered indicative of true genomic alterations rather than PCR or sequencing errors. Because the amplicons encompass 18 histone H3 alleles, a true indel in a single allele would be expected to appear in ~5% reads for that clone.

### qPCR for assessing editing efficiency

Clones from all the editing experiments were prescreened by qPCR to estimate the ratio of edited-to-WT H3 alleles before NGS analysis. Primers were designed as described in ref. ^18^. Reactions were prepared using PowerUp SYBR green (Thermo Fisher Scientific, 100029284) and analyzed on QuantStudio 7 Pro instrument.

### qPCR for assessing histone cluster 1 copy number

Histone cluster 1 copy number assessment was performed using TaqMan probes against *Trim38* (Thermo Fisher Scientific, Mm00608206_cn), located within the histone cluster 1, and TaqMan copy number reference assay *Tfrc* (Thermo Fisher Scientific, 4458366). Reaction mix was prepared using TaqMan Genotyping master mix (Thermo Fisher Scientific, 4371353) following the manufacturer’s instructions and analyzed using QuantStudio 7 Pro instrument and CopyCaller (v.2.1) software (Thermo Fisher Scientific). Copy number estimates were subsequently categorized as 1 (values < 1.5), 2 (values ≥ 1.5 and <2.5) or 3 (≥2.5) copies.

### H3.3 genotyping

Direct genotyping of *H3f3a* from genomic DNA was hindered by the presence of numerous (>100) pseudogenes, consistent with reports for the human ortholog *H3F3A*^85^. Instead, we performed genotyping using cDNA. *H3f3b* was genotyped using genomic DNA. Primer sequences are listed in Supplementary Table 1.

### Western blotting

Cell pellets (~3 × 10^6^ cells) were collected and lysed by the addition of eight units of Benzonase Nuclease (Merck Millipore, 70746) and 300 µl LBS lysis buffer (50 mM Tris, pH 8.5, 150 mM NaCl, 2% SDS, 1% SDC, 2% IGEPAL CA-630, supplemented with protease inhibitors). Lysates were incubated at room temperature for 5 min, followed by 70 °C for 10 min. Protein concentrations were measured using the Pierce BCA assay (Thermo Fisher Scientific, 23227). Equal amounts of protein were resolved on Bolt Bis–Tris 4–12% gradient gels (Invitrogen, NW0412) in 1× Bolt MES-SDS running buffer (Invitrogen, B0002). Proteins were transferred onto nitrocellulose (Invitrogen, IB23001) or PVDF (Invitrogen, IB24001) membranes, using the iBlot 2 System (Invitrogen). Membranes were blocked for 1 h at room temperature in 5% milk in PBS-T (0.1% Tween-20), followed by overnight incubation with primary antibodies at 4 °C. After washing four times in PBS-T, membranes were incubated with secondary antibodies for 1 h at room temperature, then washed four additional times and imaged using the Odyssey M scanner (Li-Cor Biosciences). For each blot, two primary antibodies raised in different species were used—one targeting the histone modification of interest and one targeting the loading control (total H3). Species-specific secondary antibodies conjugated to distinct fluorophores (700 nm and 800 nm) were imaged in separate channels. Depending on the species of the primary antibody used to detect the target modification (mouse or rabbit), a total H3 antibody raised in the opposite species was used as the loading control to enable simultaneous dual-channel detection without cross-reactivity. Antibody specificity was ensured by our SNAP–ChIP data, prior modification-specific validation reported in the literature or by vendors, and independent benchmarking available at https://chromatinantibodies.com. All the antibody information is provided in Supplementary Table 2. For quantification, infrared fluorescence intensities were measured using Image Studio software (v.5.2; Li-Cor Biosciences). Rectangular regions of interest were applied across the bands, and local background subtraction was performed using the software’s standard median background method. Signal intensity for each target histone modification was normalized to the corresponding total H3 signal from the same lane and used for statistical analysis. All western blot experiments were independently repeated at least thrice using independently collected cell pellets.

### Shallow whole-genome sequencing and data analysis

Genomic DNA was extracted from ~1 × 10^6^ cells using the Monarch Genomic DNA purification kit (NEB, T3010). Next, 1,000 ng of genomic DNA was fragmented using a Covaris E220 ultrasonicator (Covaris, 500239) to a target size of 200 bp. Sequencing libraries were prepared using the NEBNext Ultra II DNA Library Prep Kit for Illumina (NEB, E7645). Final libraries were assessed using a high-sensitivity D1000 ScreenTape on an Agilent Tapestation 4150, pooled at equimolar concentrations and sequenced on the NextSeq 1000/2000.

Adaptor sequences were trimmed using Cutadapt (v.4.9)^86^. Reads were aligned to the mm10 genome using Bowtie 2 (v.2.5.4)^87^ with parameters: --end-to-end --very-sensitive --no-mixed --no-discordant --dovetail. SAM files were converted to BAM format with SAMtools (v.1.21) view, sorted and indexed with SAMtools sort and SAMtools index^88^. BAM files were converted to BigWig format using deepTools (bamCoverage; v.3.5.5)^89^ and visualized in IGV (v.2.19.7)^90^.

Copy number analysis was performed in R using the ACE package (v1.30.0)^91^. BAM files were processed with runACE (genome = mm10, binsize = 100 kb), and ploidy and cellularity estimations were calculated using loopsquaremodel with default parameters (penalty = 0.8, penploidy = 1).

### Embryoid body differentiation

To induce embryoid body differentiation, 1.5 million ESCs were plated in low-adherent petri dishes in the serum-containing media without leukemia inhibitory factor and cultured for 11 days with media change every 2 days.

### Gastruloid formation

Gastruloids were cultured as previously described^17^. Briefly, 300 ESCs were aggregated in 40 μl N2B27 media (50% DMEM-F12 (Gibco, 11320074), GlutaMAX (Thermo Fisher Scientific, 35050038), 50% Neurobasal (Thermo Fisher Scientific, 21103049), 1× B27 (Gibco, 175040044), 1× N2 (Cambridge Stem Cell Institute), 50 μM β-mercaptoethanol (Gibco, 31350010)) in 96-well U-bottom plates (Greiner Cellstar, 650185). After 48 h, 150 μl of 3 μM Chiron in N2B27 was added per well. At 72 and 96 h, 150 μl of medium was removed and substituted with 150 μl of fresh N2B27. Gastruloids were imaged on EVOS M5000 (Thermo Fisher Scientific).

### Proliferation assay

Cells were plated at 100,000 cells per well (six-well plate), with three replicates per condition in 3 ml ESC media, as well as treatment. Every 48 h, cells were detached and counted using Countess 3 FL Automated Cell Counter (Thermo Fisher Scientific) and reseeded at 100,000 cells per well. This process was repeated for 144 h after initial plating.

### Colony-formation (self-renewal) assay

Cells were plated at 200 cells per well (12-well plate), with three replicates per condition in 1.5 ml ESC media. The media was changed every 48 hrs, and the colonies were stained after 6 days of culture using Leukocyte Alkaline Phosphatase Kit (Sigma-Aldrich, 86R).

### SNAP–ChIP

SNAP–ChIP was performed using a standard ChIP–seq protocol described in ref. ^83^ with the following modifications: before antibody addition, 2 μl of KAcyl-Stat panel (EpiCypher, 19-3001) per 10 μg of chromatin were added to the reaction. The reads were mapped to the barcode panel with Bowtie 2 (v.2.5.4)^87^.

### RNA extraction, cDNA synthesis and qPCR gene expression analysis

RNA extraction was performed using the RNeasy Mini Kit (Qiagen, 74016). Next, 1,000 ng of RNA was used to perform reverse transcription with Transcriptor Universal cDNA Master (Roche, 5893151001). qPCR was then performed in technical triplicate using 5 ng of cDNA and PowerUp SYBR Green Master Mix (Thermo Fisher Scientific, 100029284) and analyzed on QuantStudio 7 Pro instrument. Levels of gene expression were measured by relative quantification to a housekeeping gene (*Gapdh*). Primers are listed in Supplementary Table 1.

### Automated C&R

H3K23ac C&R assay was automated through the Opentrons Flex platform equipped with a thermocycler module, plate heater module and magnetic block. The protocol was created in Opentrons Protocol Designer (v8.3.0; https://designer.opentrons.com/) based on C&R steps previously described^92^. Before starting the C&R protocol, 10% *Drosophila* S2 cells were added to the sample. The cells were fixed with 0.1% formaldehyde for 1 min, and fixation was quenched by adding glycine to 125 mM. Nuclei were isolated with nuclear extraction buffer (20 mM HEPES–KOH (pH 7.9), 10 mM KCl, 0.1% Triton X-100 and 20% (vol/vol) glycerol) and bound to ConA Paramagnetic Beads (EpiCypher, 21-1401). After the elution step, DNA was de-crosslinked overnight at 55 °C and purified using Mag-Bind TotalPure NGS beads (Omega Bio-Tek, M1378-01). Libraries were prepared using the NEBNext Ultra II DNA Library Prep Kit for Illumina (NEB, E7645S) and validated for quality and size distribution using an Agilent Tapestation 4150. Sequencing was performed using the NextSeq 1000/2000. The antibody is listed in Supplementary Table 2.

### Automated C&T

C&T assays were automated through the Opentrons Flex platform equipped with a thermocycler module, plate heater module and magnetic block. The protocol was created in Opentrons Protocol Designer software based on the C&T steps previously described^93^. Libraries were amplified through CUTANA nonhot start PCR master mix (EpiCypher, 15-1018) with 16–17 cycles. Resulting libraries were validated for quality and size distribution using an Agilent Tapestation 4150 and sequenced using the NextSeq 1000/2000. All antibodies are listed in Supplementary Table 2.

### C&T and C&R analysis

Adaptor sequences were removed with Cutadapt (v4.9)^86^. Trimmed reads were aligned to the mm10 or spike-in (dm6/eschColi_K12) genomes (as indicated in the legends to Fig. 7e,f) using Bowtie 2 (v.2.5.4)^87^ with parameters --end-to-end --very-sensitive --no-mixed --no-discordant --dovetail -p 16 --phred33 -I 10 -X 700 -x. Resulting alignments were processed using SAMtools (v.1.21) view, sort and index^88^. Spike-in scale factors were calculated as the percentage of reads aligned to the spike-in genome in the reference relative to the sample. BigWig files were generated using deepTools (bamCoverage; v.3.5.5 (ref. ^89^); -p max --effectiveGenomeSize 2652783500 --normalizeUsing RPGC -bs 5 --smoothLength 100) and subsequently scaled by sample-specific spike-in factors. Tracks were visualized in IGV (v.2.19.7)^90^. BED files were generated with BEDTools (v.2.31.1)^94^ bamtobed, and replicate BED files were merged and analyzed in EaSeq (v.1.2)^95^. H3K18ac peaks were called using MACS3 (v.3.0.3 (ref. ^96^); -f BEDPE --max-gap 300 -q 0.005 –broad) for each of three replicates. A consensus peak set was defined as peaks present in at least two of three replicates.

### RNA-seq and analysis

RNA extraction was performed using the RNeasy Mini Kit (Qiagen, 74016) and quality checked using an RNA ScreenTape (Agilent Technologies, 5067-5576) on an Agilent Tapestation 4150. RNA-seq libraries were prepared using the poly(A) mRNA magnetic isolation module (New England Biolabs, E7490) and Ultra II directional RNA library prep for Illumina kits (New England Biolabs, E7760), using 1 µg total RNA input from three independent biological replicates per sample. Libraries were quantified using the Qubit 1× dsDNA High Sensitivity kit (Invitrogen, Q33231) and a high-sensitivity D1000 ScreenTape (Agilent Technologies, 5067-5584) on an Agilent Tapestation 4150 and pooled at equimolar concentrations. The libraries were sequenced using the NextSeq 1000/2000.

Raw reads were trimmed using Cutadapt (v.4.9)^86^ and mapped using HiSat2 (v.2.2.1) to the mm10 genome assembly with standard paired-end alignment settings^97^. Raw gene count data were used as input for analysis using DESeq2 (v.1.52.0)^52^, with genes with fewer than ten total counts across all samples excluded before processing. Genotype effects were modeled using a factorial design including mutation A, mutation B and their interaction (~mutA + mutB + mutA:mutB) to identify nonadditive effects in the double mutant. log_2_ fold changes and adjusted *P* values were calculated using DESeq2, with multiple-testing correction performed using the Benjamini–Hochberg method. Genes with false discovery rate-adjusted interaction *P* values <0.01 were *z*-score normalized and clustered using *k*-means clustering (*k* = 9).

Gene Ontology functional analysis was performed using the Database for Annotation, Visualization, and Integrated Discovery (2025 release)^98,99^.

### Histone MS analysis

ESC pellets containing ~3 × 10^6^ cells were resuspended in ice-cold 50 µl of 0.2 M H_2_SO_4_, sonified with a Bioruptor at 4 °C for ten cycles of 30 s and incubated overnight at 4 °C with rotation to ensure complete histone extraction. After centrifugation at 14,000*g* and 4 °C for 30 min, 25 μl of the supernatant was neutralized with the same volume of 2 M NH_4_OH. Proteins were precipitated on magnetic beads with 100% EtOH, washed with 80% EtOH and lysine residues were propionylated with 20 µl of 200 mM NHS-propionate in ACN for 30 min at 50 °C. Samples were digested at 37 °C overnight with trypsin (Sequencing Grade Modified Trypsin, Promega GmbH). Tryptic peptides were desalted, purified using C18 Stagetips, vacuum dried and resuspended in 20 μl of 0.3% TFA and 2% ACN for liquid chromatography-tandem mass spectrometry (LC–MS/MS) measurement.

For LC–MS/MS, the peptides were injected into an Ultimate 3000 RSLCnano system and separated using a 25-cm analytical column (75 µm ID, 1.6 µm C18, Aurora-IonOpticks), with a 50-min gradient from 2% to 35% acetonitrile in 0.1% formic acid. The effluent from the HPLC was directly electrosprayed into an Orbitrap Exploris 480 instrument operating in data-dependent mode, which automatically transitions between full-scan MS and MS/MS acquisition. Typical mass spectrometric conditions were as follows: spray voltage, 1.5 kV; no sheath and auxiliary gas flow; heated capillary temperature, 275 °C; ion selection threshold, 5 × 10^3^ counts; dynamic exclusion, 20 s. Survey full-scan MS spectra (*m*/*z* 250–1200) were acquired with resolution of 60,000 at *m*/*z* 400 (max IT 40 ms, AGC target of 3 × 10^6^). The 15 most intense peptide ions with charge states between 2 and 6 were sequentially isolated to a target value of 2 × 10^5^, fragmented at 30% normalized collision energy and acquired with resolution of 15,000 at *m*/*z* 400 (max IT 40 ms).

Extracted ion chromatograms of doubly and triply charged peptide masses were quantified with the Skyline (v.25.1). Automatic selection of peaks was manually curated based on the relative retention times and fragmentation spectra with results from Proteome Discoverer (v.2.2). Integrated peak values were exported to CSV format for further calculations.

### Statistics and reproducibility

Information on statistical analyses and the number and nature of biological replicates for each experiment is provided in Supplementary Table 3 and the corresponding figure legends. Normality was assumed for *t*‑test‑based and analysis of variance‑based analyses, but was not formally tested. No statistical method was used to predetermine sample size. The experiments were not randomized. The investigators were not blinded to allocation during experiments and outcome assessment. For amplicon-based NGS experiments, samples with fewer than 1,000 reads were excluded from the analysis.

### Reporting summary

Further information on research design is available in the Nature Portfolio Reporting Summary linked to this article.

## Online content

Any methods, additional references, Nature Portfolio reporting summaries, source data, extended data, supplementary information, acknowledgements, peer review information; details of author contributions and competing interests; and statements of data and code availability are available at https://doi.org/10.1038/s41588-026-02675-y.

## Supplementary information

Reporting Summary

Peer Review file

Supplementary Tables Supplementary Tables 1–3.

## Source data

Source Data Fig. 1 Numerical source data.

Source Data Fig. 2 Numerical source data.

Source Data Fig. 3 Numerical source data.

Source Data Fig. 4 Numerical source data.

Source Data Fig. 5 Numerical source data.

Source Data Fig. 6 Numerical source data.

Source Data Fig. 7 Numerical source data.

Source Data Fig. 8 Numerical source data.

Source Data Fig. 1 Unprocessed western blots.

Source Data Fig. 2 Unprocessed western blots.

Source Data Fig. 3 Unprocessed western blots.

Source Data Fig. 4 Unprocessed western blots.

Source Data Fig. 7 Unprocessed western blots.

Source Data Fig. 8 Unprocessed western blots.

Source Data Extended Data Fig. 1 Numerical source data.

Source Data Extended Data Fig. 2 Numerical source data.

Source Data Extended Data Fig. 3 Numerical source data.

Source Data Extended Data Fig. 4 Numerical source data.

Source Data Extended Data Fig. 5 Numerical source data.

Source Data Extended Data Fig. 6 Numerical source data.

Source Data Extended Data Fig. 7 Numerical source data.

Source Data Extended Data Fig. 5 Unprocessed western blots.

Source Data Extended Data Fig. 6 Unprocessed western blots.

Source Data Extended Data Fig. 7 Unprocessed western blots.

## Data Availability

The amplicon-based and whole-genome NGS data were submitted to SRA (PRJNA1268213). The C&T, C&R and RNA-seq data were submitted to the GEO (C&T—GSE319420, C&R—GSE319506, RNA-seq—GSE319059). Source data are provided with this paper.

## Code availability

No custom code was created in this study.
