# Supplementary material for: Identifying critical lysines in mammalian histone H3 with high-throughput CRISPR prime editing
Source: Nat Genet. 2026 Jul 8;58(9):2303–19. doi: 10.1038/s41588-026-02675-y (PMC13553292; doi:10.1038/s41588-026-02675-y)
Supplement: Supplementary file 1 [file 41588_2026_2675_MOESM11_ESM.pdf]

Fig.1j

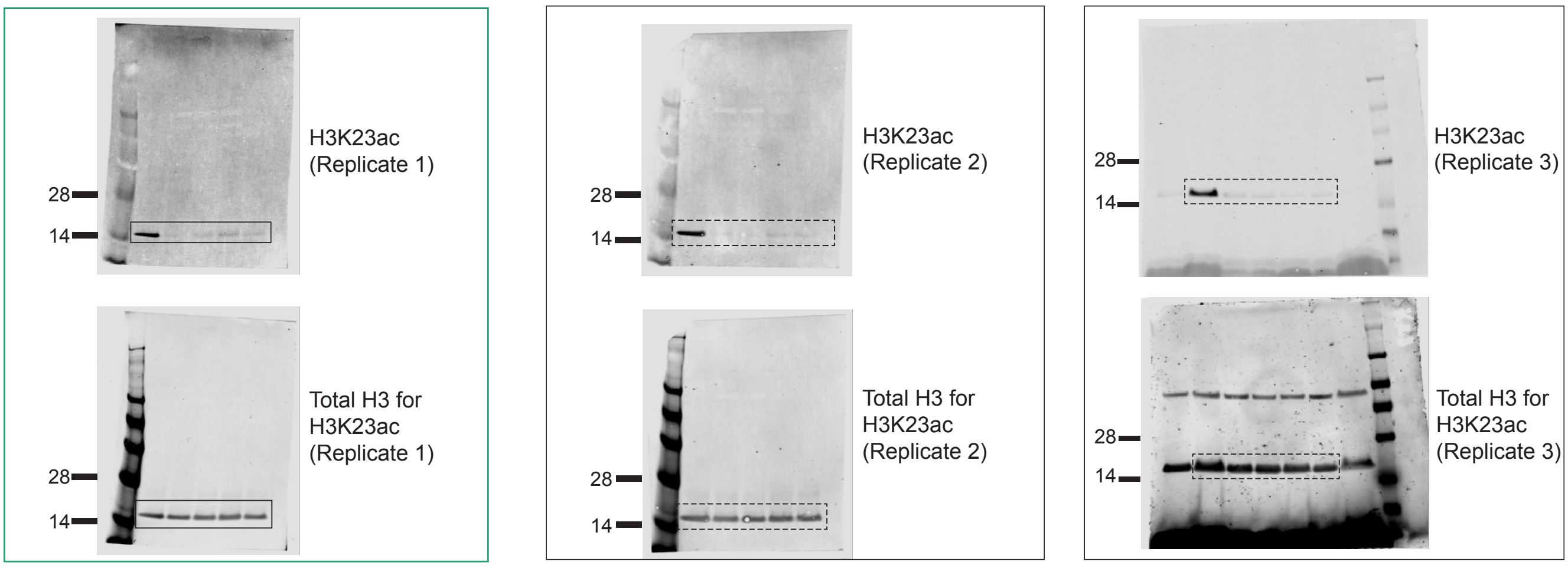

28
14
H3K23ac
(Replicate 1)
28
14
Total H3 for
H3K23ac
(Replicate 1)
28
14
H3K23ac
(Replicate 2)
28
14
Total H3 for
H3K23ac
(Replicate 2)
28
14
H3K23ac
(Replicate 3)
28
14
Total H3 for
H3K23ac
(Replicate 3)
