# Supplementary material for: Identifying critical lysines in mammalian histone H3 with high-throughput CRISPR prime editing
Source: Nat Genet. 2026 Jul 8;58(9):2303–19. doi: 10.1038/s41588-026-02675-y (PMC13553292; doi:10.1038/s41588-026-02675-y)

Fig.7a

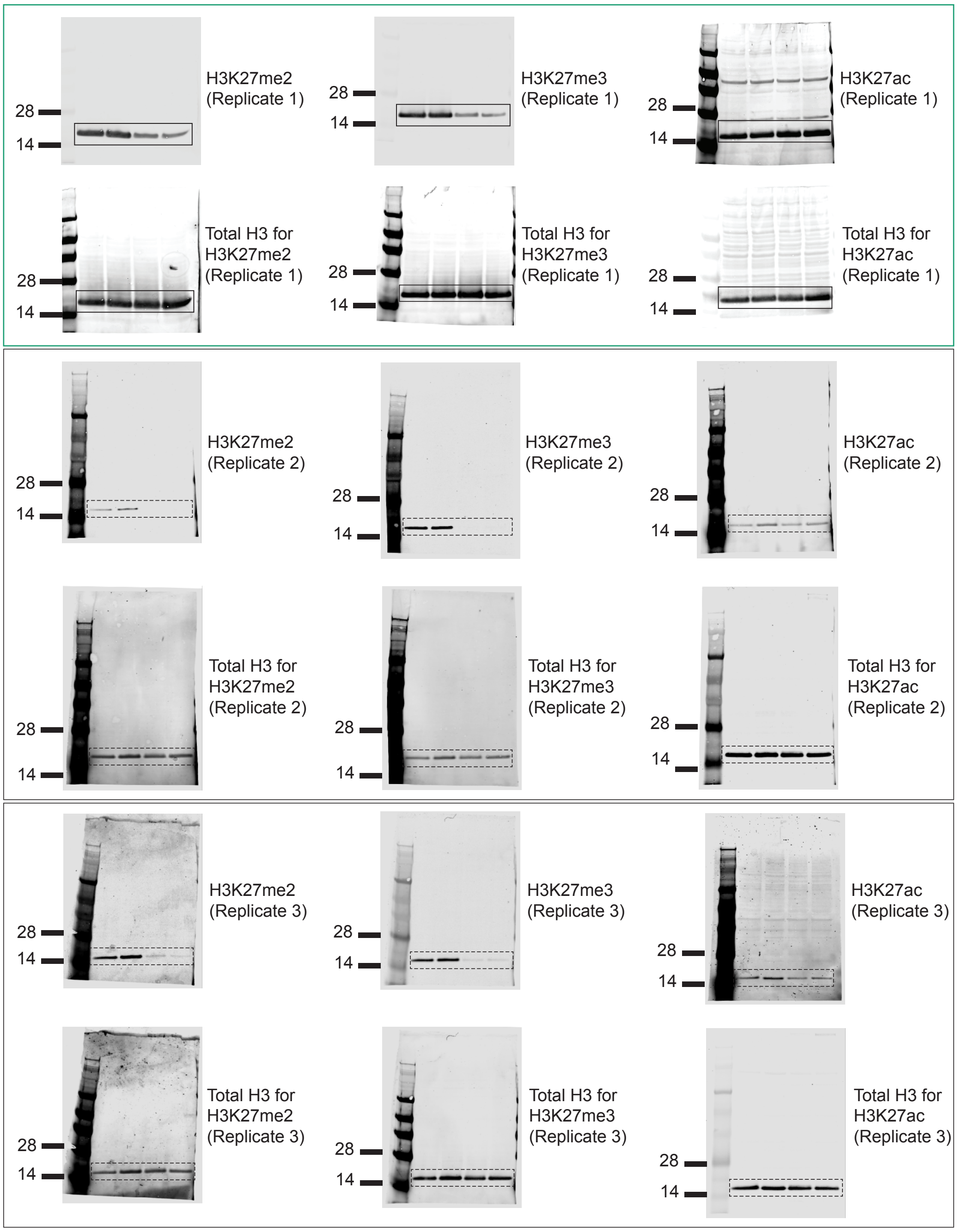
H3K27me2
(Replicate 1)
H3K27me3
(Replicate 1)
H3K27ac
(Replicate 1)
Total H3 for
H3K27me2
(Replicate 1)
Total H3 for
H3K27me3
(Replicate 1)
Total H3 for
H3K27ac
(Replicate 1)
H3K27me2
(Replicate 2)
H3K27me3
(Replicate 2)
H3K27ac
(Replicate 2)
Total H3 for
H3K27me2
(Replicate 2)
Total H3 for
H3K27me3
(Replicate 2)
Total H3 for
H3K27ac
(Replicate 2)
H3K27me2
(Replicate 3)
H3K27me3
(Replicate 3)
H3K27ac
(Replicate 3)
Total H3 for
H3K27me2
(Replicate 3)
Total H3 for
H3K27me3
(Replicate 3)
Total H3 for
H3K27ac
(Replicate 3)
28
14

Fig.7b

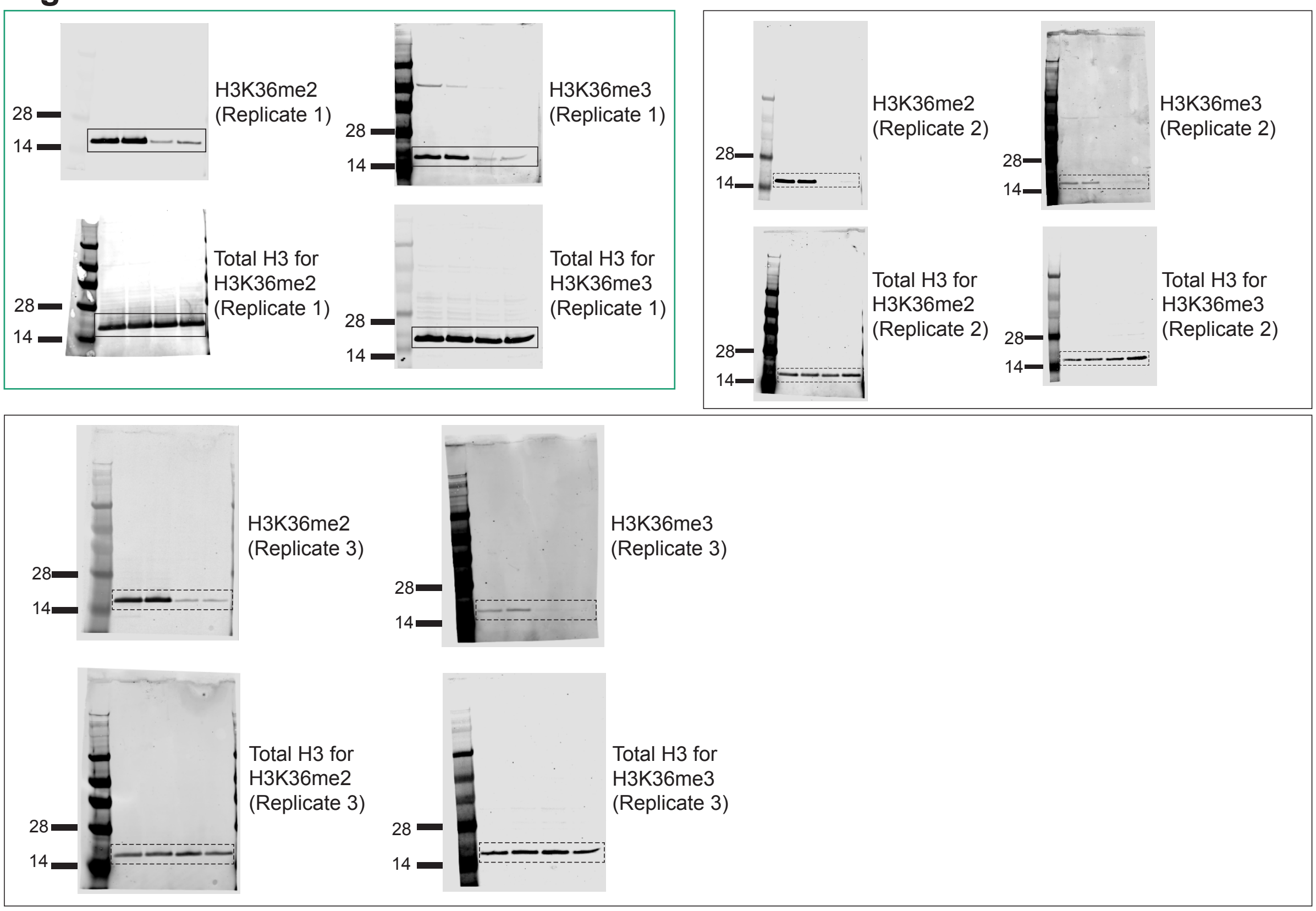
H3K36me2
(Replicate 1)
H3K36me3
(Replicate 1)
Total H3 for
H3K36me2
(Replicate 1)
Total H3 for
H3K36me3
(Replicate 1)
H3K36me2
(Replicate 2)
H3K36me3
(Replicate 2)
Total H3 for
H3K36me2
(Replicate 2)
Total H3 for
H3K36me3
(Replicate 2)
H3K36me2
(Replicate 3)
H3K36me3
(Replicate 3)
Total H3 for
H3K36me2
(Replicate 3)
Total H3 for
H3K36me3
(Replicate 3)
28
14

Fig.7c

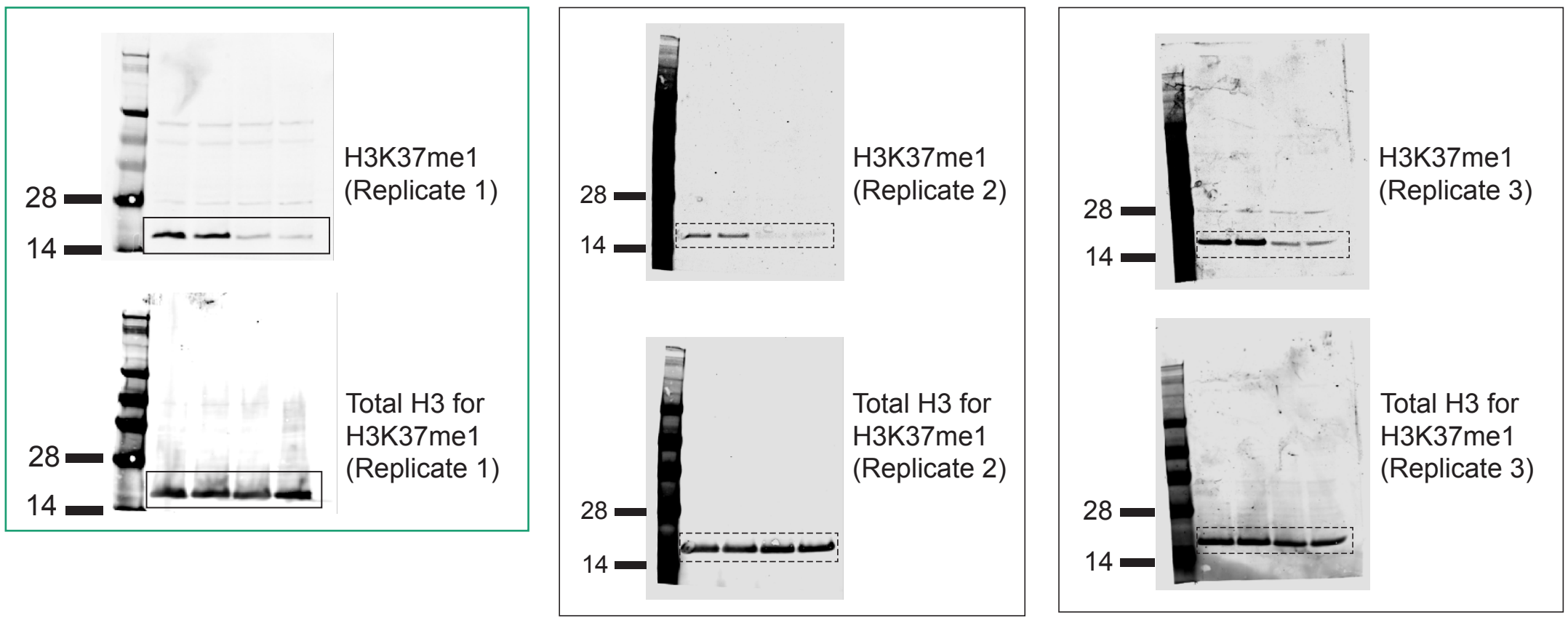

Fig.7d

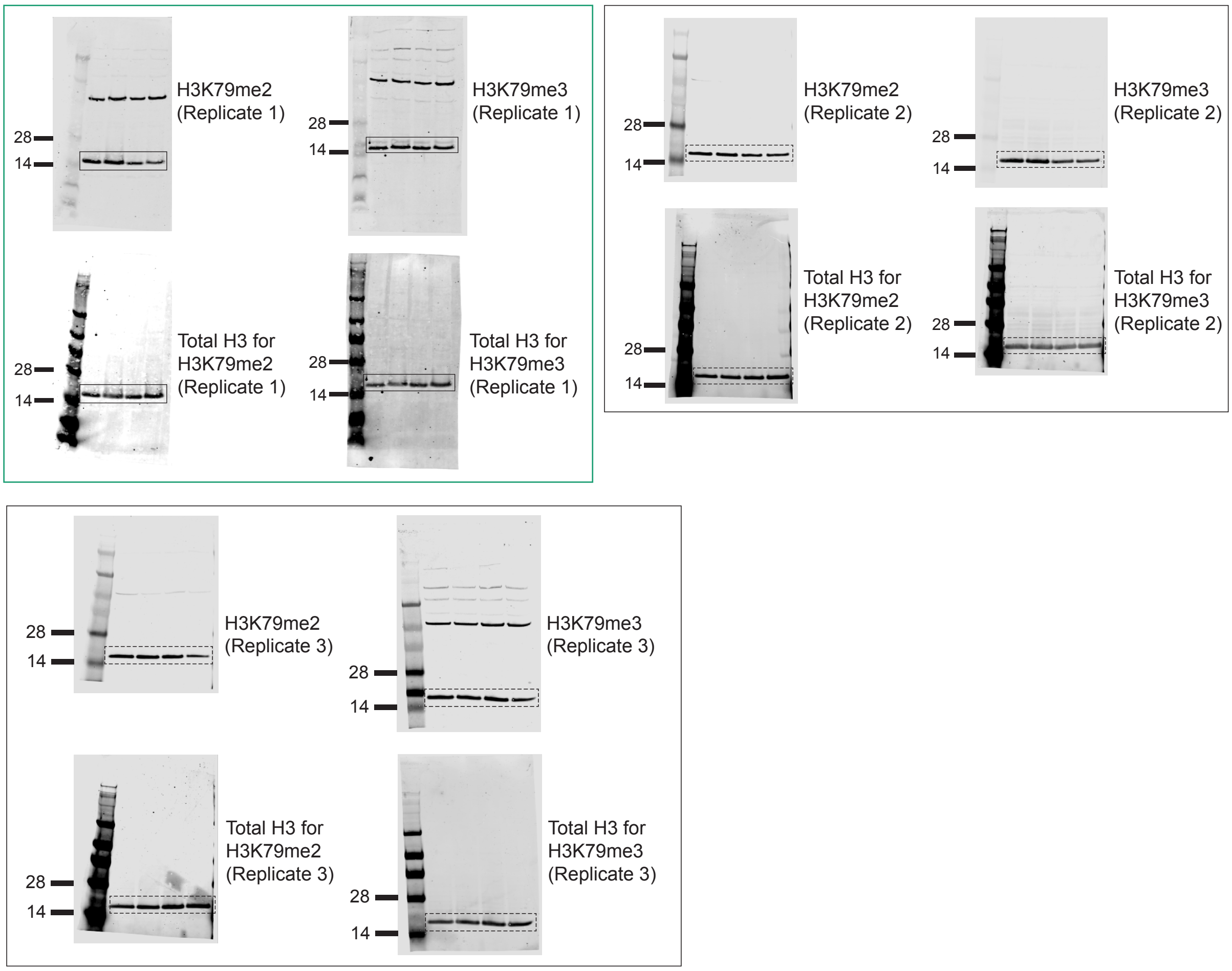

Fig.7h

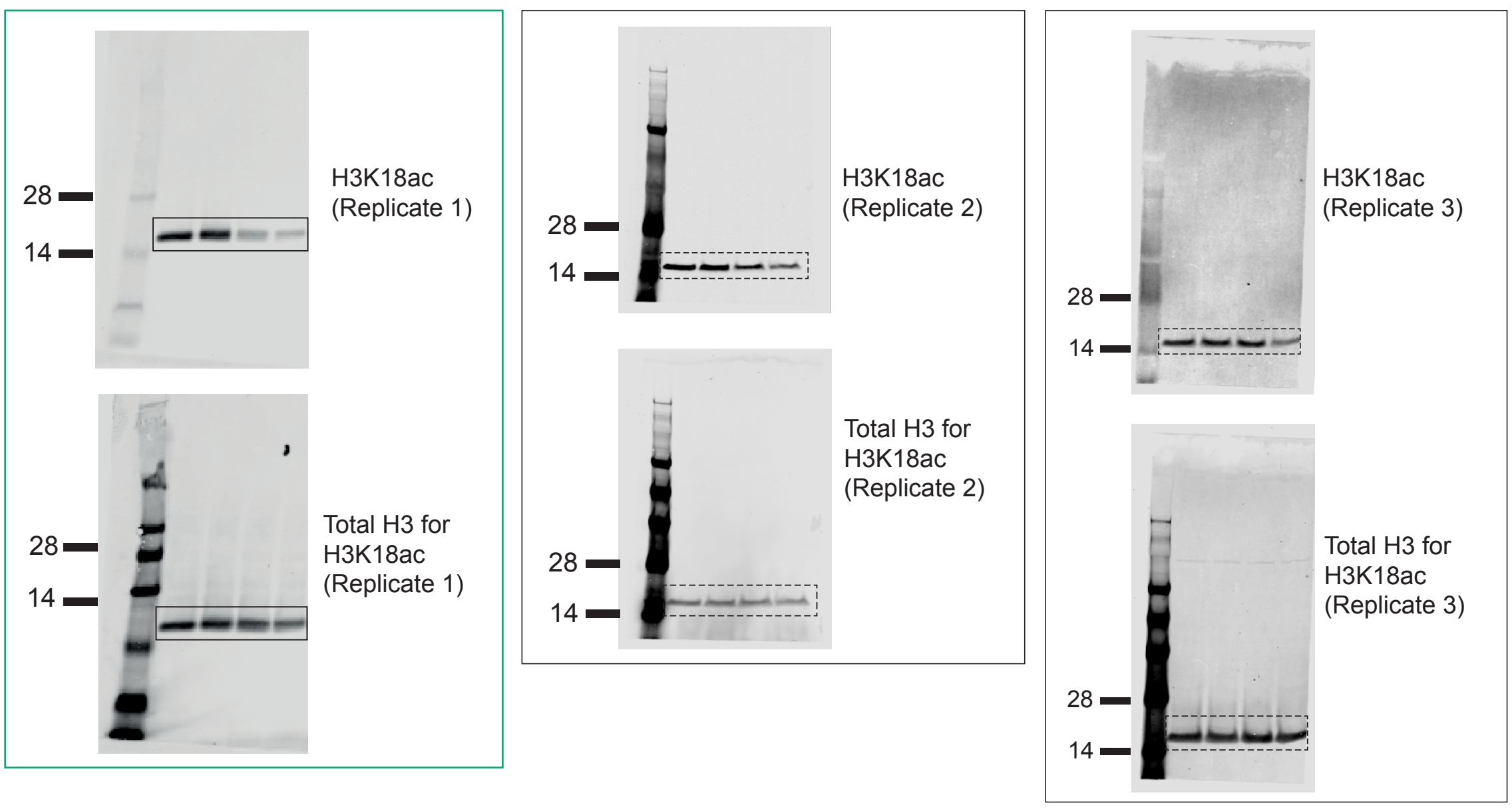

Supplement: Supplementary file 5 [file 41588_2026_2675_MOESM16_ESM.pdf]
