# Supplementary material for: Identifying critical lysines in mammalian histone H3 with high-throughput CRISPR prime editing
Source: Nat Genet. 2026 Jul 8;58(9):2303–19. doi: 10.1038/s41588-026-02675-y (PMC13553292; doi:10.1038/s41588-026-02675-y)
Supplement: Supplementary file 7 [file 41588_2026_2675_MOESM1_ESM.pdf]

nature portfolio

Corresponding author(s): Aliaksandra Radzisheuskaya

Last updated by author(s): May 27, 2026

Reporting Summary

Nature Portfolio wishes to improve the reproducibility of the work that we publish. This form provides structure for consistency and transparency in reporting. For further information on Nature Portfolio policies, see our Editorial Policies and the Editorial Policy Checklist.

Statistics

For all statistical analyses, confirm that the following items are present in the figure legend, table legend, main text, or Methods section.

n/a	Confirmed	
☐	☒	The exact sample size (*n*) for each experimental group/condition, given as a discrete number and unit of measurement
☐	☒	A statement on whether measurements were taken from distinct samples or whether the same sample was measured repeatedly
☐	☒	The statistical test(s) used AND whether they are one- or two-sided<br>*Only common tests should be described solely by name; describe more complex techniques in the Methods section.*
☐	☒	A description of all covariates tested
☐	☒	A description of any assumptions or corrections, such as tests of normality and adjustment for multiple comparisons
☐	☒	A full description of the statistical parameters including central tendency (e.g. means) or other basic estimates (e.g. regression coefficient) AND variation (e.g. standard deviation) or associated estimates of uncertainty (e.g. confidence intervals)
☐	☒	For null hypothesis testing, the test statistic (e.g. *F*, *t*, *r*) with confidence intervals, effect sizes, degrees of freedom and *P* value noted<br>*Give P values as exact values whenever suitable.*
☒	☐	For Bayesian analysis, information on the choice of priors and Markov chain Monte Carlo settings
☐	☒	For hierarchical and complex designs, identification of the appropriate level for tests and full reporting of outcomes
☒	☐	Estimates of effect sizes (e.g. Cohen's *d*, Pearson's *r*), indicating how they were calculated

Our web collection on statistics for biologists contains articles on many of the points above.

Software and code

Policy information about availability of computer code

Data collection	Sony MA900 cell sorter<br>Agilent Tapestation 4150<br>Illumina MiSeq<br>Illumina NextSeq 1000/2000<br>QuantStudio 7 Pro<br>Odyssey M scanner<br>EVOS M5000<br>Countess 3 FL automated cell counter<br>Ultimate 3000 RSLCnano system<br>Orbitrap Exploris 480
Data analysis	PrimeDesign — v0.2<br>DeepPrime — v3.6<br>CRISPResso2 — v2.3.4<br>CopyCaller — v2.1<br>Image Studio — v5.2<br>Cutadapt — v4.9<br>Bowtie2 — v2.5.4<br>samtools — v1.21<br>deepTools — v3.5.5<br>IGV — v2.19.7

ACE — v1.30.0
Opentrons Protocol Designer — v8.3.0
bedtools — v2.31.1
EaSeq — v1.2
MACS3 — v3.0.3
HiSat2 — v2.2.1
DESeq2 — v1.52.0
DAVID — 2025 release
Skyline — v25.1
Proteome Discoverer — v2.2

For manuscripts utilizing custom algorithms or software that are central to the research but not yet described in published literature, software must be made available to editors and reviewers. We strongly encourage code deposition in a community repository (e.g. GitHub). See the Nature Portfolio guidelines for submitting code & software for further information.

Data

Policy information about availability of data

All manuscripts must include a data availability statement. This statement should provide the following information, where applicable:

- Accession codes, unique identifiers, or web links for publicly available datasets
- A description of any restrictions on data availability
- For clinical datasets or third party data, please ensure that the statement adheres to our policy

The amplicon-based and whole-genome NGS data were submitted to SRA (PRJNA1268213). The CUT&Tag, CUT&RUN and RNA-seq data were submitted to GEO (CUT&Tag: GSE319420, CUT&RUN: GSE319506, RNA-seq: GSE319059). Source data are provided with this paper.

Research involving human participants, their data, or biological material

Policy information about studies with human participants or human data. See also policy information about sex, gender (identity/presentation), and sexual orientation and race, ethnicity and racism.

Reporting on sex and gender	Not applicable
Reporting on race, ethnicity, or other socially relevant groupings	Not applicable
Population characteristics	Not applicable
Recruitment	Not applicable
Ethics oversight	Not applicable

Note that full information on the approval of the study protocol must also be provided in the manuscript.

Field-specific reporting

Please select the one below that is the best fit for your research. If you are not sure, read the appropriate sections before making your selection.

☒ Life sciences ☐ Behavioural & social sciences ☐ Ecological, evolutionary & environmental sciences

For a reference copy of the document with all sections, see nature.com/documents/nr-reporting-summary-flat.pdf

Life sciences study design

All studies must disclose on these points even when the disclosure is negative.

Sample size	Sample sizes are indicated in the figure legends and Supplementary Table 3 and were selected based on established practices in the field.
Data exclusions	Samples with fewer than 1000 aligned reads in amplicon-based NGS were excluded from the analysis
Replication	All the presented experiments were performed with the minimum of two independent biological replicates. The exact nature of the biological replication (e.g., independent transfections or independently derived clones) is clearly stated in the figure legends and Supplementary Table 3.
Randomization	Randomization was not performed because samples were assigned based on predefined experimental groups rather than through randomized allocation.
Blinding	No blinding was performed in the study. Blinding was not applicable in this study because the analyses generated objective, directly measurable results that did not rely on subjective interpretation or assessment by the investigators.

Reporting for specific materials, systems and methods

We require information from authors about some types of materials, experimental systems and methods used in many studies. Here, indicate whether each material, system or method listed is relevant to your study. If you are not sure if a list item applies to your research, read the appropriate section before selecting a response.

Materials & experimental systems

n/a	Involved in the study
☐	☒ Antibodies
☐	☒ Eukaryotic cell lines
☒	☐ Palaeontology and archaeology
☒	☐ Animals and other organisms
☒	☐ Clinical data
☒	☐ Dual use research of concern
☒	☐ Plants

Methods

n/a	Involved in the study
☒	☐ ChIP-seq
☐	☒ Flow cytometry
☒	☐ MRI-based neuroimaging

Antibodies

Antibodies used

H3 CUSABIO CSB-MA000308 1:5,000
H3 Abcam ab201456 1:2,000
H3K4me1 EpiCypher 13-0057 1:1,000
H3K4me3 Cell Signaling Technology 9751 1:1,000
H3K9me1 Abcam ab9045 1:2,500
H3K9me2 Abcam ab1220 1:2,500
H3K9me3 Abcam ab176916 1:2,500
H3K27me2 Cell Signaling Technology 9728 1:2,500
H3K27me3 Cell Signaling Technology 9733 1:2,500 WB, 1:100 C&T
H3K27ac Invitrogen MA5-23516 1:1,000
H3K36me2 Thermo Fisher Scientific MA5-14867 1:1,000
H3K36me3 Acitve Motif 61021 1:3,000 WB; 1:50 C&T
H3K37me1 Abcam ab215728 1:2,500
H3K79me2 Abcam ab3594 1:2,500
H3K79me3 Abcam ab2621 1:2,500
H3K14ac Abcam ab52946 1:2,500
H3K18ac Cell Signaling Technology 13998 1:1,000 WB; 1:50 C&T
H3K23ac (WB) Cell Signaling Technology 14932 1:1,000
H3K23ac (CUT&RUN) Sigma/Millipore 07-355 1:50
H3.3 Active Motif 91191 1:50
Goat anti-mouse Licor Biosciences 925-68070 1:20,000
Goat anti-rabbit Licor Biosciences 925-32211 1:20,000

Validation

H3 CUSABIO CSB-MA000308 Expected band size
H3 Abcam ab201456 Expected band size
H3K4me1 EpiCypher 13-0057 SNAP-ChIP
H3K4me3 Cell Signaling Technology 9751 PMID: 36859550
H3K9me1 Abcam ab9045 Small peptide test by Abcam
H3K9me2 Abcam ab1220 Peptide array by Abcam
H3K9me3 Abcam ab176916 https://chromatinantibodies.com
H3K27me2 Cell Signaling Technology 9728 https://chromatinantibodies.com and PMID: 35668298
H3K27me3 Cell Signaling Technology 9733 https://chromatinantibodies.com and PMID: 35668298
H3K27ac Invitrogen MA5-23516 https://chromatinantibodies.com and PMID: 35668298
H3K36me2 Thermo Fisher Scientific MA5-14867 https://chromatinantibodies.com and PMID: 37402365
H3K36me3 Acitve Motif 61021 PMID: 37402365
H3K37me1 Abcam ab215728 Peptide array by Abcam
H3K79me2 Abcam ab3594 Peptide array by Abcam and PMID: 40009485
H3K79me3 Abcam ab2621 Luminex testing in collaboration with Epicypher, unpublished
H3K14ac Abcam ab52946 SNAP-ChIP, this study
H3K18ac Cell Signaling Technology 13998 SNAP-ChIP, this study
H3K23ac (WB) Cell Signaling Technology 14932 SNAP-ChIP, own data, unpublished
H3K23ac (CUT&RUN) Sigma/Millipore 07-355 SNAP-ChIP, own data, unpublished
H3.3 Active Motif 91191 PMID: 39908143

Eukaryotic cell lines

Policy information about cell lines and Sex and Gender in Research

Cell line source(s)

We used E14Tg2a mouse embryonic cell line, which we obtained from the laboratory of Prof Joshua Brickman, University of

Cell line source(s)	Copenhagen
Authentication	No authentication was performed
Mycoplasma contamination	The mouse embryonic stem cells are routinely tested for mycoplasma on a bi-monthly basis and are negative.
Commonly misidentified lines (See ICLAC register)	None

Plants

Seed stocks	*Report on the source of all seed stocks or other plant material used. If applicable, state the seed stock centre and catalogue number. If plant specimens were collected from the field, describe the collection location, date and sampling procedures.*
Novel plant genotypes	*Describe the methods by which all novel plant genotypes were produced. This includes those generated by transgenic approaches, gene editing, chemical/radiation-based mutagenesis and hybridization. For transgenic lines, describe the transformation method, the number of independent lines analyzed and the generation upon which experiments were performed. For gene-edited lines, describe the editor used, the endogenous sequence targeted for editing, the targeting guide RNA sequence (if applicable) and how the editor was applied.*
Authentication	*Describe any authentication procedures for each seed stock used or novel genotype generated. Describe any experiments used to assess the effect of a mutation and, where applicable, how potential secondary effects (e.g. second site T-DNA insertions, mosiacism, off-target gene editing) were examined.*

Flow Cytometry

Plots

Confirm that:

☒ The axis labels state the marker and fluorochrome used (e.g. CD4-FITC).

☒ The axis scales are clearly visible. Include numbers along axes only for bottom left plot of group (a 'group' is an analysis of identical markers).

☐ All plots are contour plots with outliers or pseudocolor plots.

☒ A numerical value for number of cells or percentage (with statistics) is provided.

Methodology

Sample preparation	The cells were treated with doxycycline for 48 hours before the analysis
Instrument	CytoFLEX, SONY MA900
Software	FlowJo
Cell population abundance	The target population were >30% abundant
Gating strategy	Cells were initially gated based on forward and side scatter (FSC/SSC) profiles to exclude debris and select single cells.

☐ Tick this box to confirm that a figure exemplifying the gating strategy is provided in the Supplementary Information.
