# Supplementary material for: Identifying critical lysines in mammalian histone H3 with high-throughput CRISPR prime editing
Source: Nat Genet. 2026 Jul 8;58(9):2303–19. doi: 10.1038/s41588-026-02675-y (PMC13553292; doi:10.1038/s41588-026-02675-y)

Extended Data Fig.5n

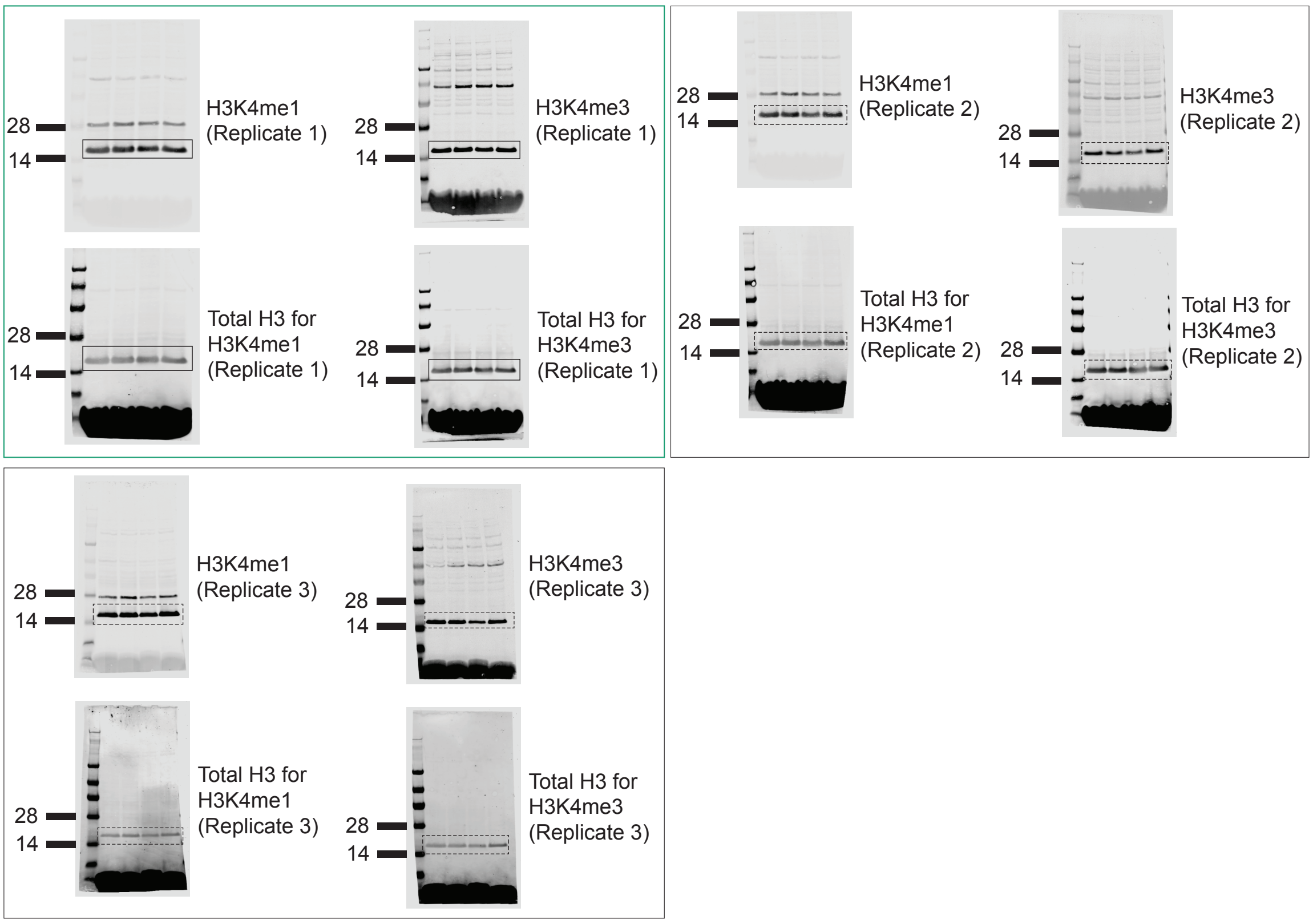

Supplement: Supplementary file 8 [file 41588_2026_2675_MOESM25_ESM.pdf]
