# Supplementary material for: Identifying critical lysines in mammalian histone H3 with high-throughput CRISPR prime editing
Source: Nat Genet. 2026 Jul 8;58(9):2303–19. doi: 10.1038/s41588-026-02675-y (PMC13553292; doi:10.1038/s41588-026-02675-y)
Supplement: Supplementary file 11 [file 41588_2026_2675_MOESM2_ESM.pdf]

nature portfolio

Peer Review File

Identifying Critical Lysines in Mammalian Histone H3 with High-Throughput CRISPR Prime Editing

Corresponding Author: Dr Aliaksandra (Alex) Radzisheuskaya

A version of this paper was originally rejected for publication by Nature Genetics, however that decision was reconsidered after appeal by the authors.

Version 0:

Decision Letter:

9th Sep 2025

Dear Dr Radzisheuskaya,

first, I'd like to sincerely apologize for the delay in providing our decision. Thank you very much for your patience during this process.

Your Article entitled "Uncovering Critical Lysines in Mammalian Histone H3 with High-Throughput CRISPR Prime Editing" has now been seen by 3 referees, whose comments are attached. While they find your work of potential interest, they have raised serious concerns which in our view are sufficiently important that they preclude publication of the work in Nature Genetics, at least in its present form.

While the referees find your work of some interest with Reviewer #1 and #3 both acknowledging the methodological innovation and effort, they raised concerns regarding the level of biological novelty and mechanistic insights provided and some potential limitations (ie, inconsistent efficiency of editing across residues; lack of quantification of WB; lack of statistics).

Should further experimental data allow you to fully address these criticisms we would be willing to consider an appeal of our decision (unless, of course, something similar has by then been accepted at Nature Genetics or appeared elsewhere). This includes submission or publication of a portion of this work someplace else.

The required new experiments and data include, but are not limited to those detailed here. We hope you understand that until we have read the revised manuscript in its entirety we cannot promise that it will be sent back for peer review.

If you are interested in attempting to revise this manuscript for submission to Nature Genetics in the future, please contact me to discuss a potential appeal. Otherwise, we hope that you find our referees' comments helpful when preparing your manuscript for resubmission elsewhere.

Thank you.

Sincerely,
Chiara

Chiara Anania, PhD
Associate Editor
Nature Genetics
https://orcid.org/0000-0003-1549-4157

Referee expertise:

Referee #1: epigenetics, histone modifications

Referee #2: epigenetics, chromatin and transcription

Referee #3: epigenetics, histones

Reviewers' Comments:

Reviewer #1 (Remarks to the Author):

In this study, prime editing tools are applied to precisely mutate particular H3 lysines simultaneously in all H3 gene copies in mESCs. Published work addressing the function or importance of specific histone lysine PTMs usually target histone modifying enzymes, however this approach is complicated by off-target effects and compensation. An alternative approach is to directly target the histone genes themselves but this is technically challenging. The authors describe a suite of approaches to target all H3 lysines in all genomic copies in mESCs, combine them or revert them, estimate and minimize off-target mutations, and go on to identify essential H3 lysines in mESCs.

Over the past few years there have been a number of publications raising questions about the functional importance of histone modifications in mammalian systems, with recent results indicating that H3K27ac is not crucial in mouse ESCs early differentiation. In addition, catalytic mutations in the enzymes that carry out modifications indicate that this function may not be, alone, crucial for function. Thus, the question arises: are histone modifications essential, and how much redundancy might there be that might explain lack of essentiality of single sites? The results of this study provide robust evidence indicating that certain acetylation sites in histones are required for viability, and thus the study adds important and valuable information revealing that histone modifications can be essential.

The authors provide a strong methodological approach to edit histone genes, including recommendations to optimize editing rate and minimize deletions using prime base editing. The work is technically robust and thorough, and the manuscript is well-written and organized. Overall, they examine all 13 lysines in histone H3 and identify 5 as critical for cellular fitness. The identification of which H3 lysines are essential is of great interest. However, given this complete set of K mutations in H3, and the likelihood that some sites are redundant, one further step to evaluate redundant function should be carried out – at least with H3K27.

Specific comments:

1. Most importantly, they generate 13 mESC lines of K-to-R mutations at all Ks in H3, and identify 5 as required for cellular fitness, establishing that key sites are individually critical. This goes far beyond the finding that H3K27ac is not critical in mESCs (Helin lab), which was not surprising given that CBP/p300 histone acetyltransferase is well-known to target many sites in histones, and that other sites are more robust than H3K27 (Choudhary's lab). Thus, given the evolutionary conservation, the results of this study establish a foundation in H3 lysine analysis to build upon to reveal roles of individual and redundant histone sites.
2. Fig 2: The authors make the reasonable assertion that failure to generate complete K14R and K18R clones, while K-to-K clones are generated efficiently, indicates that there is a fitness penalty to mutating those lysines. However, loss of fitness upon H3K14 and H3K18 mutation was not specifically demonstrated. To confirm essentiality in mESCs, editing should be done in the presence of a recombinant H3 engineered to harbour mutations to protect it from mutagenesis. After editing is complete, the recombinant H3 can be excised using a FLP, LoxP, or similar system, to then experimentally demonstrate loss of fitness. In short, incomplete K-to-R editing is used as a proxy for loss of cellular fitness throughout and is a basis for the title of the manuscript, so it is essential to confirm this.
3. Fig 4g: ChIP-seq would be informative, to understand whether acetylation is being lost in the H3.1/2/3 clones at particular loci, whether H3.3 is more frequently acetylated and/or redistributed in the H3.1/2 clones, and to understand how histone variants might be compensating for each other as suggested in the text lines 269-270.
4. Fig 5c (& e): Since the data is not expected to be normally distributed, t-test is inappropriate. The data show increased copy number loss in the -nickRNA K18R column, from 25% to 71%, in contrast to the assertion in line 292 that there is little impact, and lines 311 and 434, that omitting nickRNA will minimize large deletions. The authors should reconcile their conclusions with the K18R copy number data in 5c. Or, perform additional replicates, if the data in 5c is believed to have the same distribution as 'K18 K-to-R' in Fig 5e.
5. Fig 5: Is there a correlation between % editing (such as in Fig 1i) and the frequency of large or small deletions? Would they be defined as indels?
6. Fig 6: Is there a correlation between how essential each lysine is with frequency that they are mutated in developmental disease or cancer, given the extensive sequence information available for histone mutations?
7. Fig 6: Conclusions of the study, especially in light of previous results mutating the single H3 K27, to examine whether simultaneous loss of two non-essential lysines lead to fitness deficits, and in particular, of K27. This will provide important information about functional redundancy of histone modifications.
8. Line 382: Was a standard curve included for the Western blots? If not, should rewrite to "3-4-fold decrease in Western blot signal", or similar.
9.Line 447: Suggestions for mitigating this suboptimal outcome, or predicting whether/why a locus would be impacted?

Reviewer #2 (Remarks to the Author):

This manuscript reports the use of a CRISPR prime editing-based approach for histone gene mutagenesis. While technically sound, the study is primarily methodological in nature and lacks novel biological insights. All histone modification assessments were performed by Western blot analysis, which is not sufficiently quantitative; more robust techniques such as mass spectrometric methods and/or ChIP-seq are needed to accurately measure modification levels and loci specificity. Moreover, the editing efficiency varies significantly across different lysine residues (e.g., H3K4), making it difficult to draw meaningful conclusions about correlations with cellular fitness. Additionally, single-site lysine-to-arginine (K→R) mutations may have limited interpretability due to compensatory effects, histone mark crosstalk, and the presence of other modifications (e.g., methylation or acetylation) on the same residue. Incomplete editing across histone variants (H3.1, H3.2, and H3.3) further complicates interpretation. Overall, given the lack of new biological discoveries and the methodological limitations,the manuscript is not suitable for publication in Nature Genetics.

Reviewer #3 (Remarks to the Author):

General summary and opinion

In the present study, Price et al. present a high-throughput CRISPR prime editing platform that enables the precise, base-resolution mutation of lysine residues across all canonical and variant histone H3 genes in mouse embryonic stem cells. Using this system, the authors introduce lysine-to-arginine substitutions, paired with silent lysine-to-lysine controls, to dissect the contribution of individual lysine residues to cellular fitness and chromatin regulation. They systematically map post-translational modification outcomes and fitness consequences across a comprehensive series of histone mutants.
This work show that the K-R mutation at several lysines (H3K4, H3K9, H3K14, H3K18, and H3K79) result in viability defects, consistent with known essential roles for their corresponding PTMs. In contrast, other residues, including H3K23, H3K36, H3K56, and H3K122, appear to be dispensable in self-renewing mESCs. They validate editing efficiency, monitor loss of specific marks, and relate some of these perturbations to cell growth phenotypes. Additional mechanistic depth is explored via chromatin mark recovery.
This study builds on prior approaches in yeast and Drosophila and more recent histone knockout systems, but offers technical advance: the ability to study individual histone lysines in their native genomic context, without reliance on transgenes or exogenous histone supply. The methodological execution is careful, and the platform itself is clearly powerful, both scalable and adaptable for future applications such as modeling disease-associated oncohistones or interrogating PTM crosstalk.
However, while the technical achievements are significant, the biological insight drawn from these experiments is at times underdeveloped. Several findings reinforce existing knowledge rather than breaking conceptual ground, and key results, such as the failure to efficiently mutate H3K4, are insufficiently addressed. Furthermore, figure presentation is inconsistent, with a lack of quantification across key western blots and missing statistical annotation for multiple panels. Additionally, the discussion does not fully explore developmental implications of histone lysine function in mESCs, or the potential biological depth.
While this work could serve as a foundation for future efforts to map and manipulate the histone code in mammalian systems, the manuscript would benefit from clearer data presentation, a more expanded discussion of key mechanistic insights, and clarification of a few experimental choices. Below, we outline a series of major and minor points intended to help strengthen the impact and clarity of the study.

Major concerns

(1) Lack of functional combinatorial analysis
The authors provide a demonstration of combinatorial editing capability by designing a single epegRNA to simultaneously target H3K14, H3K18, and H3K23 (Fig. 3e-g). This is an important proof-of-principle, showing that the platform can introduce multiple lysine mutations in cis within a single histone tail using prime editing. The successful editing of the triple K-K and partial editing of the triple K-R construct illustrate the feasibility of multi-site editing, even within a short target window. However, this experiment remains a demonstration of editing feasibility, rather than a functional analysis of combinatorial histone modification since (1) the triple mutant is assessed only at the DNA level in pooled cells, without clonal isolation, western blot validation, or phenotypic characterization and (2) since K14R and K18R are independently deleterious, the strong reduction in triple K-R reads is expected and does not allow for functional dissection of crosstalk or synthetic interactions. Therefore, we suggest including a designed double/triple mutant involving at least one tolerated residue (for example K27R+K36R or even K27R+K36R+K37R), with some degree of phenotypic or biochemical readout (histone PTM levels, growth) to proof the combinatorial analysis system is functionally flexible.

(2) Quantification of western blots and biological replicates
Most western blot gels (Figures 1j, 3d, 4c, 4g) are presented without quantification or mention of biological replicates. This limits interpretability, especially where the same histone mark in the same line is being compared across multiple panels or clones. In some cases, exposure differences lead to apparent inconsistencies (1j vs. 3d vs. 4c for H3K23ac).
- Please include normalization against loading control (using global anti-H3) across all WB panels.
- Please include quantification (mean ± SD) from at least three biological replicates across all WB panels.
- Clarify whether loading controls were consistent and whether the same gel and exposure conditions were used across panels.

(3) Interpretation of H3K4 mutants

Despite H3K4 being one of the most well-studied histone lysine residues, it is not represented in the main figures and receives only limited attention in the extended data figure 5, where the authors report low editing efficiencies for both the K-R mutation and the synonymous K-K control. The fact that the silent mutation results in unsuccessful editing may point to technical difficulties, such as pegRNA efficiency or sequence context, rather than selection against mutation. They mention this in the results section (lines 256-271). Nevertheless, in both abstract and discussion sections they repeatedly refers to H3K4 as one of the most deleterious or essential residues, along with well-substantiated cases like H3K18 or H3K79. These statements are difficult to reconcile with the limited data available.
Additionally, from the results showed in Extended figure 5g we can observe that there is at least one clone with ~20% H3K4R editing. We suggest this clone could be easily analysed for at least histone mark abundance to help clarify this issue.
To improve the interpretive consistency of the manuscript, we suggest the authors:
- Clarify their conclusions regarding the essentiality of H3K4.
- Discuss in more depth the unexpected outcome of the K-K editing attempt.
- Consider presenting any data from the partially edited clone, or comment on why further analysis was not pursued.

(4) Deepening the biological context in the discussion
The discussion does not engage with the broader biological implications of the findings and is very results-oriented, particularly for residues described as non-essential based on mESC proliferation. Several lysines, including H3K36, K56, and K122, have established roles in transcriptional regulation, nucleosome dynamics, and genome stability. That mutations at these sites are tolerated under steady-state ESC culture is interesting, but it raises important questions about their potential roles in other contexts, such as during differentiation or in response to stress. Additionally, the observations on a relevant mutation like H3K27R aligns with previous data on PRC2 dispensability for self-renewal, but lacks deeper discussion on the role of this mark in proper lineage specification. A more focused discussion of these known biological functions, and how this system could be applied to tackle them in future work would strengthen the manuscript and better reflect the significance of the data.

Minor concerns

(1) Figure 1h/i/j: Please indicate in a different color in the lines from 1h that are analyzed in subsequent panels to help readers track clones across figures.
(2) In Figure 7e it is unclear that the mark measured is H3K18ac, clearly indicate.
(3) The figure legend for 7e says 48h treatment, but the x-axis extends to 144h. Clarify.
(4) Extended Figure 6: The data on modifying enzymes are relevant. We suggest moving some of this content into the main figures.
(5) Clone selection in figure 2 requires justification. Clone K18R_3 shows the highest editing efficiency (>40%) yet is not used in subsequent experiments. This raises the question of whether clone selection was biased or whether certain clones were excluded for non-obvious reasons.
(6) H3.3 is central to the experimental strategy but is not introduced until late in the results section (lines 241-247). Please include a brief summary of its biological role earlier in the introduction to help contextualize why it's edited separately.
(7) Rethink the use of the term "essential" and maybe temper to "strongly selected against" unless full lethality is shown.
(8) The manuscript lacks clear and consistent reporting of statistical tests used and how error bars were calculated. Examples: Figures 3g, 7g.
(9) Data are claimed to be uploaded to SRA but accession number is missing.
(10) The suggestion that H3.3 compensates for mutated canonical histones in partially edited clones is plausible but speculative. We recommend briefly discussing this hypothesis in the discussion, noting its limitations and referencing available data on H3.3 genomic localization or dynamics.
(11) The study assumes proper chromatin incorporation of mutant histones (K18R, K56R) without direct biochemical validation. We suggest discussing the different approaches (histone acid extraction, immunoblotting, mass spec) that could be used to confirm incorporation levels, which would clarify interpretation.
(12) In K9R mutants (differential changes between acetylation and methylation) they should check or discuss the impact of differential H3.3K9me/H3.1K9me and H3.1/H3.3K9ac.
(13) When referencing future applications, such as modeling disease mutations (H3K27M, H3K36M), the authors should cite papers such as (Schwartzentruber et al., Nature, 2012; Wu et al., Nature Genetics, 2012; Behjati et al., Nature Genetics, 2013) to ground the discussion.
(14) A brief sentence in the discussion projecting broader uses of the platform (editing other histone types (H4, H2A, H2B) or targeting other repetitive gene families) would help clarify the system's potential utility.
(15) While the Materials and Methods section is generally well organized, several important details are missing.
- The number of biological replicates is not consistently stated for key experiments.
- Statistical methods used to analyze and compare datasets are not described in the methods section.
- The western blot methodology lacks detail: for example, on antibody validation, normalization protocol, and quantification tools.

Version 1:

Decision Letter:

Our ref: NG-A69174R-Z

4th May 2026

Dear Dr. Radzisheuskaya,

Thank you for submitting your revised manuscript "Uncovering Critical Lysines in Mammalian Histone H3 with High-Throughput CRISPR Prime Editing" (NG-A69174R-Z). It has now been seen by the original referees and their comments are below. The reviewers find that the paper has improved in revision, and therefore we'll be happy in principle to publish it in Nature Genetics, pending minor revisions to comply with our editorial and formatting guidelines.

If the current version of your manuscript is in a PDF format, please email us a copy of the file in an editable format (Microsoft Word or LaTex)-- we can not proceed with PDFs at this stage.

We are now performing detailed checks on your paper and will send you a checklist detailing our editorial and formatting requirements soon. Please do not upload the final materials and make any revisions until you receive this additional information from us.

Thank you again for your interest in Nature Genetics Please do not hesitate to contact me if you have any questions.

Congratulations!

Sincerely,
Chiara

Chiara Anania, PhD
Senior Editor
Nature Genetics
https://orcid.org/0000-0003-1549-4157

Reviewer #1 (Remarks to the Author):

We think this is an excellent rebuttal and the authors have addressed our concerns. We do not require additional revision.

Our main request to improve rigor in point 2, where we had a problem with them conflating “we cannot make cell lines carrying mutations of this lysine” to “this lysine is essential” without direct demonstration. This was also raised by reviewer #3. They made a good faith effort to do the requested experiment, which did not work and so instead softened their language. We think this is acceptable.

With respect to novel biological significance, the paper’s contribution is their identification of which H3 lysines are essential vs. dispensable for mESC self-renewal. It will probably be cited often for this point, so we acknowledge that this is good enough.

We appreciate the double mutant data they introduce to our point 7, including the RNA-seq analysis and their pointing out the transcriptome rewiring that appears (or is reversed) only in the double mutants. They do a good job arguing that it demonstrates biological significance.

Reviewer #3 (Remarks to the Author):

We thank the authors for their detailed responses. They have made a substantial effort to address our concerns by adding appropriate controls and quantifications, and by performing additional experiments requested by all reviewers (including sequential mutagenesis, double-mutant RNA-seq, CUT&Tag profiling, and mass spectrometry), which significantly strengthen the manuscript.

The new data further support the main conclusion . Interestingly, it shows that combinatorial perturbations (such as H3K27R+H3K36R double mutant) show impairments in self - renewal , whereas the corresponding single substitutions have only modest effects. This work highlights unique regulatory crosstalk that would not be evident from single-site perturbations alone.

The revised Discussion now provide a proper contextualiz ation of the findings and attention has been paid to avoid overstatement.

In conclusion, we believe this work will serve as a foundation for future efforts to map and manipulate the histone code. Therefore, we support its publication in Nature Genetics.

Open Access This Peer Review File is licensed under a Creative Commons Attribution 4.0 International License, which permits use, sharing, adaptation, distribution and reproduction in any medium or format, as long as you give appropriate credit to the original author(s) and the source, provide a link to the Creative Commons license, and indicate if changes were made.
In cases where reviewers are anonymous, credit should be given to 'Anonymous Referee' and the source.
The images or other third party material in this Peer Review File are included in the article's Creative Commons license, unless indicated otherwise in a credit line to the material. If material is not included in the article's Creative Commons license and your intended use is not permitted by statutory regulation or exceeds the permitted use, you will need to obtain permission directly from the copyright holder.
To view a copy of this license, visit https://creativecommons.org/licenses/by/4.0/

Reviewers' Comments:

Reviewer #1 (Remarks to the Author):

In this study, prime editing tools are applied to precisely mutate particular H3 lysines simultaneously in all H3 gene copies in mESCs. Published work addressing the function or importance of specific histone lysine PTMs usually target histone modifying enzymes, however this approach is complicated by off-target effects and compensation. An alternative approach is to directly target the histone genes themselves but this is technically challenging. The authors describe a suite of approaches to target all H3 lysines in all genomic copies in mESCs, combine them or revert them, estimate and minimize off-target mutations, and go on to identify essential H3 lysines in mESCs.

Over the past few years there have been a number of publications raising questions about the functional importance of histone modifications in mammalian systems, with recent results indicating that H3K27ac is not crucial in mouse ESCs early differentiation. In addition, catalytic mutations in the enzymes that carry out modifications indicate that this function may not be, alone, crucial for function. Thus, the question arises: are histone modifications essential, and how much redundancy might there be that might explain lack of essentiality of single sites? The results of this study provide robust evidence indicating that certain acetylation sites in histones are required for viability, and thus the study adds important and valuable information revealing that histone modifications can be essential.

The authors provide a strong methodological approach to edit histone genes, including recommendations to optimize editing rate and minimize deletions using prime base editing. The work is technically robust and thorough, and the manuscript is well-written and organized. Overall, they examine all 13 lysines in histone H3 and identify 5 as critical for cellular fitness. The identification of which H3 lysines are essential is of great interest. However, given this complete set of K mutations in H3, and the likelihood that some sites are redundant, one further step to evaluate redundant function should be carried out – at least with H3K27.

Specific comments:

1. Most importantly, they generate 13 mESC lines of K-to-R mutations at all Ks in H3, and identify 5 as required for cellular fitness, establishing that key sites are individually critical. This goes far beyond the finding that H3K27ac is not critical in mESCs (Helin lab), which was not surprising given that CBP/p300 histone acetyltransferase is well-known to target many sites in histones, and that other sites are more robust than H3K27 (Choudhary's lab). Thus, given the evolutionary conservation, the results of this study establish a foundation in H3 lysine analysis to build upon to reveal roles of individual and redundant histone sites.

We thank the reviewer for the positive and constructive feedback and for recognising the importance of our work. Below, we provide detailed responses to the points raised, which have helped us to significantly strengthen the manuscript.

2. Fig 2: The authors make the reasonable assertion that failure to generate complete K14R and K18R clones, while K-to-K clones are generated efficiently, indicates that there is a fitness penalty to mutating those lysines. However, loss of fitness upon H3K14 and H3K18 mutation was not specifically demonstrated. To confirm essentiality in mESCs, editing should be done in the presence of a recombinant H3 engineered to harbour mutations to protect it from mutagenesis. After editing is complete, the recombinant H3 can be excised using a FLP, LoxP, or similar system, to then experimentally demonstrate loss of fitness. In short, incomplete K-to-R editing is used as a proxy for loss of cellular fitness throughout and is a basis for the title of the manuscript, so it is essential to confirm this.

In addition to our inability to generate full K-to-R mutants for H3K14 and H3K18 (despite readily deriving the corresponding K-to-K mutants), we obtained several independent lines of evidence indicating that this difference reflects a biological effect rather than a technical limitation. First, detailed

genotyping of partially edited clones revealed distinct edited alleles across independent lines (**Extended Data Fig.3f,g**), arguing against the possibility that specific alleles are intrinsically resistant to mutagenesis. Second, additional rounds of editing in partial H3K14R and H3K18R mutants failed to increase the number of mutant alleles (**Extended Data Fig.3b,c**). Finally, partial H3K14R and H3K18R mutants exhibited pronounced stem self-renewal defects in colony-formation assays (**Fig.2g,h**), demonstrating a substantial negative impact on cellular fitness even when these mutations are present in only a subset of alleles.

While exogenous complementation followed by conditional cassette excision could, in principle, provide an inducible strategy to directly test the contribution of H3K14 and H3K18 to mESC proliferation, such an approach is fundamentally confounded by the complex regulation that enforces strict histone dosage and expression timing. Canonical histones H3.1 and H3.2 are expressed exclusively during S phase to meet the tightly regulated demand for chromatin assembly during DNA replication. Their genes are organised into genomic clusters, and expression is coordinated through specialised nuclear compartments (histone locus bodies), cell-cycle-dependent activation by specific transcription factors, and unique mRNA processing mechanisms[1]. These layers of regulation are essential to maintain a strict stoichiometric balance between histone supply and demand. Consistent with this, even modest (2–3-fold) ectopic doxycycline-inducible overexpression of canonical H3 and H4 histones has been shown to induce S/G2 cell-cycle arrest in human cells[2,3].

To explore whether an exogenous histone supply could be rendered responsive to endogenous histone gene regulatory mechanisms, and, thereby, enable a complementation assay without substantially perturbing histone dosage, we undertook the following approach:

a) We identified histone H3 genes expressed at comparatively high levels in mESCs using available CAGE-sequencing data (https://fantom.gsc.riken.jp/5/): *Hist1h3a, Hist1h3b, Hist1h3c, Hist1h3e, Hist1h3g, Hist1h3i.*

b) We defined their putative cis-regulatory elements (CREs) based on ENCODE datasets across multiple cell types. **Rebuttal Fig.1** shows an example genomic region selected for exogenous expression (*Hist1h3e+CREs*):

Rebuttal Fig.1. *Hist1h3e* gene with the surrounding CREs selected for cloning

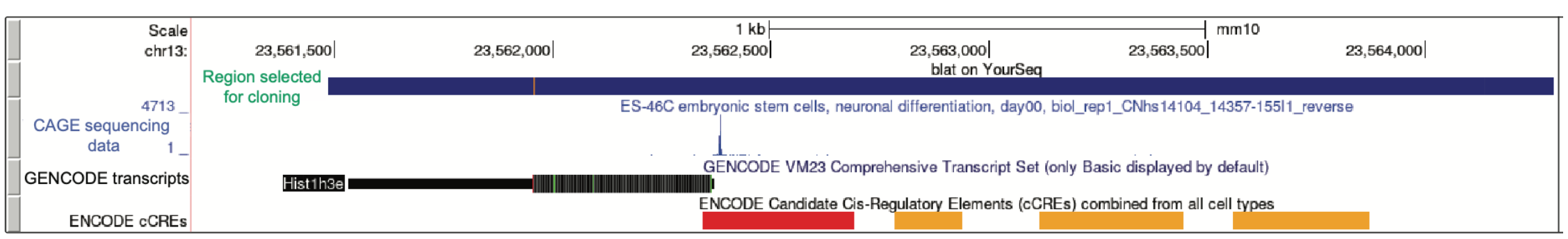

c) We generated two piggyBac (PB) vectors containing three histone H3 genes plus their associated CREs arranged back-to-back and flanked by loxP sites (**Rebuttal Fig.2**). We have added an HA tag to one of the histone genes (*Hist1h3e*) to allow monitoring construct expression. No exogenous promoters or other regulatory elements were included beyond the native histone gene CREs.

Rebuttal Fig.2. PB-b-e(HA)-i and PB-g-a-c constructs

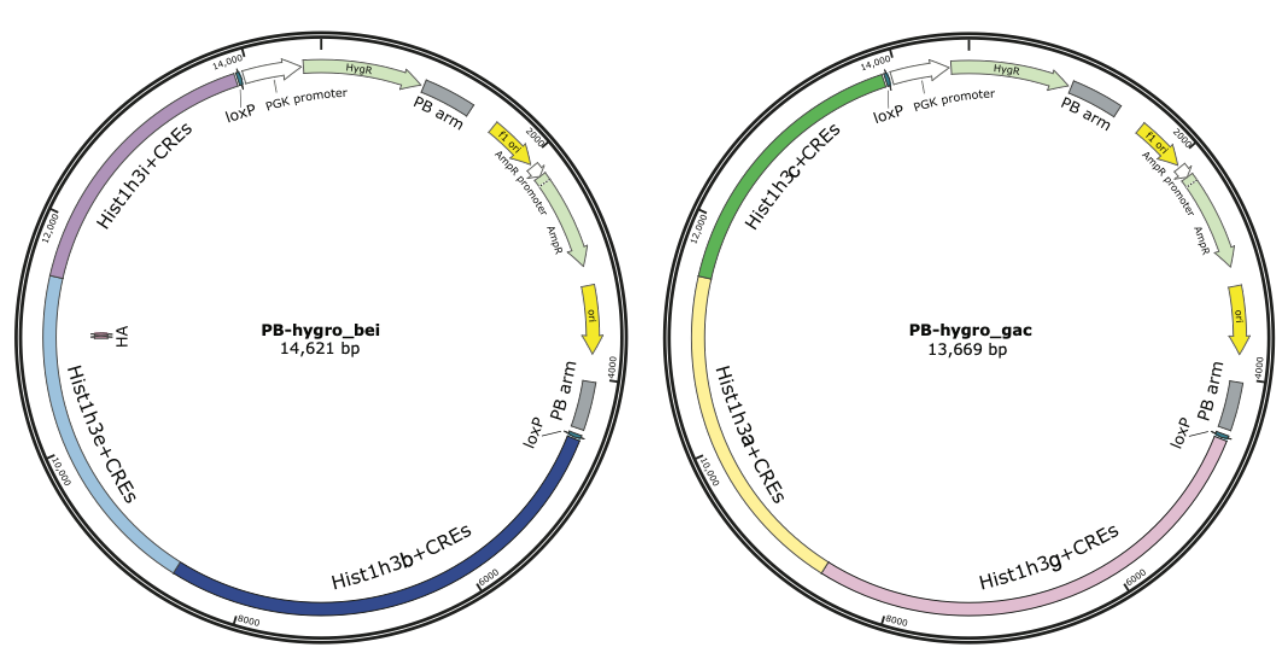

Next, these piggyBac constructs were introduced into H3K23R mutant cells under conditions promoting multiple genomic integrations, using two different transposase (PBase)-transposon (PB) ratios. This strategy allowed us to assess the functional contribution of the exogenous histone pool by monitoring potential restoration of H3K23ac levels. On average, we achieved 6-8 integrations per cell, corresponding to approximately 18–24 additional histone H3 gene copies (**Rebuttal Fig.3a**). Although the exogenously supplied histones were detectably expressed, as assessed by anti-HA and anti-H3 Western blot, this did not result in detectable restoration of the H3K23ac signal (**Rebuttal Fig.3b**). This limited rescue suggests that faithful recapitulation of endogenous histone gene regulation likely requires a more comprehensive set of regulatory elements than those included here. Systematic dissection of these regulatory requirements would represent a substantial independent line of investigation and would detract from the primary focus of this study on prime-editing-based histone gene mutagenesis.

Rebuttal Fig.3. Contribution of exogenous histone H3 expressed under native regulatory elements

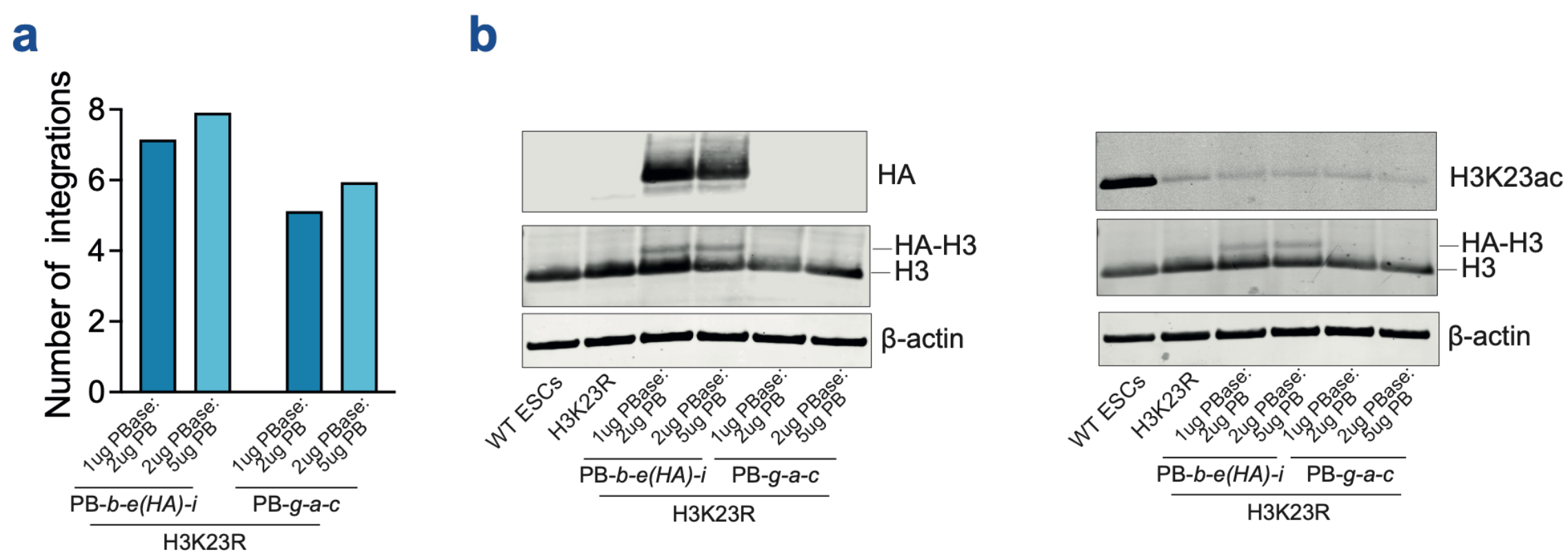

In the revised manuscript, we have, therefore, improved the text throughout to ensure we do not claim essentiality of the sites for which we did not achieve full editing, but instead use wording like "impair cellular fitness" and "impact mESC proliferation".

3. Fig 4g: ChIP-seq would be informative, to understand whether acetylation is being lost in the H3.1/2/3 clones at particular loci, whether H3.3 is more frequently acetylated and/or redistributed in the H3.1/2 clones, and to understand how histone variants might be compensating for each other as suggested in the text lines 269-270.

To address this question, we performed CUT&Tag-based genomic profiling of H3.3 incorporation and H3K18ac levels in wild-type (H3.1/2K18K), H3.1/2K18R, and H3.1/2/3K18R cells. Across H3K18ac peaks, we observed a pronounced redistribution of the H3K18ac signal in H3.1/2K18R cells, with some loci showing reduced and others increased signal (**Fig.4h**). Notably, regions exhibiting increased H3K18ac strongly correlated with H3.3 localisation, while depletion of H3K18ac was largely confined to H3.3-poor loci. Introduction of the H3.3K18R mutation to partial H3.1/2K18R abolished this redistribution and resulted in an overall mild reduction of H3K18ac levels (**Fig.4h**). To further examine potential compensation of the H3K18ac by H3.3, we focused on strong enhancer and promoter regions, which are generally characterised by high H3.3 levels[4–6]. Strikingly, at both classes of regulatory elements, increased H3K18ac in H3.1/2K18R mutant was accompanied by increased H3.3 incorporation, an effect that was lost upon further addition of the H3.3K18R mutation (**Fig.4i-k**). Finally, comparison of H3.1/2-to-H3.3 ratios in wild-type and H3.1/2K18R cells by mass spectrometry showed increased H3.3 chromatin incorporation in the H3.1/2K18R cells, providing independent evidence for histone variant compensation (**Fig.4l**).

4. Fig 5c (& e): Since the data is not expected to be normally distributed, t-test is inappropriate. The data show increased copy number loss in the -nickRNA K18R column, from 25% to 71%, in contrast to the assertion in line 292 that there is little impact, and lines 311 and 434, that omitting nickRNA will minimize large deletions. The authors should reconcile their conclusions with the K18R copy number

data in 5c. Or, perform additional replicates, if the data in 5c is believed to have the same distribution as 'K18 K-to-R' in Fig 5e.

We agree with the reviewer that analysing a relatively small number of clones (6–12 per condition) and applying a *t*-test contributed to inconsistencies in the interpretation of histone cluster copy number loss in **Fig.5c** and **Fig.5e**. To generate more robust data, we repeated these experiments with an increased number of independent clones (23–31 per site) and expanded the analysis to include an additional locus, H3K14, resulting in a total of three sites examined.

Copy number estimates obtained by qPCR were categorised as 1 copy (<1.5), 2 copies (≥1.5 and <2.5), or 3 copies (≥2.5). Differences between groups were then assessed using Fisher's exact test, followed by Benjamini–Hochberg correction for multiple comparisons. Using this approach, we found that removal of the nickRNA reduced the frequency of copy number loss for H3K23R, but not for H3K14R or H3K18R (**Fig.5c**), indicating that the effect of adding nickRNA is locus-dependent. This conclusion is further supported by shallow whole-genome sequencing of 10 clones, which revealed kilobase-scale deletions in histone clusters only in the H3K23R+nickRNA condition (**Extended Data Fig.4d-g**), consistent with the qPCR-based categorical analysis. Accordingly, we have revised the conclusions in lines 321, 339 and 343 to reflect this locus-specific effect.

For **Fig.5e**, we similarly increased the number of analysed clones to at least 20 per condition and included the additional H3K14 site to improve statistical robustness. We then applied the same analytical framework used for **Fig.5c**, i.e., categorisation of copy number values followed by Fisher's exact test with Benjamini–Hochberg correction. This revealed no differences in the frequency of histone cluster 1 loss between the K-to-K and K-to-R epegRNA conditions.

5. Fig 5: Is there a correlation between % editing (such as in Fig 1i) and the frequency of large or small deletions? Would they be defined as indels?

Standard Cas9 nuclease-based CRISPR gene editing is known to generate two types of genomic by-products: small insertions or deletions (<20 bps), commonly called **indels**, and larger kilobase- to megabase-scale deletions[7,8]. Only the former are typically defined as indels. While the frequency of both classes of by-products is markedly reduced in CRISPR prime editing, they still occur at low frequency[8,9].

Small indels are captured by our amplicon-based NGS analysis and reported throughout the manuscript across all editing conditions. Indel frequency did not meaningfully correlate with editing efficiency (**Extended Data Fig.5c,d**).

Kilobase- and megabase-scale deletions cannot be detected by amplicon sequencing and require whole-genome sequencing for comprehensive quantification. We performed whole-genome sequencing on a subset of highly edited H3K18R and H3K23R clones (**Extended Data Fig.4d-g**). Because these clones were pre-selected based on high editing efficiency, these data are not suitable for unbiased correlation analysis between the frequency of large deletions and editing efficiency. To address this limitation, we used the histone cluster 1 copy number qPCR assay developed in this study (**Fig.5c,e**) as a proxy for large-scale deletions and assessed its relationship with editing efficiency across a broader set of clones (n = 139). Using this approach, we found no strong correlation between editing efficiency and the frequency of histone cluster 1 copy number loss (**Extended Data Fig.4b,c**).

Together, these results suggest that the occurrence of both small indels and large deletions reflects rare, stochastic DNA double-strand break events rather than increased prime editing activity.

We have added the above analysis and its discussion to the revised manuscript (**Extended Data Fig.5c,d** and **Extended Data Fig.4b,c**, lines 389 and 324).

6. Fig 6: Is there a correlation between how essential each lysine is with frequency that they are mutated in developmental disease or cancer, given the extensive sequence information available for histone mutations?

To address this, we leveraged the recent comprehensive analysis of somatic histone mutations and oncogenic driver prediction in approximately 12,000 adult tumour samples[10]. This study identified driver mutations at H3K4, H3K14, H3K18, H3K27, H3K36, and H3K64 (**Extended Data Fig.6c**). The frequency and predicted driver status of the mutations did not correlate with the loss-of-fitness phenotypes we observed. Notably, the driving status of the acetyl-mimic H3K18Q mutation reinforces the functional relevance of acetylation at this residue. This information has been included in the revised manuscript (lines 497-504).

7. Fig 6: Conclusions of the study, especially in light of previous results mutating the single H3 K27, to examine whether simultaneous loss of two non-essential lysines lead to fitness deficits, and in particular, of K27. This will provide important information about functional redundancy of histone modifications.

To investigate whether simultaneous loss of multiple lysines can uncover functional redundancy, we systematically examined the effects of combinatorial substitutions. We focused on four lysines for which highly edited mutant lines were generated and for which robust antibodies were available (H3K23, H3K27, H3K36, and H3K37). Using these single mutant lines, we introduced all possible double-mutant combinations to assess whether the combined loss of individually non-essential residues can be achieved.

Highly edited clones were obtained for all attempted combinations (**Fig.8a**) and showed loss of the corresponding histone modifications (**Fig.8b-d, Extended Data Fig.7a,b**). There was no statistically significant difference between the K-to-K and K-to-R editing during the second site mutagenesis (**Fig.8a**). For the K27+K36 combination, we generated mutants in both sequential orders (K27→K36 and K36→K27), confirming that the editing efficiency of the second site was not affected by prior lysine substitution. The K36+K37 pair was not attempted due to a shared PAM site that becomes disrupted during the first editing round.

Since our fitness assay primarily measures overt proliferation defects under routine culture conditions, we next assessed <u>stem cell self-renewal capacity</u> under clonal conditions for selected single and double mutants involving H3K27 (H3K27R, H3K36R, H3K37R, H3K27R+H3K36R, and H3K27R+H3K37R). When plated at clonal density, double-mutant cells exhibited a marked reduction in alkaline-phosphatase-positive colony-forming capacity compared with the corresponding single mutants, with the strongest impairment observed in H3K27R+H3K36R cells (**Fig.8e**).

To further characterise the molecular basis of the H3K27R+H3K36R phenotype, we performed RNA-sequencing of wild-type, single- (H3K27R, H3K36R), and double-mutant (H3K27R+H3K36R) cells (**Fig.8f**). Differential expression analysis using DESeq2[11] with a factorial design was used to model genetic interactions between the two mutations (**Fig.8g**). Clustering of genes with strong interaction effects revealed several distinct transcriptional patterns. The majority of genes exhibited epistatic transcriptional responses, with the double mutant resembling either the H3K27R (clusters 4, 5 and 7; 2756 genes) or H3K36R (clusters 2 and 9; 1667 genes) single mutant. In addition, one cluster of genes (cluster 1; 419 genes) showed a buffering interaction, characterised by increased expression in both single mutants, but a partial reversion towards wild-type levels in the double mutant. Finally, three gene subsets (clusters 3, 6, and 8; 1125 genes) exhibited emergent transcriptional responses, defined by expression changes in the double mutant that were distinct from both single mutants and wild type. Genes within the emergent clusters were enriched for pathways related to cell differentiation, proliferation, and transcriptional regulation (**Extended Data Fig.7c**). These included downregulated pluripotency-maintaining factors such as *Klf4*[12,13], *Chd4*[14], and *Sin3a*[15], and upregulated differentiation drivers including *Fgfr2*[16] and *Dkk*[17], suggesting that their dysregulation may underlie the reduced stem self-renewal capacity in the double-mutant cells.

Together, these results demonstrate that the combined loss of specific lysine residues could reveal functional redundancy and crosstalk between histone modifications that is not apparent from single-site perturbations alone.

8. Line 382: Was a standard curve included for the Western blots? If not, should rewrite to “3-4-fold decrease in Western blot signal”, or similar.

We did not include a standard curve for the Western blots. Accordingly, we have revised the wording throughout the manuscript to avoid absolute quantification and now refer to changes as fold differences in Western blot signal rather than modification abundance. We have also added a description of the quantification procedure (fluorescence intensity measurements normalised to total H3 loading control) to the Materials and Methods.

In addition, based on feedback from Reviewer #3, we repeated all Western blots three times independently using separately collected cell pellets and now present the corresponding blots, quantification and statistics alongside each blot.

9.Line 447: Suggestions for mitigating this suboptimal outcome, or predicting whether/why a locus would be impacted?

To investigate factors contributing to locus-specific differences in PAM-only editing frequency, we analysed several parameters, including epegRNA extension length, primer binding site (PBS) length, reverse transcription template (RTT) length, PBS GC%, PBS melting temperature (Tm), predicted on-target score, PAM codon identity and usage frequency in mammals, PAM position in the RTT, and target edit position in the RTT. Among these, only PBS Tm showed a moderate-to-strong correlation with PAM-only frequency (**Extended Data Fig.5e**). Higher PBS Tm may increase the frequency of reverse transcription initiation, which, particularly under conditions of MMR inhibition, may result in more incomplete or abortive prime editing products and consequently higher levels of PAM-only mutations. Differences in intramolecular folding of the epegRNAs and sequence or chromatin environment may additionally contribute to this locus-specific variation. This correlation analysis and its discussion have been incorporated into the revised manuscript (lines 390-401, **Extended Data Fig.5e**).

Reviewer #2 (Remarks to the Author):

This manuscript reports the use of a CRISPR prime editing-based approach for histone gene mutagenesis. While technically sound, the study is primarily methodological in nature and lacks novel biological insights.

In response to the reviewer's feedback, we have substantially strengthened the manuscript both technically and conceptually. We have incorporated genome-wide CUT&RUN/CUT&Tag profiling, mass spectrometry validation, and quantitative replication of all Western blot analyses to ensure robust and well-supported conclusions. Moreover, we have further expanded the biological insights of the study. In the original submission, we demonstrated that specific H3 lysines differentially contribute to cellular fitness. We also showed conservation of H3K56ac function in the mammalian DNA damage response. In the revised manuscript, we uncover locus-specific redistribution of H3K18ac and reveal H3.3-mediated compensation for this mark in the partial H3.1/2K18R mutants, highlighting the power of our system for dissecting variant-specific functions of histone modifications. Furthermore, by generating five novel double histone mutants, we reveal new phenotypic and transcriptional relationships emerging from systematic combinatorial histone mutagenesis.

A detailed point-by-point response can be found below.

1. All histone modification assessments were performed by Western blot analysis, which is not sufficiently quantitative; more robust techniques such as mass spectrometric methods and/or ChIP-seq are needed to accurately measure modification levels and loci specificity.

To address this concern, we have complemented our Western blot analyses with genome-wide profiling and mass spectrometry-based validation. We have also improved the robustness of the Western blots.

a) To assess genome-wide changes of specific histone modifications in highly edited clones, we performed profiling of H3K23ac, H3K27me3 and H3K36me3 in H3K23R, H3K27R and H3K36R clones, respectively, using CUT&RUN or CUT&Tag. Consistent with the Western blot results, this revealed a global loss of all three modifications across the genome in the R mutant cells (**Fig.7e,f**). In addition, CUT&Tag profiling of H3K18ac and H3.3 variant in partial H3K18R mutants showed a pronounced redistribution of the H3K18ac signal in H3.1/2K18R cells, with some loci showing reduced and others increased signal (**Fig.4h**). Notably, regions exhibiting increased H3K18ac strongly correlated with H3.3 localisation, while depletion of H3K18ac was largely confined to H3.3-poor loci. Increased H3K18ac in H3.1/2K18R mutant was accompanied by increased H3.3 incorporation, an effect that was lost upon further addition of the H3.3K18R mutation (**Fig.4i-k**). Finally, comparison of H3.1/2-to-H3.3 ratios in wild-type and H3.1/2K18R cells by mass spectrometry showed increased H3.3 chromatin incorporation in the H3.1/2K18R cells, providing independent evidence for histone variant compensation (**Fig.4l**).

b) We have validated the successful mutagenesis of K23R, K14R and K18R mutants using mass spectrometry (**Extended Data Fig.2c, Extended Data Fig.3d.e**).

c) We repeated all Western blots three times independently using separately collected cell pellets and now present the corresponding quantification and statistics alongside each blot.

2. Moreover, the editing efficiency varies significantly across different lysine residues (e.g., H3K4), making it difficult to draw meaningful conclusions about correlations with cellular fitness.

In our system, the overall efficiency and reproducibility of histone H3 editing are high and sufficient to support robust fitness analyses. For 12 of the 13 histone H3 lysine residues examined, synonymous K-to-K editing achieved ≥86% conversion after only two rounds of transient pegRNA transfection, corresponding to modification of at least 21 out of 24 histone H3 alleles. This high and reproducible baseline editing efficiency provides adequate statistical power to detect loss-of-fitness effects associated with K-to-R substitutions.

In the initial submission, H3K4 represented the only locus with substantially lower editing efficiency, with K-to-K conversion reaching ~33%. In the revised manuscript, we implemented two complementary strategies to improve editing at this site (lines 412-437), increasing K-to-K efficiency to 51%. In contrast, K-to-R editing at H3K4 remained extremely rare, with only 2 out of 36 clones showing low-level mutation (11–12%, **Extended Data Fig.5j-m**). The reproducible and statistically significant disparity between K-to-K and K-to-R editing at this locus strongly suggests negative selection against H3K4R-edited cells rather than a technical limitation of the editing approach, supporting the conclusion that mutation of this residue imposes a fitness cost.

All editing experiments in the manuscript were performed using three or more biological replicates (independent cell transfections), with derivation and analysis of at least 47 independent clonal lines per condition. Statistical analyses were conducted using conservative tests with correction for multiple comparisons throughout. Together, these features provide a robust framework for assessing the relationship between editing outcomes and cellular fitness, despite some locus-specific differences in raw editing efficiency. **Importantly, our conclusions are based on within-locus comparisons between K-to-K and K-to-R editing rather than on absolute editing efficiencies across different lysine residues.** Details of biological replicates and statistical tests for all figure panels are now provided in **Supplementary Table 3**.

3. Additionally, single-site lysine-to-arginine (K→R) mutations may have limited interpretability due to compensatory effects, histone mark crosstalk, and the presence of other modifications (e.g., methylation or acetylation) on the same residue.

To investigate whether simultaneous loss of multiple lysines can uncover functional redundancy, we systematically examined the effects of combinatorial substitutions. We focused on four lysines for which highly edited mutant lines were generated and for which robust antibodies were available (H3K23, H3K27, H3K36, and H3K37). Using these single mutant lines, we introduced all possible double-mutant combinations to assess whether the combined loss of individually non-essential residues can be achieved.

Highly edited clones were obtained for all attempted combinations (**Fig.8a**) and showed loss of the corresponding histone modifications (**Fig.8b-d, Extended Data Fig.7a,b**). There was no statistically significant difference between the K-to-K and K-to-R editing during the second site mutagenesis (**Fig.8a**). For the K27+K36 combination, we generated mutants in both sequential orders (K27→K36 and K36→K27), confirming that the editing efficiency of the second site was not affected by prior lysine substitution. The K36+K37 pair was not attempted due to a shared PAM site that becomes disrupted during the first editing round.

Since our fitness assay primarily measures overt proliferation defects under routine culture conditions, we next assessed <u>stem cell self-renewal capacity</u> under clonal conditions for selected single and double mutants involving H3K27 (H3K27R, H3K36R, H3K37R, H3K27R+H3K36R, and H3K27R+H3K37R). When plated at clonal density, double-mutant cells exhibited a marked reduction in alkaline-phosphatase-positive colony-forming capacity compared with the corresponding single mutants, with the strongest impairment observed in H3K27R+H3K36R cells (**Fig.8e**).

To further characterise the molecular basis of the H3K27R+H3K36R phenotype, we performed RNA-sequencing of wild-type, single- (H3K27R, H3K36R), and double-mutant (H3K27R+H3K36R) cells (**Fig.8f**). Differential expression analysis using DESeq2[11] with a factorial design was used to model genetic interactions between the two mutations (**Fig.8g**). Clustering of genes with strong interaction effects revealed several distinct transcriptional patterns. The majority of genes exhibited epistatic transcriptional responses, with the double mutant resembling either the H3K27R (clusters 4, 5 and 7; 2756 genes) or H3K36R (clusters 2 and 9; 1667 genes) single mutant. In addition, one cluster of genes (cluster 1; 419 genes) showed a buffering interaction, characterised by increased expression in both single mutants, but a partial reversion towards wild-type levels in the double mutant. Finally, three gene subsets (clusters 3, 6, and 8; 1125 genes) exhibited emergent transcriptional responses, defined by

expression changes in the double mutant that were distinct from both single mutants and wild type. Genes within the emergent clusters were enriched for pathways related to cell differentiation, proliferation, and transcriptional regulation (**Extended Data Fig.7c**). These included downregulated pluripotency-maintaining factors such as *Klf4*[12,13], *Chd4*[14], and *Sin3a*[15], and upregulated differentiation drivers including *Fgfr2*[16] and *Dkk*[17], suggesting that their dysregulation may underlie the reduced stem self-renewal capacity in the double-mutant cells.

Together, these results demonstrate that the combined loss of specific lysine residues could reveal functional redundancy and crosstalk between histone modifications that is not apparent from single-site perturbations alone.

More broadly, while we generated five combinatorial mutant lines in this study, the prime editing framework we established is readily extendable and enables systematic exploration of additional combinations, including the incorporation of other histone genes and variants. We are confident that this platform will facilitate broader dissection of histone modification crosstalk in future studies.

Finally, we acknowledge that lysine-to-arginine mutagenesis cannot distinguish between the loss of different post-translational modifications (e.g., acetylation versus methylation) at a given residue. This is an inherent limitation of histone mutagenesis approaches and was explicitly discussed in the Discussion of the initial submission (lines 685-690). Importantly, this limitation is shared by all genetic strategies that substitute individual residues in proteins and does not detract from the conclusions drawn in this study.

4. Incomplete editing across histone variants (H3.1, H3.2, and H3.3) further complicates interpretation.

All pegRNAs targeting canonical histone H3 were designed to simultaneously edit all H3.1 and H3.2 genes, which are highly conserved at the nucleotide level (representative alignments in **Fig.1a; Fig.2a,b**). Consequently, editing efficiencies across canonical H3 variants are equivalent, and no variant-specific incompleteness arises within the H3.1/2 group (representative examples in **Extended Data Fig.2; Extended Data Fig.3f,g**).

In contrast, canonical (H3.1/2) and non-canonical (H3.3) histones are substantially divergent in sequence, necessitating separate targeting strategies. Because H3.3 is encoded by only two genes, it can be readily edited independently, and we demonstrate efficient editing of H3.3 at multiple lysine residues (H3.3K23R and H3.3K18R; **Fig.4a-f**). Importantly, canonical and non-canonical histone H3 variants differ in expression dynamics, chaperone usage, chromatin incorporation, and biological function[18]. Separate targeting of H3.1/2 and H3.3, therefore, represents a feature rather than a limitation of our approach, as it enables variant-resolved dissection of histone modification function.

Consistent with this, by generating H3.1/2K18R mutants alongside combined H3.1/2/3K18R mutants, we were able to reveal the compensatory contribution of H3.3 to the maintenance of H3K18ac when canonical H3 variants are partially mutated (lines 284-298, **Fig.4d-l**). These findings underscore the importance of variant-specific editing for accurate functional interpretation.

5. Overall, given the lack of new biological discoveries and the methodological limitations, the manuscript is not suitable for publication in Nature Genetics.

In summary, the revised manuscript integrates orthogonal quantitative approaches with systematic combinatorial genetics, substantially strengthening both methodological rigour and biological insight. We believe the additional data address the reviewers' concerns and clarify the mechanistic and conceptual advances of the study.

Reviewer #3 (Remarks to the Author):

General summary and opinion

In the present study, Price et al. present a high-throughput CRISPR prime editing platform that enables the precise, base-resolution mutation of lysine residues across all canonical and variant histone H3 genes in mouse embryonic stem cells. Using this system, the authors introduce lysine-to-arginine substitutions, paired with silent lysine-to-lysine controls, to dissect the contribution of individual lysine residues to cellular fitness and chromatin regulation. They systematically map post-translational modification outcomes and fitness consequences across a comprehensive series of histone mutants.

This work show that the K-R mutation at several lysines (H3K4, H3K9, H3K14, H3K18, and H3K79) result in viability defects, consistent with known essential roles for their corresponding PTMs. In contrast, other residues, including H3K23, H3K36, H3K56, and H3K122, appear to be dispensable in self-renewing mESCs. They validate editing efficiency, monitor loss of specific marks, and relate some of these perturbations to cell growth phenotypes. Additional mechanistic depth is explored via chromatin mark recovery.

This study builds on prior approaches in yeast and Drosophila and more recent histone knockout systems, but offers technical advance: the ability to study individual histone lysines in their native genomic context, without reliance on transgenes or exogenous histone supply. The methodological execution is careful, and the platform itself is clearly powerful, both scalable and adaptable for future applications such as modeling disease-associated oncohistones or interrogating PTM crosstalk.

However, while the technical achievements are significant, the biological insight drawn from these experiments is at times underdeveloped. Several findings reinforce existing knowledge rather than breaking conceptual ground, and key results, such as the failure to efficiently mutate H3K4, are insufficiently addressed. Furthermore, figure presentation is inconsistent, with a lack of quantification across key western blots and missing statistical annotation for multiple panels. Additionally, the discussion does not fully explore developmental implications of histone lysine function in mESCs, or the potential biological depth.

While this work could serve as a foundation for future efforts to map and manipulate the histone code in mammalian systems, the manuscript would benefit from clearer data presentation, a more expanded discussion of key mechanistic insights, and clarification of a few experimental choices. Below, we outline a series of major and minor points intended to help strengthen the impact and clarity of the study.

We thank the reviewer for the constructive and detailed feedback, which helped us improve both the clarity and biological depth of the manuscript. Our point-by-point responses are detailed below.

Major concerns

(1) Lack of functional combinatorial analysis

The authors provide a demonstration of combinatorial editing capability by designing a single epegRNA to simultaneously target H3K14, H3K18, and H3K23 (Fig. 3e-g). This is an important proof-of-principle, showing that the platform can introduce multiple lysine mutations in cis within a single histone tail using prime editing. The successful editing of the triple K-K and partial editing of the triple K-R construct illustrate the feasibility of multi-site editing, even within a short target window. However, this experiment remains a demonstration of editing feasibility, rather than a functional analysis of combinatorial histone modification since (1) the triple mutant is assessed only at the DNA level in pooled cells, without clonal isolation, western blot validation, or phenotypic characterization and (2) since K14R and K18R are independently deleterious, the strong reduction in triple K-R reads is expected and does not allow for functional dissection of crosstalk or synthetic interactions. Therefore, we suggest including a designed double/triple mutant involving at least one tolerated residue (for

example K27R+K36R or even K27R+K36R+K37R), with some degree of phenotypic or biochemical readout (histone PTM levels, growth) to proof the combinatorial analysis system is functionally flexible.

To investigate whether simultaneous loss of multiple lysines can uncover functional redundancy, we systematically examined the effects of combinatorial lysine substitutions. We focused on four lysines for which highly edited mutant lines were generated in the initial version of the manuscript and for which robust antibodies were available (H3K23, H3K27, H3K36, and H3K37). We then endeavoured two independent strategies: a) *sequential mutagenesis* (adding a second mutation in the pre-established single mutant clones); and b) *simultaneous mutagenesis* to achieve multiple mutations with a single pegRNA.

a) Sequential mutagenesis

Using single mutant lines, we introduced all possible double-mutant combinations to assess whether the combined loss of individually non-essential residues can be achieved. Highly edited clones were obtained for all attempted combinations (**Fig.8a**) and showed loss of the corresponding histone modifications (**Fig.8a-c, Extended Data Fig.7a,b**). There was no statistically significant difference between the K-to-K and K-to-R editing during the second site mutagenesis (**Fig.8a**). For the K27+K36 combination, we generated mutants in both sequential orders (K27→K36 and K36→K27), confirming that the editing efficiency of the second site was not affected by prior lysine substitution. The K36+K37 pair was not attempted due to a shared PAM site that becomes disrupted during the first editing round.

Since our fitness assay primarily measures overt proliferation defects under routine culture conditions, we next assessed stem cell self-renewal capacity under clonal conditions for selected single and double mutants (H3K27R, H3K36R, H3K37R, H3K27R+H3K36R, and H3K27R+H3K37R). When plated at clonal density, double-mutant cells exhibited a marked reduction in alkaline-phosphatase-positive colony-forming capacity compared with the corresponding single mutants, with the strongest impairment observed in H3K27R+H3K36R cells (**Fig.8e**).

To further characterise the molecular basis of the H3K27R+H3K36R phenotype, we performed RNA-sequencing of wild-type, single- (H3K27R, H3K36R), and double-mutant (H3K27R+H3K36R) cells (**Fig.8f**). Differential expression analysis using DESeq2[11] with a factorial design was used to model genetic interactions between the two mutations (**Fig.8g**). Clustering of genes with strong interaction effects revealed several distinct transcriptional patterns. The majority of genes exhibited epistatic transcriptional responses, with the double mutant resembling either the H3K27R (clusters 4, 5 and 7; 2756 genes) or H3K36R (clusters 2 and 9; 1667 genes) single mutant. In addition, one cluster of genes (cluster 1; 419 genes) showed a buffering interaction, characterised by increased expression in both single mutants, but a partial reversion towards wild-type levels in the double mutant. Finally, three gene subsets (clusters 3, 6, and 8; 1125 genes) exhibited emergent transcriptional responses, defined by expression changes in the double mutant that were distinct from both single mutants and wild type. Genes within the emergent clusters were enriched for pathways related to cell differentiation, proliferation, and transcriptional regulation (**Extended Data Fig.7c**). These included downregulated pluripotency-maintaining factors such as *Klf4*[12,13], *Chd4*[14], and *Sin3a*[15], and upregulated differentiation drivers including *Fgfr2*[16] and *Dkk*[17], suggesting that their dysregulation may underlie the reduced stem self-renewal capacity in the double-mutant cells.

Together, these results demonstrate that the combined loss of specific lysine residues could reveal functional redundancy and crosstalk between histone modifications that is not apparent from single-site perturbations alone.

b) Simultaneous mutagenesis

To assess whether multi-site histone mutants can be generated using a single pegRNA encoding multiple substitutions, we designed a series of combinatorial pegRNAs spanning different editing window lengths: K36R+K37R (4 bp), K23R+K27R (13 bp), and K27R+K36R (28 bp) (**Extended Data Fig.7d**).

In addition, we generated a pegRNA encoding substitutions at four sites simultaneously (K23R+K27R+K36R+K37R; 43 bp editing window).

Cells were subjected to four rounds of transient pegRNA transfection, after which individual clones were isolated and genotyped by amplicon-based NGS. Editing efficiency showed a strong inverse relationship with the distance between target mutations (**Extended Data Fig.7e-g**): the shortest editing window (K36R+K37R) yielded the highest efficiency (up to 46%), whereas the longest double-mutant configuration (K27R+K36R) resulted only in singly edited clones. For the quadruple-target pegRNA, multiple clones carrying triple mutations were recovered (efficiency up to 24%); however, no quadruple mutants were obtained (**Extended Data Fig.7h**).

In summary, simultaneous prime editing of multiple histone sites using a single pegRNA is feasible but strongly constrained by editing window length and remains substantially less efficient than sequential mutagenesis strategies.

Notably, despite the relatively low efficiencies observed, our simultaneous targeting experiments extend the current scope of prime editing. To date, only two studies have reported simultaneous multi-site (> 2 substitutions) editing using a single pegRNA[19,20], achieving editing of two alleles within an editing window of up to 9 bps. In contrast, our approach targets 24 histone gene copies per cell with a longer editing window (31 bps in the case of triple mutants in **Extended Data Fig.7h**), representing a substantial increase in genomic and editing complexity and demonstrating the potential and current limitations of multiplex prime editing.

(2) Quantification of western blots and biological replicates

Most western blot gels (Figures 1j, 3d, 4c, 4g) are presented without quantification or mention of biological replicates. This limits interpretability, especially where the same histone mark in the same line is being compared across multiple panels or clones. In some cases, exposure differences lead to apparent inconsistencies (1j vs. 3d vs. 4c for H3K23ac).

- Please include normalization against loading control (using global anti-H3) across all WB panels.

In the revised manuscript, we have performed signal quantification and normalisation against the same loading control (total H3) for all the Western blots.

- Please include quantification (mean ± SD) from at least three biological replicates across all WB panels.

As a part of the revision process, we have performed and quantified all the Western blots in three independent biological replicates for all the manuscript panels (biological replicate = independently collected cell pellet and independently conducted Western blotting protocol). We have also provided all the uncropped Western blot images in **Supplementary Figures_uncropped WBs**.

- Clarify whether loading controls were consistent and whether the same gel and exposure conditions were used across panels.

All Western blots in this manuscript were performed using fluorescence-based detection on a LI-COR Odyssey scanner. For each blot, two primary antibodies from different species were used: one targeting the modification of interest and one targeting the loading control (total H3). Species-specific secondary antibodies conjugated to distinct fluorophores (700 nm and 800 nm) were imaged in separate channels. Depending on the species of the primary antibody used to detect the target modification (mouse or rabbit), we employed a corresponding total H3 loading control antibody from the opposite species. Catalogue numbers for all antibodies and the available validation information are listed in **Supplementary Table 2**.

All images were acquired using the **Auto** intensity setting, which automatically adjusts the detector gain to optimise signal within the linear dynamic range. Therefore, exposure settings are not reported, as signal intensity is determined by fluorophore properties and the scanner's automatic detector adjustment, not by manual exposure time.

After image acquisition, the Odyssey scanner software allows adjustment of brightness and contrast for display purposes, which may have created the impression of inconsistencies between several panels in the initial submission. Importantly, these adjustments do **not** alter the underlying pixel intensity values, which were used for fluorescence quantification. Quantification from the three biological replicates included in the revised manuscript was consistent across blots and was statistically compared across all the conditions.

(3) Interpretation of H3K4 mutants

Despite H3K4 being one of the most well-studied histone lysine residues, it is not represented in the main figures and receives only limited attention in the extended data figure 5, where the authors report low editing efficiencies for both the K-R mutation and the synonymous K-K control. The fact that the silent mutation results in unsuccessful editing may point to technical difficulties, such as pegRNA efficiency or sequence context, rather than selection against mutation. They mention this in the results section (lines 256-271). Nevertheless, in both abstract and discussion sections they repeatedly refers to H3K4 as one of the most deleterious or essential residues, along with well-substantiated cases like H3K18 or H3K79. These statements are difficult to reconcile with the limited data available.
Additionally, from the results showed in Extended figure 5g we can observe that there is at least one clone with ~20% H3K4R editing. We suggest this clone could be easily analysed for at least histone mark abundance to help clarify this issue.

To improve the interpretive consistency of the manuscript, we suggest the authors:
- Clarify their conclusions regarding the essentiality of H3K4.
- Discuss in more depth the unexpected outcome of the K-K editing attempt.
- Consider presenting any data from the partially edited clone, or comment on why further analysis was not pursued.

For H3K4, we performed the experiment using a pool of five epegRNAs, differing by one to two nucleotides in the protospacer and/or PBS region to account for sequence divergence among the twelve H3 genes at their 5′ end. Low prime-editing efficiency and the high rate of PAM-only or incomplete RT incorporation in this multiplex design may arise from several factors. First, epegRNAs with a protospacer perfectly matching one target gene can partially bind other H3 genes with incomplete matches, leading to transient R-loop formation, abortive nicking, or sequestration of PE–epegRNA complexes at nonproductive sites, preventing productive editing. Second, small sequence differences within the PBS across homologous loci can reduce PBS annealing efficiency, destabilise the extended 3′ flap, and result in incomplete reverse transcription incorporation, even when the spacer is perfectly matched. Third, delivering multiple epegRNAs simultaneously reduces the effective intracellular concentration of each guide, further limiting prime-editing efficiency. Together, these factors likely contributed to the low K-to-K editing efficiency observed in the initial screen.

To overcome these limitations, we implemented two strategies: a) removing the PAM mutation to prevent PAM-only edits that block further epegRNA binding, and b) extending the editing time window. For the latter, we either increased transient transfection rounds from 2 to 13 (**Extended Data Fig.5l**) or performed long-term editing via lentiviral delivery of epegRNA constructs, with analysis after six weeks of lower but constitutive epegRNA expression (**Extended Data Fig.5m**). These modifications allowed to increase H3K4 K-to-K editing, with the 13-round transfection raising median editing from 13.9% to 31% and maximum editing from 33% to 51% (**Extended Data Fig.5l**).

In contrast, K-to-R editing remained extremely rare, with only 2 out of 36 clones showing 11–12% mutation (**Extended Data Fig.5j,l**). The consistent and statistically significant difference between K-to-K and K-to-R editing indicates that the limited K-to-R mutagenesis reflects negative selection against

edited cells rather than technical failure, supporting the conclusion that this mutation confers fitness loss.

As suggested by the reviewer, we have performed Western blot analysis for H3K4me1 and H3K4me3 in the two partial H3K4R clones and observed a small decrease in H3K4me3 in the more edited mutant (**Extended Data Fig.5n**).

The revised manuscript now contains the new data described above, as well as a detailed discussion of the reasons for the low K-to-K editing efficiency of the H3K4 (lines 412-437).

(4) Deepening the biological context in the discussion

The discussion does not engage with the broader biological implications of the findings and is very results-oriented, particularly for residues described as non-essential based on mESC proliferation. Several lysines, including H3K36, K56, and K122, have established roles in transcriptional regulation, nucleosome dynamics, and genome stability. That mutations at these sites are tolerated under steady-state ESC culture is interesting, but it raises important questions about their potential roles in other contexts, such as during differentiation or in response to stress. Additionally, the observations on a relevant mutation like H3K27R aligns with previous data on PRC2 dispensability for self-renewal, but lacks deeper discussion on the role of this mark in proper lineage specification. A more focused discussion of these known biological functions, and how this system could be applied to tackle them in future work would strengthen the manuscript and better reflect the significance of the data.

We have substantially revised the Discussion to better contextualise our findings within known histone biology and to highlight how our system can be used to study context-dependent functions of histone modifications and their combinations beyond steady-state mESC proliferation.

Minor concerns

(1) Figure 1h/i/j: Please indicate in a different color in the lines from 1h that are analyzed in subsequent panels to help readers track clones across figures.

Throughout the manuscript, for all the editing experiments, we first pre-screened the clones using qPCR to estimate the ratio between the wild-type and edited H3 genes. We then selected the clones with the best editing ratios for subsequent NGS amplicon sequencing analysis. To better illustrate this key aspect of the experimental design, we arranged the clones based on the qPCR-estimated editing ratio in **Figure 1h** and highlighted with colour the top 11 clones taken for further analysis. To help readers track clones across figures, we also improved clone labelling in **Fig.1i,j, Fig.2, Fig.3c,d, Extended Data Fig.2a,b,** and **Extended Data Fig.3,b.** We have also updated the Methods section with the details on pre-screening of the clones by qPCR.

(2) In Figure 7e it is unclear that the mark measured is H3K18ac, clearly indicate.

We realised that panels 7e and 7f were inadvertently swapped in the initial submission, which has caused this confusion. We apologise for this error and have now corrected it. Revised **Fig.7h** shows a Western blot analysis of H3K18ac levels in TPMB cells treated with DMSO or A485 (5 or 10 µM) for 48 hours. **Fig.7i** shows the growth curve of TPMB cells treated under the same conditions for a total of 144 hours, with cell counts recorded every 48 hours.

(3) The figure legend for 7e says 48h treatment, but the x-axis extends to 144h. Clarify.

Please see the response to point 2 above, which clarifies this confusion.

(4) Extended Figure 6: The data on modifying enzymes are relevant. We suggest moving some of this content into the main figures.

We have moved the CRISPR-KO screen results on modifying enzymes to the main **Fig.7g**.

(5) Clone selection in figure 2 requires justification. Clone K18R_3 shows the highest editing efficiency (>40%) yet is not used in subsequent experiments. This raises the question of whether clone selection was biased or whether certain clones were excluded for non-obvious reasons.

Clone K18R_3 contains an indel in one of the histone H3 genes (**Fig.2d**, pink bar). For all follow-up experiments, we excluded clones harbouring indels, as these may produce histone copies with unknown or confounding biological properties.

Because our NGS amplicon sequencing covers 18 histone H3 alleles, an indel in a single gene would correspond to roughly 5% of reads for that clone. For clones with an indel frequency below 2%, we attribute detected indel events to PCR or sequencing errors, since the indel calculation spans the 80 bp window around the editing site. To make sure we clarify this important element of the clone selection process, we have introduced a sentence in the Methods section to describe this, which reads as follows:

"The prevalence of indels was determined by dividing the number of reads containing an insertion or deletion within an 80-bp window surrounding the cut site by the total number of aligned reads. Only clones with an indel rate below 2% were included in subsequent experiments, as such low levels were assumed to arise from PCR or sequencing errors rather than true genomic mutations. Because the amplicons encompass 18 histone H3 alleles, a true indel in a single allele would be expected to appear in approximately 5% of reads for that clone."

(6) H3.3 is central to the experimental strategy but is not introduced until late in the results section (lines 241-247). Please include a brief summary of its biological role earlier in the introduction to help contextualize why it's edited separately.

To improve clarity and provide appropriate context, we have moved the introduction of H3.3 to follow the description of the canonical mouse histone H3 genes. It now reads as follows:

"Murine canonical, or replication-dependent, histone H3 is encoded by nine H3.1 and three H3.2 genes, which collectively produce two proteins that differ by a single amino acid at position 96 (cysteine in H3.1 and serine in H3.2). H3.3 is a well-characterised non-canonical histone variant that is expressed throughout the cell cycle, replacing canonical histone H3 at actively transcribed loci and regions of heterochromatin, such as telomeres and pericentromeres[21]. Unlike replication-dependent H3 genes, H3.3 is encoded by only two genes (H3f3a and H3f3b) and is deposited by distinct chaperone complexes[22]."

(7) Rethink the use of the term "essential" and maybe temper to "strongly selected against" unless full lethality is shown.

As suggested, we have removed the word "essential" when referring to the loss-of-fitness effects conferred by specific mutations throughout the manuscript. We have used phrases "impair cellular fitness", "impact mESC proliferation" and "confer loss of fitness".

(8) The manuscript lacks clear and consistent reporting of statistical tests used and how error bars were calculated. Examples: Figures 3g, 7g.

We have provided a new **Supplementary Table 3**, which contains information on statistical analyses and the number and nature of biological replicates for each experiment. We have also included this information and how error bars were calculated in each corresponding figure legend.

(9) Data are claimed to be uploaded to SRA but accession number is missing.

SRA and GEO accession numbers are now provided in the Data Availability section of the manuscript.

(10) The suggestion that H3.3 compensates for mutated canonical histones in partially edited clones is plausible but speculative. We recommend briefly discussing this hypothesis in the discussion, noting its limitations and referencing available data on H3.3 genomic localization or dynamics.

To address this question, we performed CUT&Tag-based genomic profiling of H3.3 incorporation and H3K18ac levels in wild-type (H3.1/2K18K), H3.1/2K18R, and H3.1/2/3K18R cells. Across H3K18ac peaks, we observed a pronounced redistribution of the H3K18ac signal in H3.1/2K18R cells, with some loci showing reduced and others increased signal (**Fig.4h**). Notably, regions exhibiting increased H3K18ac strongly correlated with H3.3 localisation, while depletion of H3K18ac was largely confined to H3.3-poor loci. Introduction of the H3.3K18R mutation to partial H3.1/2K18R abolished this redistribution and resulted in an overall mild reduction of H3K18ac levels (**Fig.4h**). To further examine potential compensation of the H3K18ac by H3.3, we focused on strong enhancer and promoter regions, which are generally characterised by high H3.3 levels[4–6]. Strikingly, at both classes of regulatory elements, increased H3K18ac in H3.1/2K18R mutant was accompanied by increased H3.3 incorporation, an effect that was lost upon further addition of the H3.3K18R mutation (**Fig.4i-k**). Finally, comparison of H3.1/2-to-H3.3 ratios in wild-type and H3.1/2K18R cells by mass spectrometry showed increased H3.3 chromatin incorporation in the H3.1/2K18R cells, providing independent evidence for histone variant compensation (**Fig.4l**).

(11) The study assumes proper chromatin incorporation of mutant histones (K18R, K56R) without direct biochemical validation. We suggest discussing the different approaches (histone acid extraction, immunoblotting, mass spec) that could be used to confirm incorporation levels, which would clarify interpretation.

To verify that the mutant histones are expressed and incorporated into chromatin, we performed histone acid extraction and mass spectrometry analysis. Because free histones constitute less than 1% of the total histone pool in rapidly dividing cells, this method predominantly recovers chromatin-associated histones. Histone mass spectrometry profiling involves propionylation of lysine residues to block tryptic cleavage at lysines, thereby enforcing digestion at arginine only. Consequently, mutation of lysine to arginine introduces additional tryptic cleavage sites, resulting in novel peptides uniquely present in mutant samples. These mutant-specific peptides were detected in all K-to-R samples, confirming the presence of the modified histone variants within the chromatin fraction (**Extended Data Fig.6b**).

(12) In K9R mutants (differential changes between acetylation and methylation) they should check or discuss the impact of differential H3.3K9me/H3.1K9me and H3.1/H3.3K9ac.

The established partial K9R mutant lines display a strong tendency toward spontaneous differentiation in culture. We observed that the extent of H3K9ac loss in these clones varied substantially depending on cell collection density and the proportion of spontaneously differentiated cells. To avoid potential misinterpretation of these data, we therefore chose to remove the H3K9ac Western blot measurements from the manuscript. As suggested, we have added the discussion of the potential impact of differential H3.3K9me/H3.1K9me and H3.1/H3.3K9ac to the manuscript, which reads as follows:

"It will be informative to perform analogous analyses in partially edited H3K9R alongside H3.3K9R and combined H3.1/2/3K9R mutants to directly assess functional redundancy between variant and canonical PTMs at this site. Consistent with a model of context-dependent compensation between H3 variants with regard to H3K9 PTM function, histone mutagenesis studies in Drosophila have shown that H3.1/2 and H3.3 can compensate for one another with respect to H3K9ac[23]. In contrast, variant-specific requirements emerge in repressive contexts: H3.1/2K9 is uniquely required for repression of transposon-derived transcripts in pericentric heterochromatin, whereas H3.3K9 is necessary for maintenance of telomeric heterochromatin[23]. Together, these findings highlight separate targeting of H3.1/2 and H3.3 as a strategy for resolving both shared and variant-specific functions of histone modifications."

(13) When referencing future applications, such as modeling disease mutations (H3K27M, H3K36M),

the authors should cite papers such as (Schwartzentruber et al., Nature, 2012; Wu et al., Nature Genetics, 2012; Behjati et al., Nature Genetics, 2013) to ground the discussion.

We have incorporated these citations in the relevant segments of the Results and Discussion (lines 498 and 698).

(14) A brief sentence in the discussion projecting broader uses of the platform (editing other histone types (H4, H2A, H2B) or targeting other repetitive gene families) would help clarify the system's potential utility.

We have incorporated these suggestions in the last paragraph of the Discussion, which now reads as follows:

"Importantly, the methodology we have established can be extended beyond the analysis of direct histone H3 PTM effects. It also provides a means for investigating the functional roles of various residues on other canonical (H2A, H2B and H4) and non-canonical histone variants, both within individual organisms and across evolutionary contexts. This approach is also well-suited for studying oncohistones, which frequently involve single amino acid substitutions with strong effects on chromatin regulation[24–26]*. Moreover, the same strategy can be applied to targeted mutagenesis of other highly conserved or repetitive genomic elements, e.g., ubiquitin gene clusters, rRNA, tRNA genes and transposable elements, where conventional gene editing approaches face significant technical challenges."*

(15) While the Materials and Methods section is generally well organized, several important details are missing.

- The number of biological replicates is not consistently stated for key experiments.

The number of biological replicates is now stated in each figure legend and also in **Supplementary Table 3**.

- Statistical methods used to analyze and compare datasets are not described in the methods section.

We have provided a new **Supplementary Table 3**, which lists the statistical tests applied to each panel. We have also included the information on the statistical methods in each corresponding figure legend.

- The western blot methodology lacks detail: for example, on antibody validation, normalization protocol, and quantification tools.

The following text was added to the Materials and Methods to address this concern:

"Membranes were imaged using an Odyssey M flatbed scanner (Li-Cor Biosciences 3340-00). For each blot, two primary antibodies raised in different species were used: one targeting the histone modification of interest and one targeting the loading control (total H3). Species-specific secondary antibodies conjugated to distinct fluorophores (700 nm and 800 nm) were imaged in separate channels. Depending on the species of the primary antibody used to detect the target modification (mouse or rabbit), a total H3 antibody raised in the opposite species was used as the loading control to enable simultaneous dual-channel detection without cross-reactivity.

Antibody specificity was ensured by our SNAP-ChIP data, prior modification-specific validation reported in the literature or by vendors, as well as independent benchmarking available through the https://chromatinantibodies.com resource. Catalogue numbers and available validation information for all antibodies used in this study are provided in Supplementary Table 2.

For quantification, infrared fluorescence intensities were measured using Image Studio software (Li-Cor Biosciences). Rectangular regions of interest were applied across the bands, and local background

subtraction was performed using the software's standard median background method. Signal intensity for each target histone modification was normalised to the corresponding total H3 signal from the same lane. Normalised values were subsequently used for statistical analysis.

All Western blot experiments were independently repeated at least three times using independently collected cell pellets (biological replicates). Quantification from these biological replicates was consistent across blots and was statistically compared across all experimental conditions. The statistical tests utilised are provided in Supplementary Table 3 and the corresponding figure legends."

References

1. Mendiratta, S., Gatto, A. & Almouzni, G. Histone supply: Multitiered regulation ensures chromatin dynamics throughout the cell cycle. *J Cell Biol* **218**, 39–54 (2019).

2. Groth, A. *et al.* Regulation of Replication Fork Progression Through Histone Supply and Demand. *Science* **318**, 1928–1931 (2007).

3. Cook, A. J. L., Gurard-Levin, Z. A., Vassias, I. & Almouzni, G. A Specific Function for the Histone Chaperone NASP to Fine-Tune a Reservoir of Soluble H3-H4 in the Histone Supply Chain. *Mol. Cell* **44**, 918–927 (2011).

4. Goldberg, A. D. *et al.* Distinct Factors Control Histone Variant H3.3 Localization at Specific Genomic Regions. *Cell* **140**, 678–691 (2010).

5. Mito, Y., Henikoff, J. G. & Henikoff, S. Genome-scale profiling of histone H3.3 replacement patterns. *Nat. Genet.* **37**, 1090–1097 (2005).

6. Chen, P. *et al.* H3.3 actively marks enhancers and primes gene transcription via opening higher-ordered chromatin. *Genes Dev.* **27**, 2109–2124 (2013).

7. Schimmel, J. *et al.* Modulating mutational outcomes and improving precise gene editing at CRISPR-Cas9-induced breaks by chemical inhibition of end-joining pathways. *Cell Rep.* **42**, 112019 (2023).

8. Hwang, G.-H. *et al.* Large DNA deletions occur during DNA repair at 20-fold lower frequency for base editors and prime editors than for Cas9 nucleases. *Nat. Biomed. Eng.* **9**, 79–92 (2025).

9. Chen, P. J. & Liu, D. R. Prime editing for precise and highly versatile genome manipulation. *Nat Rev Genet* **24**, 161–177 (2023).

10. Espiritu, D. *et al.* Cancer histone mutations impact protein binding and DNA repair with possible links to genomic instability. *Nucleic Acids Res.* **53**, gkaf899 (2025).

11. Love, M. I., Huber, W. & Anders, S. Moderated estimation of fold change and dispersion for RNA-seq data with DESeq2. *Genome Biology* **15**, 550 (2014).

12. Aksoy, I. *et al.* Klf4 and Klf5 differentially inhibit mesoderm and endoderm differentiation in embryonic stem cells. *Nat. Commun.* **5**, 3719 (2014).

13. Zhang, P., Andrianakos, R., Yang, Y., Liu, C. & Lu, W. Kruppel-like Factor 4 (Klf4) Prevents Embryonic Stem (ES) Cell Differentiation by Regulating Nanog Gene Expression*. *J. Biol. Chem.* **285**, 9180–9189 (2010).

14. Zhao, H. *et al.* The chromatin remodeler Chd4 maintains embryonic stem cell identity by controlling pluripotency- and differentiation-associated genes. *J. Biol. Chem.* **292**, 8507–8519 (2017).

15. Zhu, F. *et al.* Sin3a–Tet1 interaction activates gene transcription and is required for embryonic stem cell pluripotency. *Nucleic Acids Res.* **46**, 6026–6040 (2018).

16. Molotkov, A., Mazot, P., Brewer, J. R., Cinalli, R. M. & Soriano, P. Distinct Requirements for FGFR1 and FGFR2 in Primitive Endoderm Development and Exit from Pluripotency. *Dev. Cell* **41**, 511-526.e4 (2017).

17. Kong, X. B. & Zhang, C. Dickkopf (Dkk) 1 promotes the differentiation of mouse embryonic stem cells toward neuroectoderm. *Vitr. Cell. Dev. Biol. - Anim.* **45**, 185–193 (2009).

18. Delaney, K., Weiss, N. & Almouzni, G. The cell-cycle choreography of H3 variants shapes the genome. *Mol. Cell* **83**, 3773–3786 (2023).

19. Chen, P. J. *et al.* Enhanced prime editing systems by manipulating cellular determinants of editing outcomes. *Cell* **184**, 5635-5652.e29 (2021).

20. Li, X. *et al.* Highly efficient prime editing by introducing same-sense mutations in pegRNA or stabilizing its structure. *Nat. Commun.* **13**, 1669 (2022).

21. Delaney, K., Weiss, N. & Almouzni, G. The cell-cycle choreography of H3 variants shapes the genome. *Mol. Cell* **83**, 3773–3786 (2023).

22. Goldberg, A. D. *et al.* Distinct Factors Control Histone Variant H3.3 Localization at Specific Genomic Regions. *Cell* **140**, 678–691 (2010).

23. Penke, T. J. R., McKay, D. J., Strahl, B. D., Matera, A. G. & Duronio, R. J. Functional Redundancy of Variant and Canonical Histone H3 Lysine 9 Modification in Drosophila. *Genetics* **208**, 229–244 (2018).

24. Wu, G. *et al.* Somatic histone H3 alterations in pediatric diffuse intrinsic pontine gliomas and non-brainstem glioblastomas. *Nat. Genet.* **44**, 251–253 (2012).

25. Schwartzentruber, J. *et al.* Driver mutations in histone H3.3 and chromatin remodelling genes in paediatric glioblastoma. *Nature* **482**, 226–231 (2012).

26. Behjati, S. *et al.* Distinct H3F3A and H3F3B driver mutations define chondroblastoma and giant cell tumor of bone. *Nat. Genet.* **45**, 1479–1482 (2013).

We thank the reviewers for taking the time to review the revised version of our manuscript and welcome their positive feedback on our work. No further revisions were requested.

Reviewer #1:
Remarks to the Author:
We think this is an excellent rebuttal and the authors have addressed our concerns. We do not require additional revision.

Our main request to improve rigor in point 2, where we had a problem with them conflating “we cannot make cell lines carrying mutations of this lysine” to “this lysine is essential” without direct demonstration. This was also raised by reviewer #3. They made a good faith effort to do the requested experiment, which did not work and so instead softened their language. We think this is acceptable.

With respect to novel biological significance, the paper’s contribution is their identification of which H3 lysines are essential vs. dispensable for mESC self-renewal. It will probably be cited often for this point, so we acknowledge that this is good enough.

We appreciate the double mutant data they introduce to our point 7, including the RNA-seq analysis and their pointing out the transcriptome rewiring that appears (or is reversed) only in the double mutants. They do a good job arguing that it demonstrates biological significance.

Reviewer #3:
Remarks to the Author:
We thank the authors for their detailed responses. They have made a substantial effort to address our concerns by adding appropriate controls and quantifications, and by performing additional experiments requested by all reviewers (including sequential mutagenesis, double-mutant RNA-seq, CUT&Tag profiling, and mass spectrometry), which significantly strengthen the manuscript.

The new data further support the main conclusion . Interestingly, it shows that combinatorial perturbations (such as H3K27R+H3K36R double mutant) show impairments in self - renewal , whereas the corresponding single substitutions have only modest effects. This work highlights unique regulatory crosstalk that would not be evident from single-site perturbations alone.

The revised Discussion now provide a proper contextualization of the findings and attention has been paid to avoid overstatement.

In conclusion, we believe this work will serve as a foundation for future efforts to map and manipulate the histone code. Therefore, we support its publication in Nature Genetics.
